# Plant-Derived Catechols Are Substrates of TonB-Dependent Transporters and Sensitize Pseudomonas aeruginosa to Siderophore-Drug Conjugates

**DOI:** 10.1128/mbio.01498-22

**Published:** 2022-06-30

**Authors:** Alexandre Luscher, Véronique Gasser, Dirk Bumann, Gaëtan L. A. Mislin, Isabelle J. Schalk, Thilo Köhler

**Affiliations:** a Service of Infectious Diseases, University Hospital Geneva, Geneva, Switzerland; b Department of Microbiology and Molecular Medicine, University of Geneva, Geneva, Switzerland; c CNRS, University of Strasbourg, Strasbourg, France; d Biozentrum, University of Basel, Basel, Switzerland; Laboratory of Microbiology Signals and Microenvironment LMSM EA 4312; Harvard Medical School

**Keywords:** *Pseudomonas aeruginosa*, polyphenols, siderophore-drug conjugate, drug transport, plant polyphenols, siderophores

## Abstract

Pseudomonas aeruginosa is an opportunistic pathogen responsible for acute and chronic infections in immunocompromised hosts. This organism is known to compete efficiently against coinfecting microorganisms, due in part to the secretion of antimicrobial molecules and the synthesis of siderophore molecules with high affinity for iron. P. aeruginosa possess a large repertoire of TonB-dependent transporters for the uptake of its own, as well as xenosiderophores released from other bacteria or fungi. Here, we show that P. aeruginosa is also capable of utilizing plant-derived polyphenols as an iron source. We found that exclusively plant-derived phenols containing a catechol group (i.e., chlorogenic acid, caffeic acid, quercetin, luteolin) induce the expression of the TonB-dependent transporters PiuA or PirA. This induction requires the two-component system PirR-PirS. Chlorogenic acid in its Fe(III)-loaded form was actively transported by PiuA and PirA and supported growth under iron-limiting conditions. Coincidentally, PiuA and PirA are also the main TonB transporters for the recently approved siderophore-drug conjugate cefiderocol. Surprisingly, quercetin supplementation increased the susceptibility of P. aeruginosa to siderophore-drug conjugates, due to induction of *piuA* and *pirA* expression mediated by the PirR-PirS two-component system. These findings suggest a potential novel therapeutic application for these biologically active dietary polyphenols.

## INTRODUCTION

Pseudomonas aeruginosa is a ubiquitous Gram-negative bacterium that dwells in humid environments rich in organic substrates but is also a nosocomial pathogen able to cause severe acute and chronic infections in hospitalized patients, as well as in cystic fibrosis patients (1). It is well known that its adaptability to various environmental conditions is due to a huge repertoire of metabolic pathways. P. aeruginosa is a pathogen for plant, animal, and human hosts due to common or specific virulence determinants, including quorum-sensing controlled secreted products and several contact-dependent secretion systems (type III secretion system [T3SS], T5SS, and T6SS) (2–4). Survival under restricted nutrient conditions and the capacity to outcompete other microorganisms is in part due to the production and secretion of efficient iron-scavenging molecules, namely, the siderophores pyoverdine and pyochelin (5–7). Both molecules are produced under iron-deficient conditions, and pyoverdine is essential for full virulence of P. aeruginosa in various animal models (8). The import across the outer membrane of siderophore-Fe complexes in Gram-negative bacteria occurs via TonB-dependent transporters (9–11). P. aeruginosa harbors 35 TonB-dependent transporter genes on its chromosome, three of which are dedicated to the transport of the endogenous siderophores pyoverdine (FpvA, FpvB) and pyochelin (FptA) (12–14). This large diversity of TonB-dependent transporters endows P. aeruginosa with the ability to use a variety of xenosiderophores produced by other microorganisms, which represents a competitive advantage when thriving in the presence of other bacteria in iron-deficient environments. Unlike the diffusion across the rather nonspecific porin proteins in the outer membrane of Gram-negative bacteria, the uptake of siderophore-Fe complexes via TonB transporters is a substrate-specific, active process requiring the inner membrane complex ExbBD-TonB, which transmits to the outer membrane transporter energy derived from proton gradient across the inner membrane (15, 16). The TonB protein contacts the N terminus of the plug domain of the TonB-dependent transporters and promotes uptake of the substrate recognized by the surface exposed domain of the plug buried within the β-barrel domain (17–22).

Among the 35 TonB transporters, a subgroup has been associated with the uptake of specific xenosiderophores (23), namely, PfeA (enterobactin from Escherichia coli) (24), PirA (catecholamines) (10), FemA (mycobatin from mycobacteria) (25), FvbA (vibriobactin from Vibrio cholerae) (26), FiuA (ferrichrome from fungi and desferioxamine) (27, 28), FoxA (nocardamine from Streptomyces) (28), FecA for citrate (29), and ChtA (aerobactin from enterobacteriaceae) (30). Three TonB transporters (HasR, PhuR, and HxuC) are involved in the uptake of heme and hemophores, while other transporters promote the uptake of non-iron transition metals (OprC, BtuB, and ZnuD) (31). The expression of TonB transporters in P. aeruginosa, involved in iron uptake, is tightly regulated by the global iron regulator Fur (32). We previously showed that ion complexation induced expression of 18 of the 35 TonB-dependent transporter proteins (33), indicating their involvement in cation transport. The transcription of some of these TonB-dependent transporters is also positively regulated by sigma/anti-sigma factors (34, 35), two-component systems (36), and transcriptional regulators of the AraC family (37). When sigma factors are involved, the extracellular siderophores bind to the plug domain of the TonB-dependent transporter, which undergoes a conformational change that is transmitted to the anti-sigma factor leading to activation of an extracytoplasmic function (ECF) sigma factor, which in turn alters the transcription of a subset of genes (34, 35, 38). Most of the known ECF sigma factors in P. aeruginosa are adjacent to iron acquisition systems (25, 39), and only two siderophore TonB transporter genes are located next to a two-component signaling system (TCS) on the chromosome, namely, *pfeA* adjacent to *pfeR*-*pfeS* (36) and *pirA* adjacent to *pirR*-*pirS* (6).

Here, we show that P. aeruginosa is able to sense plant-derived phenols with Fe-complexing capacities. These phenols are recognized by the PirR-PirS TCS and transported via the PiuA and PirA TonB-dependent transporters. Since PiuA and PirA are also the main uptake pathways for therapeutically used siderophore-drug conjugates (21, 40, 41), upregulation of these transporters by plant phenols renders P. aeruginosa more susceptible to these Trojan horse antimicrobials.

## RESULTS

### Constitutive and inducible expression of *piuA* and *pirA* in P. aeruginosa.

**FIG 1:**
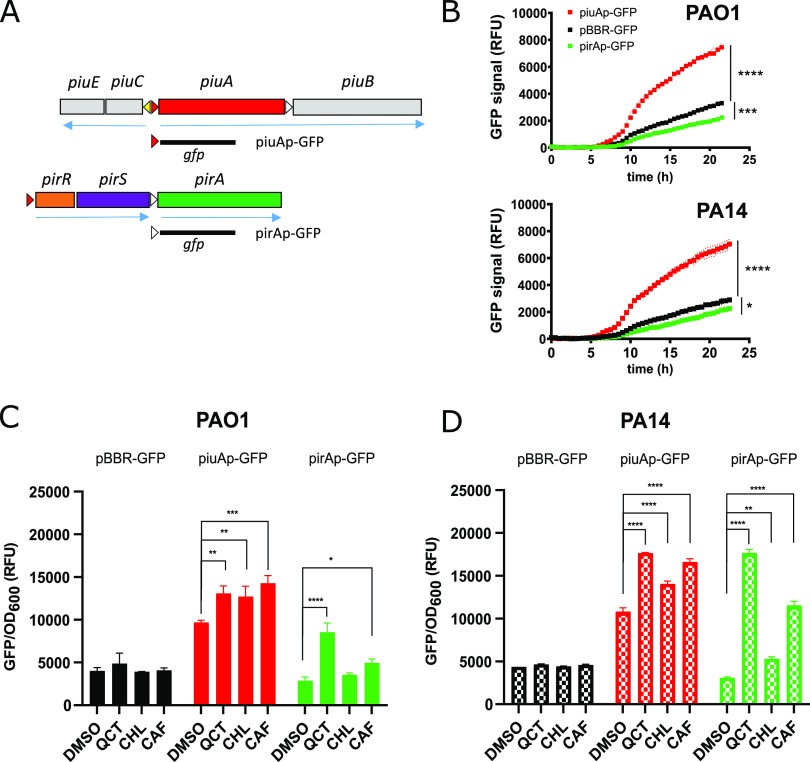
Expression of *piuA* or *pirA* is induced in the presence of plant catechols. (A) Operon structure of the *piuA* and *pirA* loci of P. aeruginosa. PAO1 and PA14 carry the *piuA* gene. Experimentally verified Fur boxes (red triangles) are present in *piuA* and *pirR* promoter regions, while the *piuC* promoter contains a putative Fur box (yellow arrowhead). (B) Fluorescence emitted from the vector pBBR–green fluorescent protein (GFP) and the piuAp-GFP and pirAp-GFP transcriptional fusions, represented as relative fluorescent units (RFU), was monitored during growth in M9-CAA medium in PAO1 and PA14. (C, D) Effect of plant phenols on expression of *pirA* and *piuA* expression in PAO1 (C) and PA14 (D). Quercetin (QCT), chlorogenic acid (CHL), and caffeic acid (CAF) were added at final concentrations of 40 μM. The data are represented as means and SEM of biological duplicates. Statistical significance was assessed using two-way analysis of variance (ANOVA) and a Dunnett’s test with pBBR-GFP (B) or dimethyl sulfoxide (DMSO) (C, D) as a control. *, *P* < 0.05; **, *P* < 0.01; ***, *P* < 0.005; ****, *P* < 0.001. More detailed information can be found in Fig. S1.

We and others previously showed that PiuA and PirA are the main TonB-dependent transporters, which under standard drug susceptibility testing conditions were responsible for the uptake of siderophore-drug conjugates, including the FDA-approved cefiderocol (21, 40, 41). To study their regulation and identify potential substrates of these TonB transporters, we constructed transcriptional fusions with the promoter regions of the *pirA* and *piuA* genes in the promoter probe vector pBBR–green fluorescent protein (GFP), yielding plasmids pirAp-GFP and piuAp-GFP, respectively (Fig. 1A). The promoter regions of *pirR* and *piuA* contain previously described Fur-box motifs similar to the canonical P. aeruginosa sequence GATAATGATAATCATTATC (red arrows in Fig. 1A) (42). While no Fur-box motifs were present (>10 of 19 nucleotide matches with consensus sequence) upstream of the *pirA* and *piuB* genes (white arrows in Fig. 1A), we identified a novel putative Fur-box motif in the *piuC* upstream region (GCAAATCAATATCATTGGC; yellow arrow in Fig. 1A). We next introduced plasmids pirAp-GFP and piuAp-GFP into the PAO1 and PA14 wild-type strains and followed fluorescence emission during growth in the iron-limiting M9-CAA medium (Fig. 1B). While fluorescence emission from the pirAp-GFP fusion was below the vector control during the 23 h of the incubation period, the piuAp-GFP fusion showed a 2-fold higher expression level than the vector (Fig. 1B) in both PAO1 and PA14. Hence, we conclude that *pirA* is not expressed in the absence of an inducer, while *piuA* shows constitutive expression in both wild-type strains.

10.1128/mbio.01498-22.6 FIG S1 Induction of *piuA* and *pirA* by plant-derived phenols in PAO1. The strains were grown in M9-CAA medium in the presence or absence of 40 μM plant phenols or DMSO as vehicle control. When added in combination quercetin and chlorogenic acid were provided at 20 μM each. The data are the ratio of relative fluorescence units (RFU) divided by the optical density at 600 nm (OD_600_) after 23 h of incubation at 37°C. The dotted lines correspond to baseline expression of the vector control exposed to DMSO. The values represent the average and SD of two biological replicates. One-way ANOVA analysis shows statistically significant differences compared to the DMSO control condition. *, *P* < 0.05; **, *P* < 0.01; ***, *P* < 0.005; ****, *P* < 0.001. Download FIG S1, PDF file, 0.03 MB. Copyright © 2022 Luscher et al. 2022 Luscher et al. https://creativecommons.org/licenses/by/4.0/ This content is distributed under the terms of the Creative Commons Attribution 4.0 International license.

### Plant phenols induce the expression of TonB-dependent transporters.

**FIG 2:**
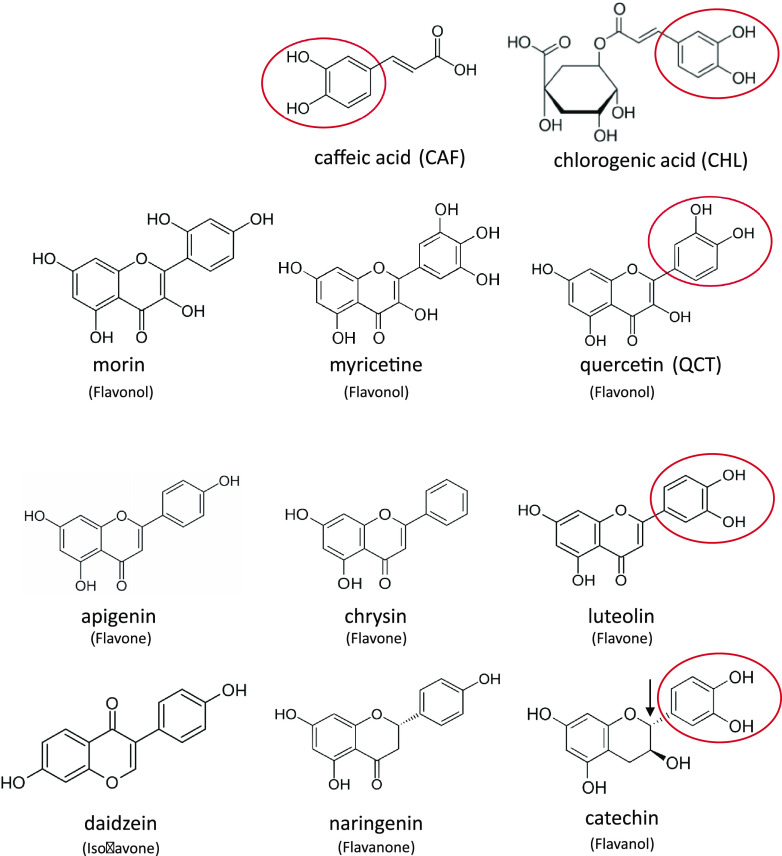
Plant-derived catechols induce *piuA* or *pirA* expression. Chemical structures of plant compounds used in this study. Chlorogenic acid is an ester of quinic acid and caffeic acid, which are intermediates of lignin biosynthesis. Polyphenols tested belong to different flavonoid families. Only compounds harboring a catechol group (pink circle) induce *piuA* or *pirA* TonB transporter genes.

To search for inducers and hence potential substrates of the PiuA and PirA transporters, we investigated natural compounds including plant-derived phenols, like the lignin biosynthetic intermediates caffeic acid and chlorogenic acid, as well as polyphenols (Fig. 2). Polyphenols have antioxidant properties, with some also displaying weak antimicrobial activities (43, 44). They are produced in leaves and roots and protect plants against UV radiation and stress-induced oxygen radicals. We screened the *piuA* and *pirA* promoter-GFP fusions in the presence of 40 μM commercially available plant phenols for their ability to induce expression of these genes in PAO1. Among 11 compounds tested, only caffeic acid, chlorogenic acid, quercetin, and luteolin showed increases in *piuA* and/or *pirA* expression in PAO1 (Fig. S1). The two lignin biosynthetic intermediates chlorogenic acid and caffeic acid, and the polyphenols quercetin and luteolin showed a weak induction of *piuA*, while quercetin induced preferentially the *pirA* promoter (Fig. 1C and D; Fig. S1). The response of *pirA* to quercetin and caffeic acid was more pronounced in PA14 compared to PAO1 (Fig. 1C and D). This was confirmed by the gene expression kinetics showing a higher induction of *pirA* by quercetin, chlorogenic acid, and caffeic in PA14 compared to PAO1 (Fig. S2). We also performed real-time quantitative PCR (qRT-PCR) in the presence of these compounds under identical growth conditions. The results confirmed induction of *pirA* by quercetin in PAO1 and also by chlorogenic acid and caffeic acid in PA14 (Fig. S3). Induction ratios were higher in PA14 compared to PAO1, as observed with the GFP fusions (Fig. 1C and D; Fig. S2).

10.1128/mbio.01498-22.7 FIG S2 Expression kinetics of *piuA* and *pirA* green fluorescent protein (GFP) fusions in PAO1 and PA14. Strains were grown in M9-CAA medium in the presence or absence of 40 μM plant phenols or DMSO as vehicle control. The data show the fluorescence emission (RFU) measured in a plate reader (BioTek Synergy H1; excitation, 485 nm/emission, 528 nm). The data represent the means and SD of two technical replicates. Download FIG S2, PDF file, 0.04 MB. Copyright © 2022 Luscher et al. 2022 Luscher et al. https://creativecommons.org/licenses/by/4.0/ This content is distributed under the terms of the Creative Commons Attribution 4.0 International license.

10.1128/mbio.01498-22.8 FIG S3 Induction of *piuA* and *pirA* by plant-derived catechols in PAO1 and PA14 by qRT-PCR. The strains were grown in microtiter plates in M9-CAA medium in the presence or absence of 40 μM plant phenols or DMSO as vehicle control. The values show the fold change in *piuA*/*rpsL* and *pirA*/*rpsL* expression ratios in response to phenolic compounds and represent the average and SD of three biological replicates. Two-way ANOVA analysis shows statistically significant differences compared to the DMSO control condition. *, *P* < 0.05; **, *P* < 0.01; ***, *P* < 0.005; ****, *P* < 0.001. QCT, quercetin; CHL, chlorogenic acid; CAF, caffeic acid; ND, not done. Download FIG S3, PDF file, 0.01 MB. Copyright © 2022 Luscher et al. 2022 Luscher et al. https://creativecommons.org/licenses/by/4.0/ This content is distributed under the terms of the Creative Commons Attribution 4.0 International license.

By comparing the chemical structures of the tested molecules, we noticed that the inducers differ from those of the noninducing compounds by the presence of a catechol moiety in the former group (Fig. 2, red ovals). An exception was catechin, which did not affect *piuA* or *pirA* expression, despite the presence of a catechol group (Fig. S1). However, this catechol moiety lacks the planar configuration of the other compounds due to the presence of a chiral carbon atom to which the catechol group is attached (arrow in catechin structure in Fig. 2). Our data establish for the first time a link between plant-derived compounds carrying a catechol group, present in many bacterial siderophores (pyoverdine, enterobactin, azotochelin, etc.), and the induction of TonB-dependent transporter genes in P. aeruginosa.

### Plant phenols sensitize P. aeruginosa to siderophore-drug conjugates.

Since PiuA and PirA are the main transporters for the uptake of the siderophore-drug conjugates BAL30072, MC-1, and cefiderocol (33, 40, 41), we wondered whether plant-derived phenols would also affect susceptibility to these conjugates. We thus performed MIC determinations with the siderophore-drug conjugate BAL30072 in the presence and absence of plant phenols. Interestingly, we observed an 8-fold increase in susceptibility to BAL30072 in the presence of quercetin and chlorogenic acid, while luteolin and caffeic acid showed 4- and 2-fold increases in susceptibility, respectively (Table 1). We suspected that the increased expression of *piuA* and/or *pirA* in response to plant phenols would lead to an increase in the amount of the cognate TonB transporters and hence increased uptake of siderophore-drug conjugates. Indeed, we observed a good correlation between induction of *piuA*/*pirA* and increased susceptibility to BAL30072 (Table 1). Plant phenols did not affect MICs of aztreonam, a nonsiderophore monobactam. Notably, simultaneous addition of both chlorogenic acid and quercetin resulted in a further increase in susceptibility to BAL30072, which was corroborated by a strong induction of *pirA* expression (Table 1; Fig. S1). To assess that plant phenols did not cause a general permeabilization of the bacterial membrane, we performed MIC determinations with a set of antibiotics including tobramycin, ciprofloxacin, tetracycline, azithromycin, and polymyxin. None of the catechols tested affected the susceptibilities of PAO1 to these antibiotics (Table S1), indicating that the effect is specific to the siderophore-drug compounds, which are substrates of the PiuA and PirA TonB-dependent transporters.

**TABLE 1:** Effect of polyphenols on TonB receptor expression and antibiotic susceptibility in PAO1^*a*^

Plant phenols	Induction		MIC (μg/mL)	
	Of *piuA*	Of *pirA*	BAL30072	AZM
None (DMSO)	NC	NC	1	4
Chlorogenic acid	+	+	**0.125**	4
Caffeic acid	+	+	0.5	4
Quercetin	+	++	**0.125**	4
Luteolin	+	+	**0.25**	4
Chlorogenic acid + quercetin^*b*^	+	+++	**0.06**	4
Morin	NC	NC	0.5	4
Myricetin	NC	NC	0.5	4
Apigenin	NC	NC	1	2
Chrysin	NC	NC	1	4
Daidzein	NC	NC	1	4
Naringenin	NC	NC	0.5	4
Catechin	NC	NC	1	2
Genistein	NC	NC	1	4
Sinigrin	NC	NC	1	2

a Plant phenols were tested at 40 μM final concentration for the induction and 20 μM for MIC determinations. The values in bold represent at least four-fold changes in MIC compared to control. AZM, aztreonam; NC, no change in expression. +, weak, ++ medium, +++ strong increase in expression.

b At 10 μM final concentration each.

10.1128/mbio.01498-22.1 TABLE S1 Effect of plant phenols on antibiotic susceptibility of PAO1. Plant phenols affect only the MICs of the siderophore-drug conjugate BAL30072 but not those of other antibiotics, suggesting that the plant phenols are not general membrane permeabilizers. Download Table S1, PDF file, 0.08 MB. Copyright © 2022 Luscher et al. 2022 Luscher et al. https://creativecommons.org/licenses/by/4.0/ This content is distributed under the terms of the Creative Commons Attribution 4.0 International license.

### Quercetin and chlorogenic acid affect growth in the absence of endogenous siderophores.

Next, we compared the growth of the pyoverdine- and pyochelin-deficient strain PA14Δ*pvdpch* with derivatives in which we deleted the *pirA* and *piuA* TonB-dependent transporter genes. We reasoned that in a strain unable to produce these endogenous siderophores, PiuA and PirA might import iron via plant catechols and promote growth under iron-limited conditions.

**FIG 3:**
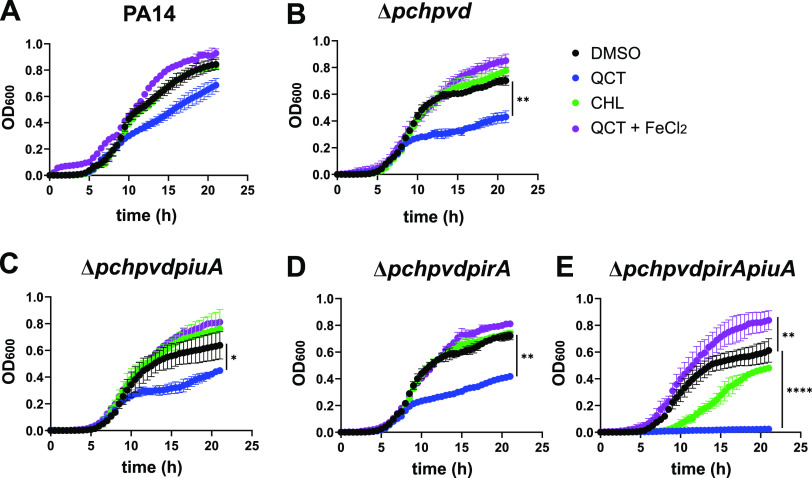
Effect of plant catechols on growth under Fe-limiting conditions. The strains were grown without agitation at 37°C in M9-CAA medium in microtiter plates. Growth (optical density at 600 nm [OD_600_]) was monitored every 30 min. Quercetin (QCT) and chlorogenic acid (CHL) were added at a final concentration of 40 μM, and FeCl_2_ was added at a final concentration of 10 μM. The data represent the mean and standard deviation of biological duplicates. (A) PA14. (B) *Δpchpvd*. (C) *ΔpchpvdpiuA*. (D) *ΔpchpvdpirA*. (E) *ΔpchpvdpirApiuA*. Statistics were determined by two-way ANOVA and Dunnett’s test with DMSO control. *, *P* < 0.05; **, *P* < 0.01; ***, *P* < 0.005; ****, *P* < 0.001.

Quercetin delayed growth compared to the dimethyl sulfoxide (DMSO) control condition in all the PA14-derived mutant strains (blue versus black lines in Fig. 3A to E). Markedly, quercetin completely prevented growth of the Δ*pvdpchpirApiuA* mutant, suggesting that iron-loaded quercetin cannot enter the cell in the absence of the PiuA and PirA transporters (Fig. 3E). On the other hand, chlorogenic acid did not affect growth of PA14 and its Δ*pvdpch* derivative (green versus black lines in Fig. 3A and B) but slightly improved growth of the Δ*pvdpchpiuA* mutant compared to the DMSO control (green versus black lines in Fig. 3C, difference not significative). This growth-promoting effect was abrogated in the Δ*pvdpchpirA* and the Δ*pvdpchpirApiuA* mutants (green versus black lines in Fig. 3D and E). We thus conclude that chlorogenic acid behaves as a bona fide siderophore, recognized and transported mainly by PirA. The addition of 10 μM Fe(II), whose uptake is independent of TonB transporters, restored growth retardation imposed by quercetin in all the strains tested (purple versus blue lines in Fig. 3A to E). The data strongly suggest that both quercetin and chlorogenic acid transport Fe(III) via the TonB-dependent transporters PirA and PiuA.

### Chlorogenic acid is transported by PiuA and PirA.

**FIG 4:**
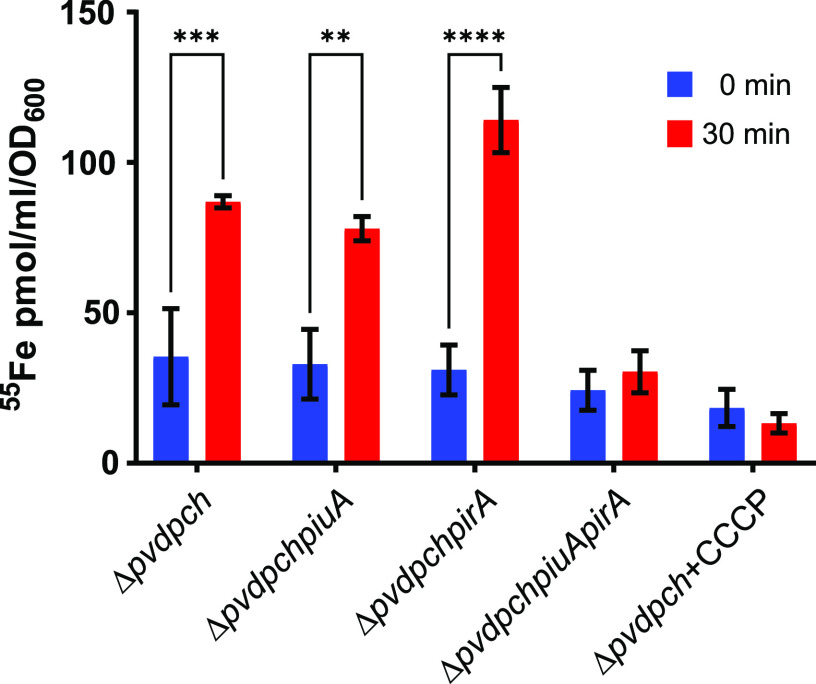
Uptake of chlorogenic acid loaded with ^55^Fe(III). 500 nM chlorogenic acid was added in a 200:1 ratio with ^55^Fe(III)Cl_3_. Uptake was measured after 30 min in PA14-derived mutants. The data show the means and SEMs of two independently performed experiments. Comparisons were made using two-way ANOVA and Sidák’s test. **, *P* < 0.01; ***, *P* < 0.005; ****, *P* < 0.001. CCCP, carbonyl cyanide *m*-chlorophenylhydrazone.

To verify this hypothesis, we measured the uptake of ^55^Fe(III)-loaded quercetin and chlorogenic acid in whole cells of P. aeruginosa strain PA14Δ*pvdpch*. We chose again the pyoverdine- and pyochelin-deficient strain background to avoid interference with Fe(III) transport by these endogenous siderophores. Unfortunately, precipitation of quercetin-^55^Fe was observed under the conditions required for the ^55^Fe-uptake assay, resulting in radioactive background noise, precluding ^55^Fe-uptake measurements with quercetin. In contrast, chlorogenic acid was soluble, and we measured accumulation of ^55^Fe(III) in strain PA14Δ*pvdpch* during the 30 min incubation period in the presence of chlorogenic acid (Fig. 4), but not in its absence (data not shown). When the TonB-dependent transporter genes *piuA* and *pirA* were mutated individually, ^55^Fe(III) still accumulated to the same level as in the parental strain PA14Δ*pvdpch*. However, when *piuA* and *pirA* genes were deleted simultaneously, no ^55^Fe accumulation was observed in the presence of chlorogenic acid. TonB-dependent transport requires energy derived from the proton gradient across the cytoplasmic membrane. Hence, the addition of the ionophore carbonyl cyanide *m*-chlorophenylhydrazone (CCCP), which dissipates the proton gradient, abrogated ^55^Fe accumulation in the presence of chlorogenic acid (Fig. 4). These results confirm that Fe-loaded chlorogenic acid is translocated via PirA and/or PiuA in a proton gradient-driven transport process.

### Plant catechols signal through the PirR-PirS TCS.

**FIG 5:**
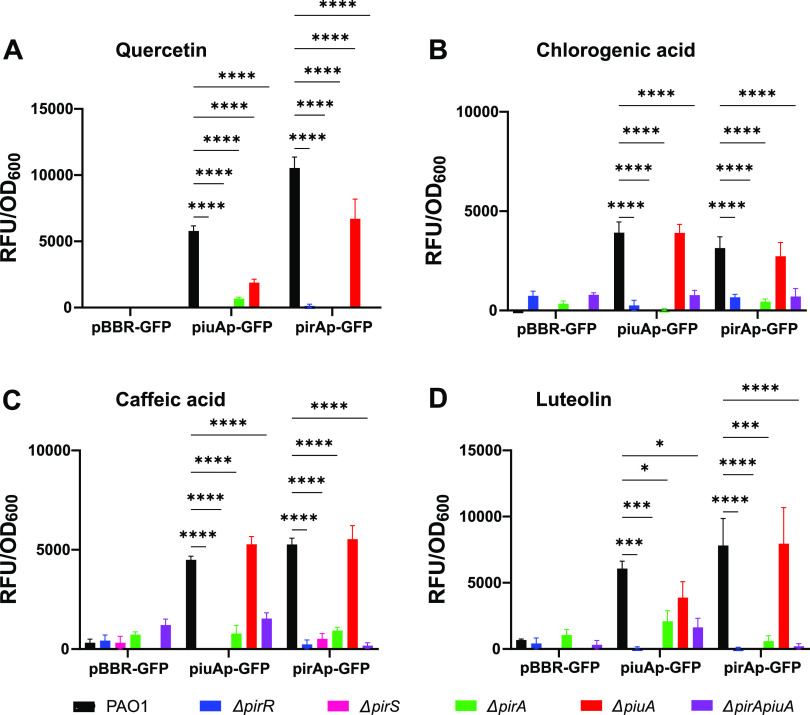
Induction of *piuA* and *pirA* by catechols requires the PirR-PirS TCS and the PirA transporter. The strains were grown in M9-CAA medium in the presence or absence of 40 μM inducer (20 μM for luteolin). The data show the difference (inducer – DMSO) in normalized fluorescence emission after 23 h of growth at 37°C for the vector control pBBR-GFP and the transcriptional fusions piuAp-GFP and pirAp-GFP. The data represent the averages and SEMs of three independently performed experiments. (A) Quercetin. (B) Chlorogenic acid. (C) Caffeic acid. (D) Luteolin. Significance was determined by two-way ANOVA with Dunnett’s comparison to DMSO control.

**FIG 6:**
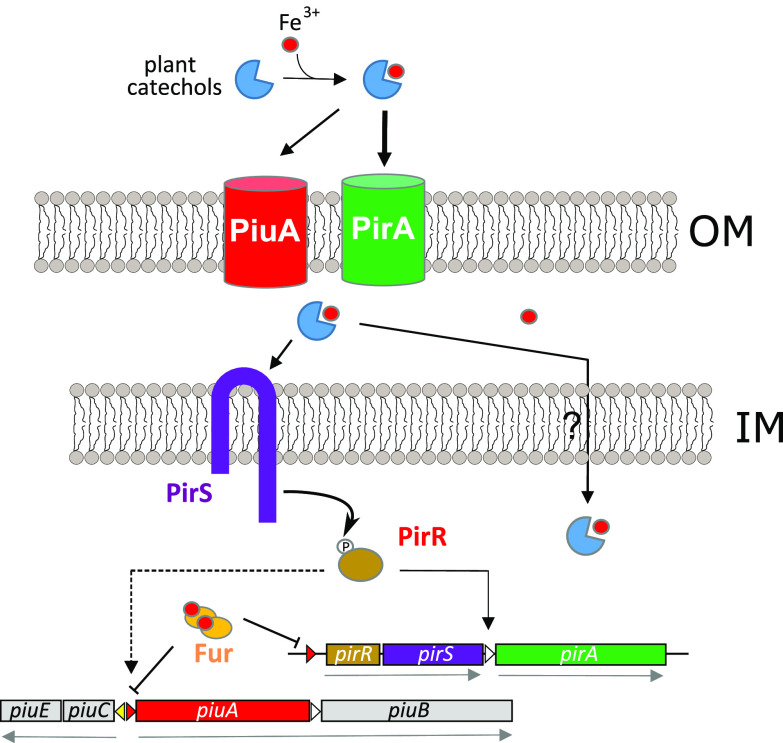
Proposed model for the regulation of *piuA* and *pirA* operons. Iron-loaded quercetin and other plant catechols are transported via PiuA and PirA. Either iron is released in the periplasm or the complex may be further transported across the inner membrane to the cytosol. Plant catechols interact with PirS in the periplasm, leading subsequently to PirR phosphorylation, which induces expression of *piuA* and *pirA*. Under iron-proficient conditions, Fur represses expression of both operons by binding to Fur boxes upstream *pirR* and *piuA* (red arrowheads). IM, inner membrane; OM, outer membrane.

Since the PirA TonB-dependent transporter gene is located downstream of two ORFs, annotated as two-component response regulator *pirR* and two-component sensor *pirS* on the P. aeruginosa chromosome (pseudomonas.com), we wondered whether the plant catechols would be recognized by this TCS (Fig. 1A). We therefore generated defined deletion mutants of the *pirR* and *pirS* genes in strain PAO1 and measured the expression of the pirAp-GFP and piuAp-GFP fusions in response to the catechol inducer molecules and subtracted the corresponding values for the vehicle DMSO. Compared to the wild-type PAO1, expression levels of *piuA* and *pirA* dropped by at least 10-fold, for all four catechol molecules, in the Δ*pirR* and Δ*pirS* mutants, suggesting that all four catechols signal via the PirR-PirS TCS (Fig. 5A to D). As expected, expression of both *piuA* and *pirA* was significantly decreased in the presence of quercetin, when *piuA* or *pirA* genes were deleted (Fig. 5A), in agreement with quercetin being transported via PiuA and PirA. In the presence of the three other catechols, only deletion of *pirA* significantly decreased *piuA* and *pirA* expression, while deletion of *piuA* did not affect significantly their expression (Fig. 5B to D). These data support the notion that quercetin is transported preferentially by PirA and less by PiuA, while the other three catechols seem to be exclusively transported via PirA. Once in the periplasmic space, all four catechols signal via the PirR-PirS TCS, since induction of *piuA* and *pirA* by all four catechols requires the PirS sensor kinase and the PirR response regulator as detailed schematically in Fig. 6.

Since PirR-PirS control expression of *piuA* and *pirA*, we hypothesized that this TCS might also determine the susceptibility to siderophore-drug conjugates. We therefore measured the susceptibility to BAL30072 in the Δ*pirR* and Δ*pirS* mutants in response to quercetin. Surprisingly, in the absence of quercetin the Δ*pirR*, but not the Δ*pirS* mutant, showed 4-fold higher BAL30072 MICs than the wild type (Table 2). Since *pirA* is not expressed in the PAO1 and PA14 wild-type strains (Fig. 1B), the most likely explanation is that PirR directly or indirectly controls constitutive expression of *piuA*. In the presence of quercetin, PAO1 and the Δ*pirA* mutant display the same reduced BAL30072 MICs (0.125 mg/liter), which likely result from increased uptake of BAL30072 due to quercetin-induced upregulation of PiuA (Fig. 1B; Table 2). As expected, deletion of the *pirS* sensor kinase gene increased BAL30072 MICs only in the presence of quercetin, demonstrating the requirement of PirS for quercetin recognition and signal transduction. As reported previously, deletion of *pirA* had no effect on BAL30072 MICs (40, 41); however, deletion of *piuA* increased MICs in the presence and absence of quercetin (Table 2). Finally, the identical elevated BAL30072 MICs (16 mg/liter) observed for the Δ*piuApirA* double mutant in the presence and absence of quercetin are in support of an exclusive uptake of quercetin via these two TonB transporters.

**TABLE 2:** Effect of *pirR* and *pirS* deletion on susceptibility of PAO1 to BAL30072^*a*^

Strain	MIC (μg/mL)			
	MHB		MHB + 20 μM quercetin	
	BAL30072	AZM	BAL30072	AZM
PAO1	1	4	0.125	4
Δ*pirR*	4	4	2	4
Δ*pirS*	1	4	0.5	4
Δ*pirA*	1	4	0.125	4
Δ*piuA*	8	4	2	4
Δ*piuApirA*	16	4	16	4

*^a^*MHB, Mueller-Hinton broth; AZM, aztreonam.

### Cross-talk between the *piu* operon and the PirR-PirS TCS.

The unexpected finding of *piuA* regulation by the PirR response regulator let us hypothesize that the level of PirR might impact the expression level of the PiuA TonB-dependent transporter. We therefore constructed plasmids ppirR and ppirS, providing constitutive expression of these genes, and introduced them into strain PAO1. While plasmid ppirS did not affect activity of the siderophore-drug conjugates, expression of PirR from plasmid ppirR decreased BAL30072 MICs 4-fold and those of MC-1 and cefiderocol 16-fold, which could result from increased expression of PiuA or PirA (Table 3). We therefore introduced the ppirR plasmid in the Δ*pirA* and Δ*piuA* mutants. While *pirR* expression still decreased BAL30072 MICs in the Δ*pirA* mutant, no effect on BAL30072 susceptibilities was observed in the Δ*piuA* mutant. We conclude from these data that PirR directly or indirectly controls the basal level expression of *piuA*. To corroborate this hypothesis at the protein level, we performed a proteomic analysis on PA14 and its isogenic Δ*pirR* mutant grown under standard MIC conditions. Among the 2,384 proteins detected by mass spectrometry analysis in both strains (at least two peptide counts per protein), only three proteins showed significantly decreased amounts in the Δ*pirR* mutant compared to PA14, namely, PiuA (19-fold), PiuB (2-fold), and OprH (2-fold) (Table S2). These results support a specific control of PirR on the basal level expression of PiuA.

**TABLE 3:** Effect of *pirR* and *pirS* overexpression on siderophore-drug conjugate activities^*a*^

Strain	MIC (μg/mL)			
	BAL30072	MC-1	CEF	AZM
PAO1 + pIApX2 (vector)	1	0.25	0.5	2
PAO1 + *ppirR*	**0.25**	**0.01**	**0.03**	4
PAO1 + *ppirS*	0.5	0.25	0.5	4
Δ*pirA* + pIApX2	1	0.25	0.5	4
Δ*pirA* + *ppirR*	**0.25**	**0.03**	**0.03**	4
Δ*piuA* + pIApX2	8	8	8	4
Δ*piuA* + *ppirR*	8	4	8	4

a MHB supplemented with carbenicillin at 100 μg/mL final concentration; CEF, cefiderocol; AZM, aztreonam;Values in bold show MIC values differing by at least 4-fold to those of the corresponding vector control.

10.1128/mbio.01498-22.2 TABLE S2 Proteomic analysis of PA14 and its Δ*pirR* mutant. Among the 2,384 proteins identified by mass spectrometry (with at least two peptide counts), only three proteins (PiuA, PiuB, and OprH) showed significantly decreased abundances in the Δ*pirR* mutant compared to PAO1, suggesting that PirR directly or indirectly positively regulates expression of PiuA. Download Table S2, PDF file, 1.4 MB. Copyright © 2022 Luscher et al. 2022 Luscher et al. https://creativecommons.org/licenses/by/4.0/ This content is distributed under the terms of the Creative Commons Attribution 4.0 International license.

## DISCUSSION

In the host or in the environment, P. aeruginosa is exposed to iron-limiting conditions. P. aeruginosa strains produce two endogenous siderophores, pyoverdine and pyochelin, transported by dedicated TonB-dependent transporters (FpvA, FpvB, and FptA). During evolution, P. aeruginosa has probably acquired and maintained on its chromosome additional TonB-dependent transporters able to import xenosiderophores produced by other microorganisms. Here, we show that the two TonB-dependent transporters PiuA and PirA are responsible for the uptake of plant-derived catechols. Coincidently, PiuA and PirA are also the main transporters for siderophore-drug conjugates and are among 18 TonB transporters induced in P. aeruginosa under iron-limiting conditions (33). Due to the antimicrobial activity of the siderophore-drug conjugates, it remains unclear whether PiuA and PirA are induced by these drugs. We further show that plant-derived catechols, but not compounds showing a different configuration of the hydroxyl groups on the same scaffold, are inducers of *piuA* and *pirA* genes. Expression of *pirA* and *piuA* in response to the catechol compounds caffeic acid and chlorogenic acid, as well as the polyphenolic quercetin and luteolin, also resulted in increased susceptibility to siderophore-drug antibiotics, including BAL30072, MC-1, and the FDA-approved cefiderocol (45, 46).

Quercetin displayed the strongest induction of *pirA* but also caused a growth inhibitory effect at concentrations above 10 μM. We believe that this inhibitory effect results from Fe chelation, since Fe(II) supplementation restored wild-type level growth (Fig. 3). Quercetin binds transition metals, including both Fe(II) and Fe(III), by forming complexes in 1:2 and 2:3 stoichiometry, respectively (47, 48). The Fe(II) chelating properties, which are expected to reduce the generation of ROS via the Fenton reaction, might explain the antioxidant effects frequently reported for polyphenols (43).

Chlorogenic acid, which we show here to be transported actively as an Fe(III) complex by PiuA and PirA, promoted growth of the Δ*pvdpchpiuA* mutant. One possible explanation is that both quercetin and chlorogenic acid bind Fe(III) and are transported via PiuA and PirA into the periplasm, where iron can be released from siderophores by periplasmic reductases. P. aeruginosa, for instance, takes up the xenosiderophore enterobactin via the PfeA TonB transporter (24, 36, 49, 50) and releases the bound iron using the periplasmic esterase PfeE (51). Alternatively, siderophore-Fe(III) complexes may be further translocated into the cytosol by dedicated ABC transporters. Hence, Fe(III) could be released from chlorogenic acid but not from quercetin, due to the lack of a reductase or an ABC transporter, explaining the growth retardation caused by quercetin. We believe that quercetin, luteolin, caffeic, and cholorogenic acid do, however, reach the periplasm, since they interact with the PirS sensor kinase to induce expression of *pirA*, resulting in increased susceptibility to siderophore-drug conjugates but not to other antimicrobials (Table 1; Fig. 5 and 6). Ghysels et al. (52) reported that PirA was a secondary transporter of ferric enterobactin. However, exogenous addition of enterobactin to PAO1 harboring a pfeAp-GFP or a pirAp-GFP fusion induced *pfeA* but not *pirA* expression (data not shown).

The fact that plant phenols and siderophore-drug conjugates use the same TonB transporters raises the question of competitive inhibition. This is apparently not the case, probably because of the dual, or even multiple transport systems for these compounds, as we showed previously for the siderophore-drug conjugates (33). Alternatively, plant-derived catechols could also form heterocomplexes with the siderophore-drug conjugates. Indeed, Fe(III) complexes display a six-atom coordination center; hence, two or three catechol groups are required, which can be contributed potentially by different molecules as shown previously for pyochelin and cepabactin (53).

Another unexpected finding was the transcriptional cross-talk between the inducible *pir* and the constitutively expressed *piu* loci. The *piu* locus is organized in two divergently transcribed operons (*piuA-piuB* and *piuC-piuE*), which are not genetically linked to a TCS or ECF regulatory system (Fig. 1A) (40). The *piuB* and *piuC* gene products are annotated as oxidoreductases, while the *piuE* gene encodes a hypothetical protein (pseudomonas.com). We found that expression of *piuA* is positively controlled by PirR since (i) *piuA* gene expression and PiuA protein levels were reduced in a Δ*pirR* mutant, independently of the presence of plant catechols; (ii) the Δ*pirR* mutant showed increased resistance to siderophore-drug conjugates (Table 2), and (iii) plasmid-mediated overexpression of *pirR* increased susceptibility to siderophore-drug conjugates even in the absence of PirA (Table 3). Indeed, mutations in *pirR* have been identified in both laboratory strains and clinical isolates, displaying increased cefiderocol MICs, which is consistent with our findings of reduced PiuA expression in the Δ*pirR* mutant (19-fold reduction at the protein level) (41).

PirR was recently shown to target multiple promoter sites in the genome of PAO1 (*n* = 103) and PA14 (*n* = 230), as determined by DNA-affinity-purification (DAP) sequence analysis (54). Among the genes with an inferred PirR-binding site was *piuA*, thereby confirming our data on direct regulation of *piuA* by PirR. Trouillon et al. (54) also identified PirR-binding sites in the promoter regions of *fpvB*, *pfeA*, and *optR* (PA3268), three other TonB-dependent transporter genes, as well as upstream *pvdS*, encoding an extracytosolic sigma factor. PvdS mainly regulates pyoverdine synthesis in response to iron starvation; however, we did not measure a significant difference in pyoverdine production between PA14 and the Δ*pirR* mutant under the conditions of our assays (data not shown).

The low Mg^2+^-inducible porin OprH was downregulated 2-fold in the Δ*pirR* mutant. OprH, when overexpressed, causes increased resistance to polymyxin and to gentamicin, while *oprH* deletion does not affect the antibiotic susceptibility profile of PAO1 (55). However, the susceptibilities to polymyxin and aminoglycosides were not altered in the Δ*pirR* mutant (data not shown), suggesting a minor effect of PirR on this OprH-related phenotype.

In summary, we show that plant catechols can bind Fe(III) and serve as a xenosiderophore for P. aeruginosa. Plant catechols are recognized by the PirR-PirS two-component system and are transported by the PirA and PiuA TonB-dependent transporters. To the best of our knowledge, this represents the first report of a plant-derived compound that can be used as a siderophore by bacteria. The unexpected side effect of the plant-derived catechols is the increased uptake of siderophore-drug conjugates. Hence, coadministration of these well tolerated natural catechols could further increase the efficacy of siderophore-based drug conjugates through upregulation of their dedicated TonB transporters.

## MATERIALS AND METHODS

### Bacterial strains and growth conditions.

Strains and plasmids used in this study are listed in Table S3. E. coli and P. aeruginosa were grown in lysogeny broth (LB) at 37°C with shaking (250 rpm). E. coli DH10B was use as cloning host and E. coli S17-Δpir as donor for biparental matings. Gentamicin (15 μg/mL for E. coli and 50 μg/mL for P. aeruginosa) or carbenicillin (200 μg/mL) was added for plasmid carrying strains. MICs were determined in Mueller-Hinton (MH) broth according to CLSI guidelines (56) and were repeated at least on three different occasions. M9 casamino acid medium (M9-CAA) contained 1× M9 salts, supplemented with 0.5% casamino acids (filter-sterilized) and 2 mM MgSO_4_.

10.1128/mbio.01498-22.3 TABLE S3 Bacterial strains and plasmids. List of strains and plasmids used or constructed during this study as well as their source or reference. Download Table S3, PDF file, 0.2 MB. Copyright © 2022 Luscher et al. 2022 Luscher et al. https://creativecommons.org/licenses/by/4.0/ This content is distributed under the terms of the Creative Commons Attribution 4.0 International license.

### PCR amplifications and DNA modifications.

PCR primers are listed in the supplemental material (Table S4). All primer sequences were based on the sequences from the pseudomonas.com website (57). For screening PCRs, bacterial cells were boiled at 95°C for 5 min and subsequently pelleted at 13,000 rpm for 1 min. The high-fidelity Q5 DNA polymerase (NEB) was used for PCRs. Restriction digestions were performed according to the manufacturer’s instructions at the appropriate temperature. All ligation reactions were carried out at room temperature using T4 DNA ligase (Promega). DNA preparations were performed using the GeneJET PCR purification or the GeneJET gel extraction kit (Thermo Scientific).

10.1128/mbio.01498-22.4 TABLE S4 Primers used in this study. List of primers used for the construction of defined deletion mutants as well as primers used for real-time quantitative PCR (qRT-PCR) analyses. Download Table S4, PDF file, 0.04 MB. Copyright © 2022 Luscher et al. 2022 Luscher et al. https://creativecommons.org/licenses/by/4.0/ This content is distributed under the terms of the Creative Commons Attribution 4.0 International license.

### Construction of knockout mutants.

The generation of unmarked knockout mutants was based on the protocol described by Hoang et al. (58). Briefly, DNA fragments of 500 to 700 bp were PCR-amplified using primer pairs A1/A2 and B1/B2, respectively (Table S4). For deletion of *pirR piuA*, *pirR*, and *pirS* in strain PAO1, the up- and downstream regions flanking the gene were PCR-amplified. After amplification, the obtained A and B fragments were gel-purified and ligated together with the cleaved suicide vector pEXG2. The cloned knockout fragments were verified by Sanger sequencing. The replacement vectors were mobilized into P. aeruginosa via biparental conjugation, and the generation of the unmarked mutants was carried out as previously described (59). The defined gene knockouts were verified by PCR amplification using the external primers and subsequent Sanger sequencing.

### Construction of GFP fusion plasmids.

The DNA regions upstream of the ATG start codon of the *pirA* and *piuA* coding region were amplified by PCR from genomic DNA of P. aeruginosa PAO1. The fragments harboring the putative promoter regions were cloned into the promoter probe vector pBBR1-GFP using restriction sites KpnI and BglII (60). All other constructs were prepared in a similar way using primers shown in Table S4. The Q5 high-fidelity DNA polymerase (NEB) was used for all amplifications. The PCR conditions were as follows: denaturation at 98°C for 2 min, followed by 27 cycles of 98°C for 20 s, 63°C for 30 s, 72°C for 2 min, and a final extension at 72°C for 4 min. Plasmids were transferred into P. aeruginosa by electroporation, and the cells were spread on LB-agar supplemented with carbenicillin at 200 mg/liter. All generated plasmid constructs were verified by Sanger sequencing.

### Gene expression analysis using GFP fusions.

Precultures for each strain were grown for 7 h at 37°C in LB medium, supplemented with carbenicillin at 100 μg/mL. The cultures were diluted 1,000× in saline buffer, and 5 μL of this cell suspension was added to a 96-well microtiter plate well containing 200 μL of medium supplemented with 100 μg/mL carbenicillin. Growth measured as the optical density at 600 nm (OD_600_) and fluorescence emission (excitation, 485 nm; emission, 528 nm) expressed as relative fluorescent units (RFU) were determined every 30 min in a BioTek Synergy H1 plate reader. Before each measurement, the plates were shaken for 1 min. Each strain/condition was performed two to three times independently.

### Quantitative real-time PCR.

Overnight cultures of strains grown in LB medium were diluted and inoculated into fresh M9CAA medium and grown in microtiter plates (200 μL/well) until reaching late exponential phase. RNA was extracted using the RNeasy kit (Qiagen, Germany) according to the manufacturer’s protocol. Residual genomic DNA was removed by treatment with RNase-free DNase (Promega). One μg of RNA was reverse transcribed using ImProm-II reverse transcriptase (Promega). Gene-specific primers were used for PCRs using the Rotor Gene SYBR green PCR kit (Qiagen). Quantitative PCRs were performed in a RotorGene 3000 (Corbett Research, Australia) using the following conditions: 2 min 95°C, followed by 35 cycles of 20 s at 95°C, 30 s at 60°C, and 30 s at 72°C, followed by a final extension at 72°C for 3 min. The ribosomal *rpsL* gene was used as a housekeeping reference gene (61).

### Iron uptake measurements.

The strains were grown overnight in 10 mL iron-deficient M9-CAA medium (53). The next day, the cultures were washed and adjusted to an OD_600_ of 0.1 in fresh M9-CAA medium and regrown overnight. The cultures were then washed twice in 50 mM Tris-HCl at pH 8, resuspended to an OD_600_ of 1 in 1 mL of 50 mM Tris-HCl, pH 8, and incubated for 15 min at 37°C. The complex chlorogenic acid-^55^Fe was prepared by addition of 10 mM chlorogenic acid to 50 μM ^55^FeCl_3_ (final ratio, 200:1) in 50 mM Tris-HCl, pH 8. A 10-μL aliquot of this complex was added to the bacterial suspension (500 nM ^55^Fe final concentration) and incubated for 30 min at 37°C. At time points 0 and 30 min, 100 μL of bacterial suspension was sampled in triplicate, and centrifuged. Supernatant containing the remaining complex was removed, and radioactivity of bacterial pellets was determined after 1 h of incubation in the dark in a scintillation counter. To confirm the proton gradient-dependent nature of the uptake process, the experiment was repeated in parallel with addition of 200 µM CCCP to the suspension of PA14Δ*pvdpch* cells before addition of the chlorogenic acid ^55^Fe complex.

### Proteomics analysis.

Sample preparation and MS analysis was performed as described previously (49). P. aeruginosa was grown in MH broth under standard MIC determination conditions in microtiter plates without shaking at 37°C for 18 h. The cells from three wells were combined to yield sufficient material for proteomic analyses. Three replicate cell pellets were lysed, and proteins were reduced with 5 mM Tris (2-carboxyethyl) phosphine hydrochloride and alkylated with iodoacetamide. The samples were diluted and digested with trypsin at 37°C overnight. The peptides were desalted on a C_18_ reversed-phase column and dried under vacuum. One μg of peptide was injected into a liquid chromatography-mass spectrometer (LTQ-Orbitrap Elite). The peptides were separated using an EASY nLC-1000 system (Thermo Fisher Scientific) using a C_18_ high-performance liquid chromatography column. Tandem mass spectrometry data were exported from PROGENESIS LC-MS and searched against a protein decoy database of P. aeruginosa.

### Statistical analysis.

The data were analyzed and graphs were generated using GraphPad Prism (version 9.0.1). Statistical significance values were obtained by performing two-way analysis of variance (ANOVA) with a Dunnett’s test for comparison to one control condition or Sidák’s test for timed comparisons. *P* values below 0.05 were considered statistically significant. Size effects were determined using Cohen’s *d* calculation for *t* tests, with *d* = (M_1_ – M_2_)/SD_pooled_, where *M* is the mean of two sample groups, and SD_pooled_ is the combined standard deviation of the groups defined as (_[SD12 + SD22]_/2)^1/2^ and calculated as the *q* factor in GraphPad Prism. The details of all the statistical analyses performed, including Cohen’s *d* values, are shown in a spreadsheet in the supplementary data (Table S5).

10.1128/mbio.01498-22.5 TABLE S5 Summary of statistical analyses. Summary of statistical tests used and the raw data for each of the main data sets shown in Fig. 1, 3, 4, and 5. Download Table S5, XLSX file, 0.04 MB. Copyright © 2022 Luscher et al. 2022 Luscher et al. https://creativecommons.org/licenses/by/4.0/ This content is distributed under the terms of the Creative Commons Attribution 4.0 International license.

### Data availability.

The raw data have been deposited and are available at the Zenodo data repository under https://zenodo.org/record/6670117#.YrBe8uxBxPZ.
