# Supplementary figures and images for: Plant-Derived Catechols Are Substrates of TonB-Dependent Transporters and Sensitize Pseudomonas aeruginosa to Siderophore-Drug Conjugates
Source: mBio. 2022 Jun 30;13(4):e01498-22. doi: 10.1128/mbio.01498-22 (PMC9426570; doi:10.1128/mbio.01498-22)

## pBBR-GFP

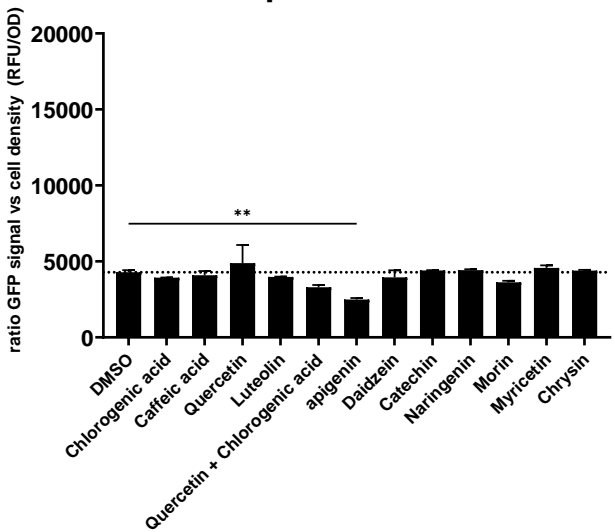

## piuAp-GFP

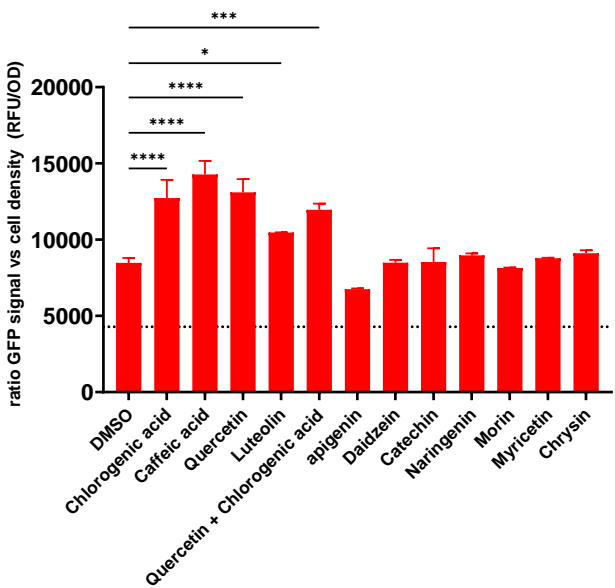

## pirAp-GFP

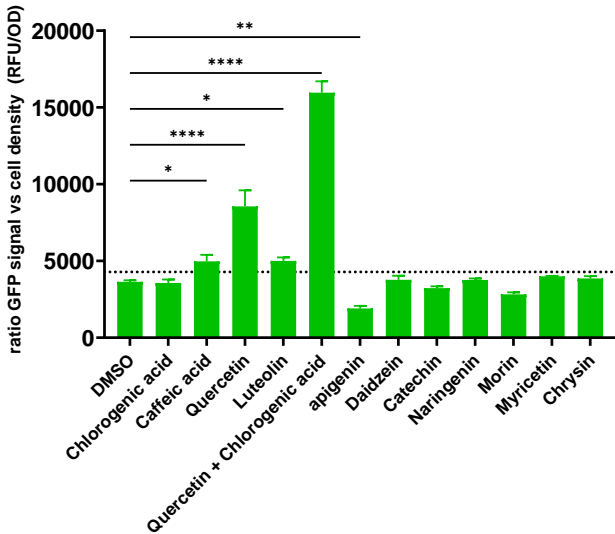

Supplement: FIG S1 [file mbio.01498-22-s0001.pdf]

# PAO1

none

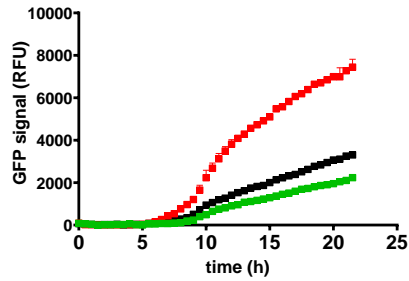

Quercetin

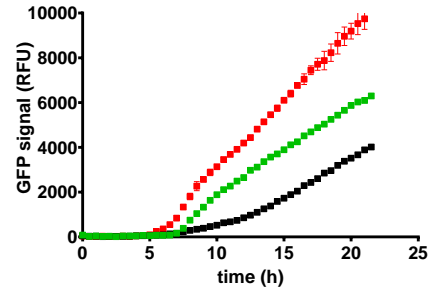

Chlorogenic acid

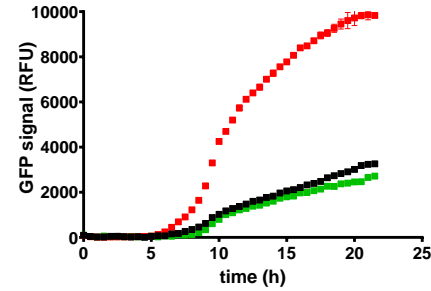

Caffeic acid

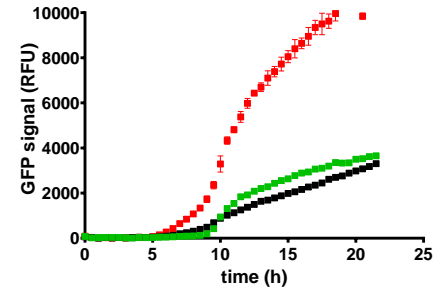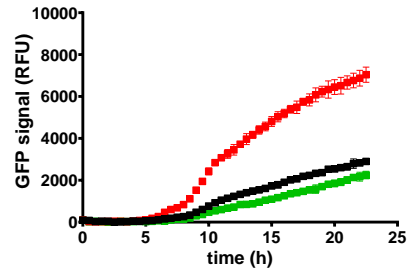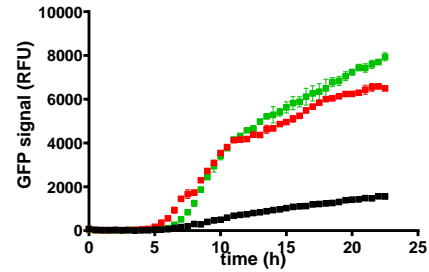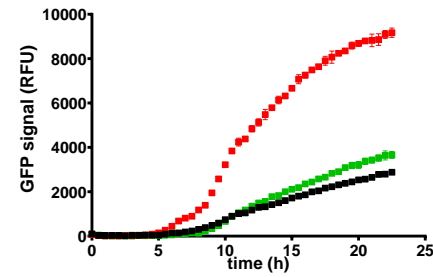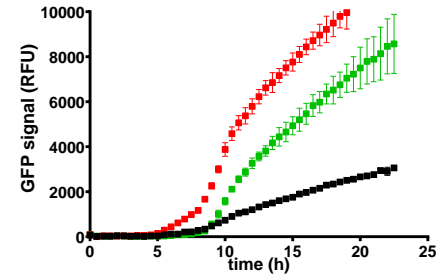

- pBBR-GFP
- piuAp-GFP
- pirAp-GFP

# PA14

Supplement: FIG S2 [file mbio.01498-22-s0002.pdf]

# PAO1

# PA14

piuA

pirA

piuA

pirA

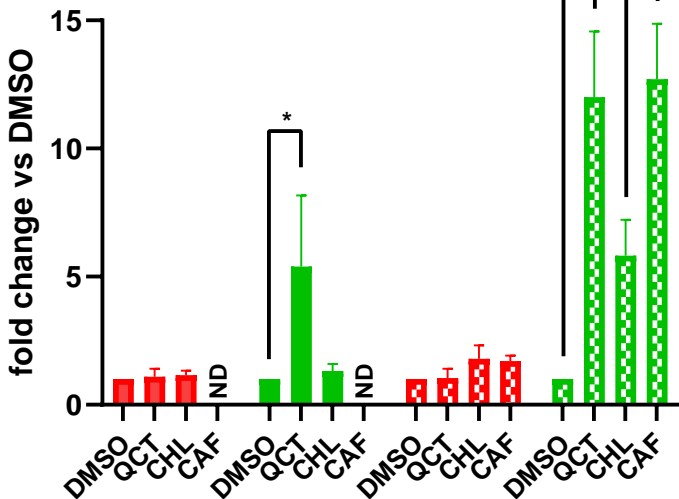

Supplement: FIG S3 [file mbio.01498-22-s0003.pdf]
