# Supplementary material for: Plant-Derived Catechols Are Substrates of TonB-Dependent Transporters and Sensitize Pseudomonas aeruginosa to Siderophore-Drug Conjugates
Source: mBio. 2022 Jun 30;13(4):e01498-22. doi: 10.1128/mbio.01498-22 (PMC9426570; doi:10.1128/mbio.01498-22)
Supplement: TABLE S1 [file mbio.01498-22-s0004.pdf]

Table S1. Effect of plant phenols on antibiotic susceptibility of PAO1

Antibiotic	MIC (μg/mL) in presence of				
	DMSO	quercetin	chlorogenic acid	caffeic acid	luteolin
BAL30072	1	**0.125**	**0.125**	0.5	**0.25**
Tetracyclin	4	4	8	8	8
Azithromycin	256	256	256	256	128
Tobramycin	0.125	0.25	0.5	0.5	0.25
Ciprofloxacin	0.06	0.125	0.125	0.125	0.125
Polymyxin B	1	1	2	1	2

Plant phenols were added at 20 μM final concentration; MICs were performed in MH-broth

MIC changes of ≥4 fold, compared to control condition (DMSO) are shown in bold
