# Supplementary material for: Plant-Derived Catechols Are Substrates of TonB-Dependent Transporters and Sensitize Pseudomonas aeruginosa to Siderophore-Drug Conjugates
Source: mBio. 2022 Jun 30;13(4):e01498-22. doi: 10.1128/mbio.01498-22 (PMC9426570; doi:10.1128/mbio.01498-22)
Supplement: TABLE S2 [file mbio.01498-22-s0005.pdf]

Table S2. Proteomic analysis of PA14 and its Δ*pirR* mutant

PA14 ORF	UniProtID	Description	FC Δ*pirR* vs PA14	Q-value*
PA14_58570	A0A0H2ZGX7	**PiuA** Putative outer membrane ferric siderophore receptor	-19,36	0,000
PA14_09730	A0A0H2ZF18	Putative dihydrodipicolinate synthase	-4,48	0,411
PA14_16110	A0A0H2ZF02	Uncharacterized protein	-2,54	0,010
PA14_26020	Q02PA2	Aminopeptidase (EC 3.4.11.1) (Leucine aminopeptidase) (PaAP)	-2,49	0,022
PA14_16670	A0A0H2ZEP1	Putative transcriptional regulator CadR	-2,48	0,378
PA14_33650	A0A0H2ZA75	Pyoverdine synthetase D	-2,43	0,042
PA14_73300	Q02DE9	ATP synthase subunit c (ATP synthase F(0) sector subunit c) (F-typ	-2,34	0,576
PA14_56810	A0A0H2ZFI0	Putative lemA-like protein	-2,28	0,086
PA14_58560	A0A0H2ZGW8	**PiuB** Probable oxidoreductase	-2,20	0,008
PA14_56670	A0A0H2ZGK5	Uncharacterized protein	-2,14	0,220
PA14_49200	A0A0H2Z863	PhoP/Q and low Mg2+ inducible outer membrane protein	-2,10	0,005
PA14_70760	A0A0H2ZIA8	Two-component sensor PhoR	-2,10	0,265
PA14_62280	Q02FW7	Hemin import ATP-binding protein HmuV (EC 3.6.3.-)	-2,03	0,030
PA14_54790	A0A0H2Z5V7	Uncharacterized protein	-1,95	NA
PA14_60710	A0A0H2ZHN0	Glutamate dehydrogenase	-1,95	NA
PA14_64090	A0A0H2ZHW1	3-dehydroquinate dehydratase (3-dehydroquinase) (EC 4.2.1.10) (	-1,92	NA
PA14_43900	A0A0H2Z8X4	Uncharacterized protein	-1,88	NA
PA14_07330	A0A0H2ZLQ2	Uncharacterized protein	-1,85	NA
PA14_67630	A0A0H2ZHL5	Putative small-conductance mechanosensitive channel	-1,84	NA
PA14_10320	A0A0H2ZG80	Putative transcriptional regulator	-1,84	NA
PA14_13780	A0A0H2ZFJ3	Putative respiratory nitrate reductase alpha subun	-1,80	NA
PA14_22800	Q02Q08	Probable intracellular septation protein A	-1,77	NA
PA14_50950	A0A0H2Z7R3	Uncharacterized protein	-1,76	NA
PA14_60760	A0A0H2ZGC9	Uncharacterized protein	-1,76	NA
PA14_33810	A0A0H2ZBF7	L-ornithine N5-oxygenase	-1,74	NA
PA14_52230	A0A0H2Z7E4	Siderophore receptor protein	-1,69	NA
PA14_33680	A0A0H2ZBG8	Ferripyoverdine receptor	-1,67	NA
PA14_48240	A0A0H2Z8J1	Putative outer membrane component of multidrug efflux pump	-1,62	NA
PA14_09500	A0A0H2ZF64	Outer membrane protein	-1,60	NA
PA14_49180	A0A0H2Z7U0	Two-component response regulator PhoP	-1,59	NA
PA14_68720	A0A0H2ZJ20	Uncharacterized protein	-1,57	NA
PA14_09520	A0A0H2ZGB3	Probable RND efflux transporter	-1,56	NA
PA14_30020	Q02NC9	NADH-quinone oxidoreductase subunit A 1 (EC 1.6.5.11) (NADH d	-1,55	NA
PA14_63150	A0A0H2ZHQ0	Two-component response regulator	-1,55	NA
PA14_52960	A0A0H2Z6Z8	Uncharacterized protein	-1,55	NA
PA14_28450	Q02NQ8	Ecotin	-1,53	NA
PA14_50340	A0A0H2Z7W6	Flagellar hook-associated protein type 3 FlgL	-1,53	NA
PA14_07000	Q02TM7	Disulfide bond formation protein B 1 (Disulfide oxidoreductase 1)	-1,53	NA
PA14_09530	A0A0H2ZGA2	RND efflux membrane fusion protein	-1,53	NA
PA14_06960	A0A0H2ZL65	Putative oxidoreductase	-1,53	NA
PA14_41880	A0A0H2Z9S9	Universal stress protein	-1,53	NA
PA14_40690	A0A0H2ZA33	Uncharacterized protein	-1,52	NA
PA14_20800	A0A0H2ZE20	Putative histidine-containing phosphotransfer (HPt) domain	-1,51	NA
PA14_23000	A0A0H2ZDQ4	Putative permease of ABC sugar transporter	-1,51	NA
PA14_69350	A0A0H2ZJ62	Uncharacterized protein	-1,50	NA
PA14_27520	A0A0H2ZBG7	Glutathione peroxidase	-1,50	NA
PA14_02530	A0A0H2ZJE3	Putative esterase	-1,49	NA
PA14_70060	A0A0H2ZI63	Putative lipoprotein	-1,49	NA
PA14_26500	A0A0H2ZCY3	Precorrin isomerase CobH	-1,48	NA
PA14_18260	A0A0H2ZEN6	Phosphofructokinase	-1,48	NA
PA14_32700	A0A0H2ZBF5	Probable transcriptional regulator	-1,48	NA
PA14_33030	A0A0H2ZBL6	L-serine dehydratase	-1,48	NA
PA14_17010	A0A0H2ZDJ0	Putative putative Na(+)/H(+) exchanger protein	-1,47	NA
PA14_44120	A0A0H2Z9C3	Putative 3-hydroxyisobutyrate dehydrogenase	-1,46	NA
PA14_43910	A0A0H2Z9D4	Uncharacterized protein	-1,46	NA
PA14_45090	A0A0H2Z8Y3	Uncharacterized protein	-1,46	NA

PA14_61140	A0A0H2ZHI5	Putative oxidoreductase, small subunit	-1,45	NA
PA14_43780	A0A0H2Z9E4	Putative oxidoreductase	-1,44	NA
PA14_53880	A0A0H2Z7D3	Uncharacterized protein	-1,44	NA
PA14_00760	A0A0H2ZJ05	Uncharacterized protein	-1,44	NA
PA14_65080	A0A0H2ZH86	UPF0313 protein PA14_65080	-1,43	NA
PA14_64960	A0A0H2ZIG0	Nicotinate phosphoribosyltransferase (NAPRTase) (EC 6.3.4.21)	-1,42	NA
PA14_29890	Q02ND8	NADH-quinone oxidoreductase subunit K (EC 1.6.5.11) (NADH deh	-1,42	NA
PA14_24230	A0A0H2ZC17	Uncharacterized protein	-1,42	NA
PA14_35290	A0A0H2ZB46	Gluconate dehydrogenase	-1,41	NA
PA14_11460	A0A0H2ZFU2	Thiamine-monophosphate kinase (TMP kinase) (Thiamine-phosph	-1,41	NA
PA14_43300	A0A0H2Z9K4	Putative esterase	-1,40	NA
PA14_72250	A0A0H2ZIN5	Putative metalloprotease	-1,39	NA
PA14_33580	A0A0H2ZA80	Uncharacterized protein	-1,39	NA
PA14_07420	A0A0H2ZKI3	Uncharacterized protein	-1,39	NA
PA14_13170	A0A0H2ZFK0	Probable metal transporting P-type ATPase	-1,39	NA
PA14_05920	A0A0H2ZLD9	Putative membrane protein	-1,38	NA
PA14_35780	A0A0H2Z9R8	Uncharacterized protein	-1,38	NA
PA14_36630	A0A0H2ZAR3	Glycogen operon protein GlgX homolog (EC 3.2.1.-)	-1,38	NA
PA14_09460	A0A0H2ZGB8	Phospho-2-dehydro-3-deoxyheptonate aldolase (EC 2.5.1.54)	-1,38	NA
PA14_26140	A0A0H2ZCS5	Putative transcriptional regulator	-1,37	NA
PA14_39130	A0A0H2ZAD9	Putative ATP-binding component of ABC transporter	-1,37	NA
PA14_67620	A0A0H2ZIK6	Uncharacterized protein	-1,37	NA
PA14_39190	Q02LA5	Undecaprenyl-diphosphatase (EC 3.6.1.27) (Bacitracin resistance p	-1,37	NA
PA14_67640	A0A0H2ZIK1	Uncharacterized protein	-1,36	NA
PA14_18010	A0A0H2ZED7	Glycerol kinase (EC 2.7.1.30) (ATP:glycerol 3-phosphotransferase)	-1,36	NA
PA14_04970	A0A0H2ZKH0	Thiamine biosynthesis protein ThiS	-1,35	NA
PA14_07810	A0A0H2ZLL6	Uncharacterized protein	-1,35	NA
PA14_40200	A0A0H2ZA35	Putative exported oxidoreductase	-1,35	NA
PA14_12140	A0A0H2ZEK2	Putative transcriptional regulator	-1,35	NA
PA14_30390	A0A0H2ZC54	Putative sulphur reductase protein	-1,35	NA
PA14_38395	A0A0H2ZLG8	Resistance-Nodulation-Cell Division (RND) multidrug efflux memb	-1,35	NA
PA14_54520	A0A0H2Z6S7	Probable porin	-1,35	NA
PA14_20290	A0A0H2ZEB1	DNA binding-protein	-1,35	NA
PA14_44860	Q02JZ7	Ureidoglycolate lyase (EC 4.3.2.3) (Ureidoglycolatase)	-1,34	NA
PA14_25510	Q02PE4	Tetraacyldisaccharide 4'-kinase (EC 2.7.1.130) (Lipid A 4'-kinase)	-1,34	NA
PA14_09760	A0A0H2ZGC0	Putative transcriptional regulator, GntR family	-1,34	NA
PA14_35720	A0A0H2ZAS4	Uncharacterized protein	-1,34	NA
PA14_34670	A0A0H2ZB95	Putative enzyme of the cupin superfamily	-1,34	NA
PA14_26750	A0A0H2ZCY7	Uncharacterized protein	-1,34	NA
PA14_62440	A0A0H2ZI11	Putative transporter	-1,34	NA
PA14_59230	A0A0H2ZGV9	Colicin immunity protein	-1,33	NA
PA14_02470	A0A0H2ZJD6	NAD(P) transhydrogenase subunit beta (EC 1.6.1.2) (Nicotinamide	-1,33	NA
PA14_48400	A0A0H2Z8I1	Uncharacterized protein	-1,33	NA
PA14_43790	A0A0H2Z991	Putative aldehyde dehydrogenase	-1,33	NA
PA14_15930	A0A0H2ZDV5	Putative hemolysin containing a CBS domain	-1,33	NA
PA14_69950	A0A0H2ZJB4	Putative acetyl-CoA hydrolase/transferase	-1,33	NA
PA14_35200	A0A0H2Z9W1	Putative acetyltransferase	-1,33	NA
PA14_06720	A0A0H2ZLJ6	Heme d1 biosynthesis protein NirF	-1,33	NA
PA14_05600	A0A0H2ZLB9	Uncharacterized protein	-1,32	NA
PA14_09540	A0A0H2ZGH2	Putative membrane protein	-1,32	NA
PA14_61220	Q02G49	Ribosomal RNA small subunit methyltransferase C (EC 2.1.1.172) (	-1,32	NA
PA14_46980	A0A0H2Z8S0	Putative two-component sensor	-1,32	NA
PA14_01440	A0A0H2ZKC7	Putative 2-hydroxychromene-2-carboxylate isomerase	-1,32	NA
PA14_34450	A0A0H2ZBA2	Putative transcriptional regulator, AraC family	-1,32	NA
PA14_36280	A0A0H2ZAX3	Putative antibiotic biosynthesis monooxygenase	-1,32	NA
PA14_49170	A0A0H2Z8C1	Two-component sensor PhoQ	-1,31	NA
PA14_40860	A0A0H2Z9S0	Putative sterol carrier protein	-1,31	NA
PA14_41420	A0A0H2Z9Y9	Uncharacterized protein	-1,31	NA
PA14_63010	A0A0H2ZI30	DNA repair protein RecN (Recombination protein N)	-1,30	NA

PA14_48860	A0A0H2Z721	Uncharacterized protein	-1,30	NA
PA14_73350	A0A0H2ZJZ9	Chromosome partitioning protein	-1,30	NA
PA14_64410	A0A0H2ZHX7	Putative Zinc-finger containing protein	-1,30	NA
PA14_26540	A0A0H2ZBN4	Uncharacterized protein	-1,29	NA
PA14_50010	A0A0H2Z7W2	Putative dehydrogenase	-1,29	NA
PA14_16480	A0A0H2ZEX9	Chemotaxis response regulator protein-glutamate methylesterase	-1,29	NA
PA14_21550	A0A0H2ZE16	Putative sigma-70 factor, ECF subfamily	-1,29	NA
PA14_34250	A0A0H2ZBC2	Putative glycerophosphoryl diester phosphodiesterase	-1,29	NA
PA14_15580	A0A0H2ZF76	Possible Type II restriction enzyme, methylase subunit	-1,29	NA
PA14_46240	A0A0H2Z8B8	Putative mechanosensitive ion channel family protein	-1,29	NA
PA14_62810	A0A0H2ZHW2	Secretion protein SecG	-1,29	NA
PA14_23840	A0A0H2ZDI2	tRNA pseudouridine synthase A (EC 5.4.99.12) (tRNA pseudouridi	-1,28	NA
PA14_52070	A0A0H2Z753	Probable transcriptional regulator	-1,28	NA
PA14_10420	Q02SV2	Tyrosine--tRNA ligase (EC 6.1.1.1) (Tyrosyl-tRNA synthetase) (TyrR	-1,28	NA
PA14_06070	A0A0H2ZLE9	Histidine kinase (EC 2.7.13.3)	-1,28	NA
PA14_57425	A0A0H2ZLI8	Penicillin-binding protein 3	-1,28	NA
PA14_07530	A0A0H2ZLR9	DNA primase (EC 2.7.7.-)	-1,28	NA
PA14_58230	A0A0H2ZGV4	Uncharacterized protein	-1,27	NA
PA14_54420	A0A0H2Z700	Anti-sigma factor MucA	-1,27	NA
PA14_72780	Q02DJ3	Pyridoxal kinase PdxY (PL kinase) (EC 2.7.1.35)	-1,27	NA
PA14_09440	A0A0H2ZG56	Phenazine biosynthesis protein PhzE	-1,27	NA
PA14_44170	A0A0H2Z8V1	Uncharacterized protein	-1,27	NA
PA14_38380	A0A0H2ZA65	Putative transcriptional regulator	-1,27	NA
PA14_71360	A0A0H2ZIE9	Uncharacterized protein	-1,26	NA
PA14_33160	Q02MN4	Uncharacterized protein PA14_33160	-1,26	NA
PA14_55640	A0A0H2ZGD2	Nuclease SbcCD subunit D	-1,26	NA
PA14_62390	A0A0H2ZI07	Putative metal-binding protein	-1,26	NA
PA14_22670	A0A0H2ZCE1	Putative ATP-binding component of ABC transporter	-1,26	NA
PA14_57110	A0A0H2ZGM3	Putative mechanosensitive ion channel family protein	-1,26	NA
PA14_29820	A0A0H2ZC16	Putative exported protein	-1,26	NA
PA14_62570	A0A0H2ZHS1	2-amino-4-hydroxy-6-hydroxymethyldihydropteridine pyrophospl	-1,26	NA
PA14_09160	A0A0H2ZG72	Bacterioferritin (EC 1.16.3.1)	-1,26	NA
PA14_62010	A0A0H2ZHS0	Putative iron ABC transporter, permease protein	-1,26	NA
PA14_08070	Q02TE1	Putative prophage major tail sheath protein	-1,26	NA
PA14_29410	A0A0H2ZCB3	Putative serine/threonine dehydratase	-1,26	NA
PA14_16680	A0A0H2ZDQ3	Uncharacterized protein	-1,26	NA
PA14_40640	A0A0H2Z9M1	Fatty acyl cis-trans isomerase	-1,26	NA
PA14_45100	A0A0H2Z7S5	Putative mucoidy inhibitor A	-1,25	NA
PA14_15670	A0A0H2ZF29	Putative multicopper oxidase	-1,25	NA
PA14_17640	Q02R79	Spermidine/putrescine import ATP-binding protein PotA (EC 3.6.3	-1,25	NA
PA14_05970	A0A0H2ZL74	Uncharacterized protein	-1,25	NA
PA14_30410	A0A0H2ZAV7	Putative 24K membrane protein	-1,25	NA
PA14_39390	A0A0H2Z907	Ribosomal protein S6 modification enzyme	-1,25	NA
PA14_23430	A0A0H2ZDM9	Putative heparinase	-1,25	NA
PA14_05650	A0A0H2ZL52	Putative exported protein	-1,25	NA
PA14_05700	A0A0H2ZKK5	Cytosine permease	-1,25	NA
PA14_58030	A0A0H2ZFR2	Fumarate hydratase class II (Fumarase C) (EC 4.2.1.2)	-1,25	NA
PA14_70350	A0A0H2ZJD7	Putative thioesterase	-1,24	NA
PA14_51060	A0A0H2Z7W5	Uncharacterized protein	-1,24	NA
PA14_56930	A0A0H2ZFJ0	Uncharacterized protein	-1,24	NA
PA14_65370	A0A0H2ZI41	N-acetylmuramoyl-L-alanine amidase	-1,24	NA
PA14_70100	A0A0H2ZJC3	Putative Glycine/D-amino acid oxidase	-1,24	NA
PA14_16210	A0A0H2ZES1	Uncharacterized protein	-1,24	NA
PA14_68080	A0A0H2ZIX7	Putative amino acid ABC transporter, permease protein	-1,24	NA
PA14_71820	A0A0H2ZII7	Putative peptidase	-1,24	NA
PA14_50240	A0A0H2Z7U8	Uncharacterized protein	-1,24	NA
PA14_61080	A0A0H2ZHG9	Putative C4-dicarboxylate-binding protein	-1,24	NA
PA14_13940	A0A0H2ZF46	Putative S-type pyocin protein	-1,24	NA
PA14_51570	A0A0H2Z7I9	Uncharacterized protein	-1,24	NA

PA14_12750	A0A0H2ZFM9	Uncharacterized protein	-1,24	NA
PA14_48700	A0A0H2Z8F7	Glutathione-regulated potassium-efflux system protein KefB	-1,24	NA
PA14_49660	A0A0H2Z6W9	Uncharacterized protein	-1,24	NA
PA14_70940	Q02DZ0	Oxygen-dependent choline dehydrogenase (CDH) (CHD) (EC 1.1.9	-1,24	NA
PA14_49340	A0A0H2Z6Y4	PcpS	-1,24	NA
PA14_26130	A0A0H2ZD25	Morphinone reductase	-1,24	NA
PA14_64370	Q02FF2	Urease subunit beta (EC 3.5.1.5) (Urea amidohydrolase subunit be	-1,24	NA
PA14_07580	Q02TI2	Glycerol-3-phosphate acyltransferase (Acyl-PO4 G3P acyltransfera	-1,24	NA
PA14_22440	A0A0H2ZDU6	Putative ATP-binding/permease fusion ABC transporter	-1,23	NA
PA14_22650	A0A0H2ZDS8	Putative ABC-type uncharacterized transport system	-1,23	NA
PA14_06010	A0A0H2ZKM0	Uncharacterized protein	-1,23	NA
PA14_41820	A0A0H2Z9L0	Para-aminobenzoate synthase component I	-1,23	NA
PA14_26280	A0A0H2ZBP7	Putative chemotaxis transducer	-1,23	NA
PA14_02220	A0A0H2ZK98	Putative chemotaxis transducer	-1,23	NA
PA14_32530	A0A0H2ZBQ1	Putative cytochrome c	-1,23	NA
PA14_13710	A0A0H2ZFE8	Uncharacterized protein	-1,23	NA
PA14_34660	A0A0H2ZA03	Transcriptional regulator GntR	-1,23	NA
PA14_61060	A0A0H2ZHI1	Putative ferredoxin--NADP+ reductase	-1,23	NA
PA14_34850	A0A0H2ZB56	Putative tRNA synthase	-1,23	NA
PA14_25090	Q02PH7	3-ketoacyl-CoA thiolase (EC 2.3.1.16) (Acetyl-CoA acyltransferase)	-1,23	NA
PA14_58130	A0A0H2ZFS3	Cell shape-determining protein MreC (Cell shape protein MreC)	-1,23	NA
PA14_02020	A0A0H2ZJY0	Putative outer membrane porin	-1,23	NA
PA14_25810	A0A0H2ZD46	Putative oxidase	-1,23	NA
PA14_16360	A0A0H2ZEY6	Putative oxidase	-1,23	NA
PA14_34970	A0A0H2ZB70	Glucose dehydrogenase	-1,23	NA
PA14_38110	Q02LI9	Serine/threonine transporter SstT (Na(+)/serine-threonine sympo	-1,23	NA
PA14_07200	A0A0H2ZLP1	Uncharacterized protein	-1,23	NA
PA14_13430	A0A0H2ZFH9	Fe(III) dicitrate transport protein FecA	-1,23	NA
PA14_15830	A0A0H2ZF13	Putative transcriptional regulator, GntR family	-1,22	NA
PA14_50740	A0A0H2Z6N8	Uncharacterized protein	-1,22	NA
PA14_44650	A0A0H2Z981	Uncharacterized protein	-1,22	NA
PA14_06060	A0A0H2ZL08	Two-component response regulator CreB	-1,22	NA
PA14_43370	Q02KC0	Potassium-transporting ATPase KdpC subunit (ATP phosphohydro	-1,22	NA
PA14_41980	A0A0H2Z9U9	Uncharacterized protein	-1,22	NA
PA14_53400	A0A0H2Z6Z9	Probable oxidoreductase	-1,22	NA
PA14_52780	A0A0H2Z712	Arginine/ornithine transport protein AotQ	-1,22	NA
PA14_60280	A0A0H2ZHL0	Type 4 fimbrial biogenesis protein FimU	-1,22	NA
PA14_31920	A0A0H2ZBS6	Putative outer membrane protein	-1,22	NA
PA14_00570	A0A0H2ZK49	Putative lipoprotein	-1,22	NA
PA14_50360	A0A0H2Z7T8	Flagellar hook-associated protein 1 FlgK	-1,22	NA
PA14_11010	A0A0H2ZFY7	Uncharacterized protein	-1,22	NA
PA14_31040	A0A0H2ZBT4	Putative cation efflux system protein	-1,22	NA
PA14_58580	Q02GS6	PKHD-type hydroxylase PA14_58580 (EC 1.14.11.-)	-1,22	NA
PA14_00690	A0A0H2ZK59	Putative metallo-beta-lactamase	-1,22	NA
PA14_44620	Q02K16	Nucleoid-associated protein PA14_44620	-1,22	NA
PA14_69440	A0A0H2ZIY8	Uroporphyrinogen-III synthase	-1,22	NA
PA14_15820	A0A0H2ZF20	N-acetylglucosamine-6-phosphate deacetylase (EC 3.5.1.25)	-1,22	NA
PA14_40700	A0A0H2Z9L4	Uncharacterized protein	-1,22	NA
PA14_00120	A0A0H2ZJS7	Putative 2-OH-lauroyltransferase	-1,21	NA
PA14_63360	A0A0H2ZGX5	Putative peroxiredoxin	-1,21	NA
PA14_43680	Q02K95	3-hydroxydecanoyl-[acyl-carrier-protein] dehydratase (EC 4.2.1.59	-1,21	NA
PA14_38210	A0A0H2ZAG1	Putative methylase	-1,21	NA
PA14_41650	A0A0H2Z9U2	Putative esterase	-1,21	NA
PA14_19530	A0A0H2ZEE3	Putative NADH-dependent FMN reductase	-1,21	NA
PA14_39300	A0A0H2Z911	Ribose operon repressor RbsR	-1,21	NA
PA14_72020	A0A0H2ZJP2	Uncharacterized protein	-1,21	NA
PA14_55670	A0A0H2ZG79	RecBCD enzyme subunit RecB (EC 3.1.11.5) (Exonuclease V subuni	-1,21	NA
PA14_12120	Q02SG4	Octanoyltransferase (EC 2.3.1.181) (Lipoate-protein ligase B) (Lipc	-1,21	NA
PA14_12630	A0A0H2ZFN8	Putative ATP-dependent helicase	-1,21	NA

PA14_45830	A0A0H2Z7N9	Putative flagellar hook-length control protein FliK	-1,21	NA
PA14_56870	A0A0H2ZGL7	Uncharacterized protein	-1,21	NA
PA14_17610	A0A0H2ZEN8	Polyamine ABC transporter protein	-1,21	NA
PA14_06540	A0A0H2ZLC0	Malonyl-[acyl-carrier protein] O-methyltransferase (Malonyl-ACP	-1,21	NA
PA14_08370	A0A0H2ZLY6	Cyclic AMP receptor-like protein	-1,21	NA
PA14_44390	A0A0H2Z9B1	Putative cytochrome c oxidase subunit	-1,21	NA
PA14_29710	A0A0H2ZC91	Uncharacterized protein	-1,21	NA
PA14_18250	A0A0H2ZEJ3	Putative phosphotransferase system, fructose-specific componen	-1,21	NA
PA14_37780	A0A0H2ZAE1	Uncharacterized protein	-1,20	NA
PA14_67990	A0A0H2ZIX4	A/G-specific adenine glycosylase	-1,20	NA
PA14_10190	A0A0H2ZG44	Putative transcriptional regulator	-1,20	NA
PA14_49930	A0A0H2Z7Y8	Uncharacterized protein	-1,20	NA
PA14_46740	A0A0H2Z7G3	Uncharacterized protein	-1,20	NA
PA14_48730	A0A0H2Z891	Putative pirin protein	-1,20	NA
PA14_57830	A0A0H2ZGR0	Uncharacterized protein	-1,20	NA
PA14_24620	A0A0H2ZD92	Uncharacterized protein	-1,20	NA
PA14_69320	A0A0H2ZHX8	Putative integral membrane transport protein	-1,20	NA
PA14_46520	A0A0H2Z7I2	Uncharacterized protein	-1,20	NA
PA14_00490	A0A0H2ZJZ8	Putative hemolysin activation/secretion protein	-1,20	NA
PA14_61050	Q02G63	Large-conductance mechanosensitive channel	-1,20	NA
PA14_47820	A0A0H2Z777	Putative transcriptional regulator	-1,20	NA
PA14_65130	A0A0H2ZIC2	Replicative DNA helicase (EC 3.6.4.12)	-1,20	NA
PA14_30660	Q02N77	UvrABC system protein C (Protein UvrC) (Excinuclease ABC subuni	-1,20	NA
PA14_58790	Q02GQ9	DNA gyrase inhibitor YacG	-1,19	NA
PA14_26760	A0A0H2ZCW6	Putative transcriptional regulator	-1,19	NA
PA14_64490	A0A0H2ZI69	Uncharacterized protein	-1,19	NA
PA14_50110	A0A0H2Z7X7	Probable flagellar assembly protein	-1,19	NA
PA14_51000	A0A0H2Z7Y6	Uncharacterized protein	-1,19	NA
PA14_52690	A0A0H2Z7B5	Arginine N-succinyltransferase (EC 2.3.1.109)	-1,19	NA
PA14_68630	A0A0H2ZJ08	Heat shock protein 15	-1,19	NA
PA14_09660	A0A0H2ZF57	Putative AMP-binding enzyme	-1,19	NA
PA14_64000	A0A0H2ZH16	Putative translation initiation factor SUI1	-1,19	NA
PA14_30070	A0A0H2ZC71	Putative secretion system protein	-1,19	NA
PA14_37580	A0A0H2ZAL8	Putative leucine-responsive regulatory protein	-1,19	NA
PA14_54510	A0A0H2Z6L5	Probable two-component response regulator	-1,19	NA
PA14_71940	A0A0H2ZJG0	ABC subunit of A-band LPS efflux transporter	-1,19	NA
PA14_62330	A0A0H2ZI03	Putative hemin degrading factor	-1,19	NA
PA14_20940	A0A0H2ZE11	Putative acyl carrier protein	-1,19	NA
PA14_67460	A0A0H2ZIJ9	Putative lipoprotein	-1,19	NA
PA14_61090	Q02G60	Ribosomal RNA large subunit methyltransferase G (EC 2.1.1.174) (	-1,19	NA
PA14_17500	Q02R92	DNA mismatch repair protein MutS	-1,19	NA
PA14_34330	A0A0H2ZB97	Putative transmembrane protein	-1,19	NA
PA14_00800	A0A0H2ZJQ0	Putative nucleic-acid-binding protein	-1,19	NA
PA14_29230	A0A0H2ZCC5	Putative hydrolase	-1,19	NA
PA14_26590	A0A0H2ZBM9	Putative transcriptional regulator, GntR family	-1,19	NA
PA14_09195	A0A0H2ZLL7	Probable major facilitator superfamily (MFS) transporter	-1,19	NA
PA14_49390	A0A0H2Z858	rRNA methyltransferase	-1,19	NA
PA14_70970	Q02DY8	HTH-type transcriptional regulator BetI	-1,18	NA
PA14_45430	A0A0H2Z8H7	Putative short-chain dehydrogenase	-1,18	NA
PA14_07570	Q02TI3	tRNA N6-adenosine threonylcarbamoyltransferase (EC 2.3.1.234)	-1,18	NA
PA14_22770	A0A0H2ZDF5	Uncharacterized protein	-1,18	NA
PA14_67790	A0A0H2ZHM2	Putative membrane-bound metallopeptidase	-1,18	NA
PA14_50310	A0A0H2Z6S6	Possible sugar nucleotidyltransferase	-1,18	NA
PA14_71800	A0A0H2ZJA7	Glucose-6-phosphate 1-dehydrogenase (G6PD) (EC 1.1.1.49)	-1,18	NA
PA14_52430	A0A0H2Z7N1	Uncharacterized protein	-1,18	NA
PA14_29930	Q02ND5	NADH-quinone oxidoreductase subunit H (EC 1.6.5.11) (NADH del	-1,18	NA
PA14_69910	A0A0H2ZJC9	ATP-dependent DNA helicase Rep (EC 3.6.4.12)	-1,18	NA
PA14_21880	A0A0H2ZCK1	Periplasmic tail-specific protease	-1,18	NA
PA14_56360	A0A0H2ZGQ6	Uncharacterized protein	-1,18	NA

PA14_12200	A0A0H2ZEJ7	DNA polymerase III, delta subunit	-1,18	NA
PA14_65390	A0A0H2ZID2	Bifunctional NAD(P)H-hydrate repair enzyme (Nicotinamide nucle	-1,18	NA
PA14_28460	A0A0H2ZCJ8	Uncharacterized protein	-1,18	NA
PA14_60070	A0A0H2ZH36	Uncharacterized protein	-1,18	NA
PA14_16830	A0A0H2ZEU0	Uncharacterized protein	-1,18	NA
PA14_65320	Q02F73	tRNA dimethylallyltransferase (EC 2.5.1.75) (Dimethylallyl diphosp	-1,18	NA
PA14_34870	A0A0H2ZAZ1	Chitinase	-1,18	NA
PA14_47760	A0A0H2Z8M4	Hydrogenobyrinate a,c-diamide synthase (EC 6.3.5.9) (Hydrogeno	-1,18	NA
PA14_54000	A0A0H2Z5Z3	Propionate catabolic protein PrpD	-1,18	NA
PA14_13740	A0A0H2ZE97	Histidine kinase (EC 2.7.13.3)	-1,18	NA
PA14_37745	A0A0H2ZLL2	Probable carbamoyl transferase	-1,18	NA
PA14_17210	Q02RB6	Acyl-[acyl-carrier-protein]--UDP-N-acetylglucosamine O-acyltransf	-1,18	NA
PA14_15810	A0A0H2ZDW3	Putative glucosamine-fructose-6-phosphate aminotransferase	-1,18	NA
PA14_02140	A0A0H2ZK93	Uncharacterized protein	-1,18	NA
PA14_72030	A0A0H2ZJP4	Uncharacterized protein	-1,18	NA
PA14_67030	A0A0H2ZHI2	Putative amino acid ABC transporter, ATP-binding protein	-1,18	NA
PA14_17450	Q02R97	5'-nucleotidase SurE (EC 3.1.3.5) (Nucleoside 5'-monophosphate p	-1,18	NA
PA14_69260	A0A0H2ZIU8	Putative enhancing lycopene biosynthesis protein 2	-1,18	NA
PA14_45410	A0A0H2Z937	Putative FlhB domain protein	-1,18	NA
PA14_17230	Q02RB4	Ribonuclease HII (RNase HII) (EC 3.1.26.4)	-1,18	NA
PA14_40880	A0A0H2Z8R0	Putative aminoglycoside phosphotransferase	-1,18	NA
PA14_21210	A0A0H2ZE41	Uncharacterized protein	-1,17	NA
PA14_58660	A0A0H2ZFW4	Transmembrane protein	-1,17	NA
PA14_63800	A0A0H2ZH03	Mg(2+) transport ATPase, P-type 2	-1,17	NA
PA14_69970	A0A0H2ZJ18	Cytochrome c5	-1,17	NA
PA14_70240	A0A0H2ZJE6	Phosphopantothenoylcysteine synthase/(R)-4'-phospho-N-pantot	-1,17	NA
PA14_44830	A0A0H2Z904	Putative polysaccharide deacetylase	-1,17	NA
PA14_47680	A0A0H2Z840	Cobinamide kinase/cobinamide phosphate guanylyltransferase	-1,17	NA
PA14_46990	A0A0H2Z870	Putative two-component response regulator	-1,17	NA
PA14_01720	A0A0H2ZK64	Alkyl hydroperoxide reductase subunit F	-1,17	NA
PA14_41570	A0A0H2Z9M5	Major porin and structural outer membrane porin OprF	-1,17	NA
PA14_30140	Q02NB9	High frequency lysogenization protein HflD homolog	-1,17	NA
PA14_07620	Q02TH9	Multifunctional CCA protein [Includes: CCA-adding enzyme (EC 2.7	-1,17	NA
PA14_08350	Q02TB6	Anthranilate phosphoribosyltransferase (EC 2.4.2.18)	-1,17	NA
PA14_20950	Q02QG7	3-oxoacyl-[acyl-carrier-protein] synthase 3 (EC 2.3.1.180) (3-oxoac	-1,17	NA
PA14_52420	Q02I76	Ribosomal protein S12 methylthiotransferase RimO (S12 MTTase)	-1,17	NA
PA14_22050	A0A0H2ZDS1	Lipid A biosynthesis lauroyltransferase (EC 2.3.1.241) (Kdo(2)-lipic	-1,17	NA
PA14_12980	A0A0H2ZFK3	Possible membrane protein	-1,17	NA
PA14_50320	A0A0H2Z829	Uncharacterized protein	-1,17	NA
PA14_41150	A0A0H2Z9X2	Putative permease of ABC transporter	-1,17	NA
PA14_08940	Q02T71	30S ribosomal protein S17	-1,17	NA
PA14_50270	A0A0H2Z834	Flagellar hook-associated protein 2 (HAP2) (Flagellar cap protein)	-1,17	NA
PA14_05580	A0A0H2ZL45	Uncharacterized protein	-1,17	NA
PA14_24390	A0A0H2ZDE8	Putative TPR domain protein	-1,17	NA
PA14_44840	A0A0H2Z7U7	Uncharacterized protein	-1,17	NA
PA14_16500	A0A0H2ZEX4	Probable two-component response regulator	-1,17	NA
PA14_00875	A0A0H2ZKT3	Serine/threonine protein kinase PpkA	-1,17	NA
PA14_39350	A0A0H2ZA91	Binding protein component of ABC ribose transporter	-1,17	NA
PA14_68940	A0A0H2ZIT7	Uncharacterized protein	-1,17	NA
PA14_64470	A0A0H2ZHY3	Uncharacterized protein	-1,17	NA
PA14_64080	A0A0H2ZH20	Thiol:disulfide interchange protein DsbD (EC 1.8.1.8) (Protein-disu	-1,16	NA
PA14_15870	A0A0H2ZF16	Putative acetyltransferase	-1,16	NA
PA14_43030	A0A0H2Z9C1	Uncharacterized protein	-1,16	NA
PA14_70480	A0A0H2ZI87	Putative endoribonuclease L-PSP	-1,16	NA
PA14_25100	A0A0H2ZCZ5	Uncharacterized protein	-1,16	NA
PA14_06130	A0A0H2ZL12	Putative streptogramin lyase	-1,16	NA
PA14_66400	A0A0H2ZIM0	Putative small-conductance mechanosensitive channel protein	-1,16	NA
PA14_49440	A0A0H2Z7S0	Probable two-component response regulator	-1,16	NA
PA14_27570	A0A0H2ZCH1	Putative GAF domain-containing protein	-1,16	NA

PA14_64450	A0A0H2ZI21	Putative heat shock protein	-1,16	NA
PA14_43630	A0A0H2Z9F3	Putative lipase	-1,16	NA
PA14_64180	A0A0H2ZHW5	tRNA-dihydrouridine synthase B (EC 1.3.1.-)	-1,16	NA
PA14_25610	A0A0H2ZD63	Maf-like protein PA14_25610	-1,16	NA
PA14_62530	A0A0H2ZHM5	Two-component sensor CbrA	-1,16	NA
PA14_47550	A0A0H2Z792	Glutathione peroxidase	-1,16	NA
PA14_71870	A0A0H2ZJB0	DNA helicase (EC 3.6.4.12)	-1,16	NA
PA14_27470	A0A0H2ZCS1	Putative zinc carboxypeptidase	-1,16	NA
PA14_32950	A0A0H2ZBL3	Uncharacterized protein	-1,16	NA
PA14_69140	A0A0H2ZJ48	Putative aromatic hydrocarbon reductase	-1,16	NA
PA14_23470	A0A0H2ZDH7	Nucleotide sugar epimerase/dehydratase WbpM	-1,16	NA
PA14_49030	A0A0H2Z8C5	Uncharacterized protein	-1,16	NA
PA14_61440	A0A0H2ZHT3	Uncharacterized protein	-1,16	NA
PA14_05000	Q02U31	tRNA (guanine-N(7)-)-methyltransferase (EC 2.1.1.33) (tRNA (guar	-1,16	NA
PA14_27500	A0A0H2ZCH6	Putative aminotransferase	-1,16	NA
PA14_55690	A0A0H2ZGL4	RecBCD enzyme subunit RecC (EC 3.1.11.5) (Exonuclease V subuni	-1,16	NA
PA14_58770	A0A0H2ZGZ1	Type 4 prepilin-like proteins leader peptide-processing enzyme (E	-1,16	NA
PA14_11370	A0A0H2ZFU7	Putative lipoprotein	-1,16	NA
PA14_67140	A0A0H2ZIG6	Putative translation initiation inhibitor	-1,16	NA
PA14_43520	A0A0H2Z9A3	Uncharacterized protein	-1,16	NA
PA14_18070	A0A0H2ZED1	Putative periplasmic metal-binding protein	-1,16	NA
PA14_20760	A0A0H2ZCU0	Chemotaxis protein methyltransferase (EC 2.1.1.80)	-1,16	NA
PA14_39610	A0A0H2ZA15	Uncharacterized protein	-1,16	NA
PA14_43400	A0A0H2Z9J6	Potassium-transporting ATPase potassium-binding subunit (ATP p	-1,16	NA
PA14_54340	A0A0H2Z705	Uncharacterized protein	-1,16	NA
PA14_16770	Q02RF2	Ribosomal RNA small subunit methyltransferase J (EC 2.1.1.242) (	-1,16	NA
PA14_20530	A0A0H2ZE39	Uncharacterized protein	-1,16	NA
PA14_48115	A0A0H2ZLV1	Alkaline protease secretion protein AprD	-1,16	NA
PA14_35790	A0A0H2ZB11	Putative homospermidine synthase	-1,15	NA
PA14_47450	Q02JD6	YcgL domain-containing protein PA14_47450	-1,15	NA
PA14_27180	A0A0H2ZCT9	Putative ErfK/YbiS/YcfS/YnhG family protein	-1,15	NA
PA14_52460	A0A0H2Z7D2	Magnesium transporter MgtE	-1,15	NA
PA14_48040	A0A0H2Z8A8	Alkaline proteinase inhibitor AprI	-1,15	NA
PA14_46450	Q02JM4	Isocitrate dehydrogenase kinase/phosphatase (IDH kinase/phospl	-1,15	NA
PA14_60050	A0A0H2ZHA5	Putative plasmid stablization protein	-1,15	NA
PA14_51690	A0A0H2Z7H9	Uncharacterized protein	-1,15	NA
PA14_15780	A0A0H2ZF60	Putative Phosphotransferase system, N-acetylglucosamine-specifi	-1,15	NA
PA14_19120	A0A0H2ZEG8	Acylhomoserine lactone dependent transcriptional regulator	-1,15	NA
PA14_21130	A0A0H2ZE45	Putative outer membrane lipoprotein	-1,15	NA
PA14_71750	A0A0H2ZJE0	Putative transcriptional regulator, LysR family	-1,15	NA
PA14_24675	A0A0H2ZLJ3	Uncharacterized protein	-1,15	NA
PA14_58100	A0A0H2ZH28	Cytoplasmic axial filament protein	-1,15	NA
PA14_11430	Q02SM0	6,7-dimethyl-8-ribityllumazine synthase (DMRL synthase) (LS) (Lur	-1,15	NA
PA14_31030	A0A0H2ZBZ8	Putative cation efflux system protein	-1,15	NA
PA14_43160	A0A0H2Z897	Putative benzoate transporter	-1,15	NA
PA14_28600	A0A0H2ZC90	Uncharacterized protein	-1,15	NA
PA14_61790	Q02G02	Peptidyl-tRNA hydrolase (PTH) (EC 3.1.1.29)	-1,15	NA
PA14_42090	A0A0H2Z8I8	Putative acyl-CoA thiolase	-1,15	NA
PA14_08400	Q02TB1	2-nonaprenyl-3-methyl-6-methoxy-1,4-benzoquinol hydroxylase (	-1,15	NA
PA14_71000	A0A0H2ZJ80	Putative lycine betaine/L-proline ABC transporter, ATP-binding su	-1,15	NA
PA14_49920	A0A0H2Z7M5	Uncharacterized protein	-1,15	NA
PA14_10480	A0A0H2ZFV3	Putative transcriptional regulator	-1,15	NA
PA14_49650	A0A0H2Z7Y1	Uncharacterized protein	-1,15	NA
PA14_23240	A0A0H2ZDD8	Putative hydrolase	-1,15	NA
PA14_72690	A0A0H2ZJU5	Putative glutamine synthetase	-1,15	NA
PA14_16000	Q02RL5	50S ribosomal protein L19	-1,15	NA
PA14_50900	Q02IK5	UPF0225 protein PA14_50900	-1,15	NA
PA14_72320	A0A0H2ZJE4	Putative spore maturation protein	-1,15	NA
PA14_62350	A0A0H2ZHU0	Putative haem/haemoglobin uptake outer membrane receptor Pł	-1,15	NA

PA14_22310	A0A0H2ZDV6	Uncharacterized protein	-1,15	NA
PA14_49290	A0A0H2Z6Y9	Uncharacterized protein	-1,15	NA
PA14_28895	A0A0H2ZL96	Uncharacterized protein	-1,15	NA
PA14_25080	Q02PH8	Fatty acid oxidation complex subunit alpha [Includes: Enoyl-CoA h	-1,15	NA
PA14_06260	A0A0H2ZLG4	Putative transcriptional regulator, LysR family	-1,15	NA
PA14_17350	A0A0H2ZEQ2	Putative redox protein	-1,15	NA
PA14_57540	A0A0H2ZGH7	Putative cytochrome c1	-1,15	NA
PA14_60200	A0A0H2ZG91	Laccase domain protein	-1,15	NA
PA14_45780	A0A0H2Z916	Flagellar protein	-1,15	NA
PA14_70830	A0A0H2ZJ76	Phosphate transport system permease protein PstA	-1,15	NA
PA14_06460	A0A0H2ZLI0	Putative hydrolase	-1,15	NA
PA14_18670	A0A0H2ZEH2	Bacterioferritin (EC 1.16.3.1)	-1,14	NA
PA14_55710	A0A0H2ZGD6	Putative lipoate-protein ligase	-1,14	NA
PA14_17250	A0A0H2ZEU8	Putative Na+/alanine symporter	-1,14	NA
PA14_16290	A0A0H2ZER6	Uncharacterized protein	-1,14	NA
PA14_09550	A0A0H2ZG47	Putative 3-carboxymuconate cyclase	-1,14	NA
PA14_09450	A0A0H2ZF69	Phenazine biosynthesis protein PhzD	-1,14	NA
PA14_12870	A0A0H2ZEF8	TesB, acyl-CoA thioesterase II	-1,14	NA
PA14_56100	A0A0H2ZFD1	Putative membrane protein	-1,14	NA
PA14_08720	Q02T91	50S ribosomal protein L11	-1,14	NA
PA14_29640	A0A0H2ZC95	Flavohemoprotein (Flavohemoglobin) (Hemoglobin-like protein) (	-1,14	NA
PA14_19640	A0A0H2ZD39	Oxidoreductase, short chain dehydrogenase/reductase	-1,14	NA
PA14_19630	A0A0H2ZE35	GTP cyclohydrolase 1 (EC 3.5.4.16) (GTP cyclohydrolase I)	-1,14	NA
PA14_51720	A0A0H2Z6C4	Protein TolB	-1,14	NA
PA14_70570	A0A0H2ZJ60	ATP-dependent DNA helicase RecG (EC 3.6.4.12)	-1,14	NA
PA14_11350	A0A0H2ZFV9	Uncharacterized protein	-1,14	NA
PA14_14650	A0A0H2ZFD4	Protein-export membrane protein SecF	-1,14	NA
PA14_62740	Q02FS9	Ribosome-binding factor A	-1,14	NA
PA14_67680	A0A0H2ZIK9	Two-component response regulator NtrC	-1,14	NA
PA14_30570	A0A0H2ZC59	Putative periplasmic spermidine/putrescine-binding protein	-1,14	NA
PA14_68740	Q02EG0	Amino-acid acetyltransferase (EC 2.3.1.1) (N-acetylglutamate synt	-1,14	NA
PA14_25110	A0A0H2ZBW1	DNA topoisomerase 1 (EC 5.99.1.2) (DNA topoisomerase I)	-1,14	NA
PA14_10540	A0A0H2ZG66	Putative iron-sulfur cluster-binding protein	-1,14	NA
PA14_27070	A0A0H2ZCU9	Putative membrane protein	-1,14	NA
PA14_50770	A0A0H2Z7S8	Probable transporter	-1,14	NA
PA14_60100	A0A0H2ZHA8	Deoxycytidine triphosphate deaminase	-1,14	NA
PA14_25710	A0A0H2ZCV9	4-amino-4-deoxychorismate lyase	-1,14	NA
PA14_71330	A0A0H2ZJK6	Putative transcriptional regulator	-1,14	NA
PA14_47330	A0A0H2Z859	Putative glutamine amidotransferase	-1,14	NA
PA14_20810	A0A0H2ZE69	ATP-dependent DNA helicase RecQ	-1,14	NA
PA14_23440	A0A0H2ZDC7	Putative group 1 glycosyl transferase	-1,14	NA
PA14_42980	A0A0H2Z8C7	Putative ClpA/B-type protease	-1,14	NA
PA14_51430	A0A0H2Z7J6	Probable coenzyme A ligase	-1,14	NA
PA14_21700	A0A0H2ZDX6	Putative sensory box histidine kinase/response regulator	-1,14	NA
PA14_62660	A0A0H2ZHV9	Uncharacterized protein	-1,14	NA
PA14_27780	A0A0H2ZCF5	Transport permease protein	-1,14	NA
PA14_13950	A0A0H2ZE83	Uncharacterized protein	-1,14	NA
PA14_30010	Q02ND0	NADH-quinone oxidoreductase subunit B (EC 1.6.5.11) (NADH deh	-1,14	NA
PA14_53900	A0A0H2Z6W4	Probable helicase	-1,14	NA
PA14_27910	A0A0H2ZCP1	Putative thioesterase	-1,14	NA
PA14_11650	A0A0H2ZEM6	Uncharacterized protein	-1,14	NA
PA14_52350	A0A0H2Z7D4	Uncharacterized protein	-1,14	NA
PA14_28720	Q02NN5	Integration host factor subunit alpha (IHF-alpha)	-1,14	NA
PA14_62000	A0A0H2ZHY9	Ferric iron-binding periplasmic protein HitA	-1,14	NA
PA14_70370	Q02E31	Orotate phosphoribosyltransferase (OPRT) (OPRTase) (EC 2.4.2.10	-1,14	NA
PA14_44400	A0A0H2Z954	Cbb3-type cytochrome c oxidase subunit	-1,14	NA
PA14_70750	A0A0H2ZJ61	Two-component response regulator PhoB	-1,14	NA
PA14_52630	Q02I61	Succinylglutamate desuccinylase (EC 3.5.1.96)	-1,14	NA
PA14_14750	A0A0H2ZE32	Iron-binding protein IscA	-1,14	NA

PA14_43250	A0A0H2Z9H6	Uncharacterized protein	-1,14	NA
PA14_48870	Q02J28	tRNA 2-thiocytidine biosynthesis protein TtcA	-1,13	NA
PA14_61850	A0A0H2ZGP0	Putative TonB-dependent receptor	-1,13	NA
PA14_52210	A0A0H2Z744	Cysteine synthase (EC 2.5.1.47)	-1,13	NA
PA14_46930	A0A0H2Z872	Putative permease of ABC transporter	-1,13	NA
PA14_51750	A0A0H2Z7H3	TolQ protein	-1,13	NA
PA14_08460	A0A0H2ZL30	Putative 2-nitropropane dioxygenase	-1,13	NA
PA14_39470	A0A0H2Z902	Uncharacterized protein	-1,13	NA
PA14_45560	A0A0H2Z8G4	Chemotaxis protein MotC	-1,13	NA
PA14_64100	A0A0H2ZIA1	Biotin carboxyl carrier protein	-1,13	NA
PA14_66570	A0A0H2ZHG4	Glutamate synthase large chain	-1,13	NA
PA14_19600	A0A0H2ZED8	Uncharacterized protein	-1,13	NA
PA14_55580	A0A0H2Z6D9	Heme oxygenase	-1,13	NA
PA14_07960	A0A0H2ZLU5	Transcriptional regulator PrtR	-1,13	NA
PA14_31870	A0A0H2ZAI2	Putative RND efflux membrane fusion protein	-1,13	NA
PA14_58540	A0A0H2ZGP1	UPF0271 protein PA14_58540	-1,13	NA
PA14_72970	A0A0H2ZJP3	Protein TonB	-1,13	NA
PA14_61580	Q02G19	Ferrochelatase (EC 4.99.1.1) (Heme synthase) (Protoheme ferro-ly	-1,13	NA
PA14_01240	A0A0H2ZKB2	Carbonic anhydrase (EC 4.2.1.1) (Carbonate dehydratase)	-1,13	NA
PA14_22660	A0A0H2ZDG1	ABC-type transporter, periplasmic component	-1,13	NA
PA14_25910	A0A0H2ZD21	Putative lipase	-1,13	NA
PA14_51670	Q02ID8	7-cyano-7-deazaguanine synthase (EC 6.3.4.20) (7-cyano-7-carbag	-1,13	NA
PA14_16860	Q02RE5	Glycerol-3-phosphate acyltransferase (GPAT) (EC 2.3.1.15)	-1,13	NA
PA14_28800	A0A0H2ZCI9	Uncharacterized protein	-1,13	NA
PA14_21860	A0A0H2ZDZ1	Uncharacterized protein	-1,13	NA
PA14_57810	Q02GZ1	UDP-N-acetylglucosamine 1-carboxyvinyltransferase (EC 2.5.1.7) (	-1,13	NA
PA14_16040	A0A0H2ZF05	Tyrosine recombinase XerD	-1,13	NA
PA14_21010	A0A0H2ZE06	Putative FAD-dependent monooxygenase	-1,13	NA
PA14_12070	A0A0H2ZFR6	Rod shape-determining protein	-1,13	NA
PA14_64140	Q02FH0	Ribosomal protein L11 methyltransferase (L11 Mtase) (EC 2.1.1.-)	-1,13	NA
PA14_68390	A0A0H2ZHQ6	Putative hydrolase	-1,13	NA
PA14_50130	A0A0H2Z7V2	Flagellar motor switch protein FliG	-1,13	NA
PA14_28820	A0A0H2ZCG3	Uncharacterized protein	-1,13	NA
PA14_70180	A0A0H2ZJ31	50S ribosomal protein L33	-1,13	NA
PA14_13110	A0A0H2ZFJ2	Probable medium-chain acyl-CoA ligase	-1,13	NA
PA14_30980	A0A0H2ZBT8	Uncharacterized protein	-1,13	NA
PA14_10530	A0A0H2ZG17	Putative transcriptional regulator, GntR family	-1,13	NA
PA14_65170	Q02F84	30S ribosomal protein S18	-1,13	NA
PA14_72920	A0A0H2ZJV0	Uncharacterized protein	-1,13	NA
PA14_69850	A0A0H2ZJC5	Putative holine-glycine betaine transporter	-1,13	NA
PA14_04010	A0A0H2ZKB4	Uncharacterized protein	-1,13	NA
PA14_38370	A0A0H2ZAH3	Uncharacterized protein	-1,13	NA
PA14_40670	A0A0H2Z8S1	Methionine synthase	-1,13	NA
PA14_14810	A0A0H2ZF73	Uncharacterized protein	-1,13	NA
PA14_61620	A0A0H2ZHF0	Putative transcriptional regulator, MerR family	-1,13	NA
PA14_27130	A0A0H2ZCU4	Transcription elongation factor GreB (Transcript cleavage factor G	-1,13	NA
PA14_07730	A0A0H2ZKJ5	Ribosomal RNA small subunit methyltransferase A (EC 2.1.1.182) (	-1,13	NA
PA14_23990	A0A0H2ZC25	General secretion pathway protein E	-1,13	NA
PA14_71450	A0A0H2ZJ88	Uncharacterized protein	-1,13	NA
PA14_50100	A0A0H2Z7L4	Flagellum-specific ATP synthase FliI	-1,13	NA
PA14_11860	A0A0H2ZFJ1	Flavin prenyltransferase UbiX (EC 2.5.1.129)	-1,13	NA
PA14_22450	A0A0H2ZDH5	Peptidyl-prolyl cis-trans isomerase (PPIase) (EC 5.2.1.8)	-1,13	NA
PA14_70390	A0A0H2ZI82	Catabolite repression control protein	-1,12	NA
PA14_26090	A0A0H2ZD27	Putative hydrolase	-1,12	NA
PA14_53840	A0A0H2Z6Q4	Uncharacterized protein	-1,12	NA
PA14_52640	A0A0H2Z722	Uncharacterized protein	-1,12	NA
PA14_17470	A0A0H2ZEP6	Putative lipoprotein NlpD	-1,12	NA
PA14_52020	A0A0H2Z7E9	Uncharacterized protein	-1,12	NA
PA14_06510	Q02TR5	8-amino-7-oxononanoate synthase (AONS) (EC 2.3.1.47) (7-keto-8	-1,12	NA

PA14_19470	A0A0H2ZEE7	Probable malate:quinone oxidoreductase (EC 1.1.5.4) (MQO) (Ma	-1,12	NA
PA14_31440	A0A0H2ZBV7	Uncharacterized protein	-1,12	NA
PA14_03940	A0A0H2ZKB1	Polyamine-transporting ATPase (EC 3.6.3.31)	-1,12	NA
PA14_50450	A0A0H2Z7W1	Flagellar hook protein FlgE	-1,12	NA
PA14_54540	A0A0H2Z6Z0	Uncharacterized protein	-1,12	NA
PA14_04810	A0A0H2ZKG0	Aldehyde dehydrogenase	-1,12	NA
PA14_05590	A0A0H2ZKY2	Methylenetetrahydrofolate reductase (EC 1.5.1.20)	-1,12	NA
PA14_34840	A0A0H2ZB73	Putative non-ribosomal peptide synthetase	-1,12	NA
PA14_12350	Q02SE8	tRNA-2-methylthio-N(6)-dimethylallyladenosine synthase (EC 2.8.	-1,12	NA
PA14_11630	A0A0H2ZFK8	Probable two-component sensor	-1,12	NA
PA14_14850	A0A0H2ZFC5	Type 4 fimbrial biogenesis protein PilF	-1,12	NA
PA14_67850	A0A0H2ZHM7	Putative ABC-type amino acid transport protein, periplasmic com	-1,12	NA
PA14_00910	A0A0H2ZK24	Uncharacterized protein	-1,12	NA
PA14_69690	Q02E84	Diaminopimelate epimerase (DAP epimerase) (EC 5.1.1.7)	-1,12	NA
PA14_14800	A0A0H2ZF82	Ferredoxin (2Fe-2S)	-1,12	NA
PA14_50890	A0A0H2Z7R9	Uncharacterized protein	-1,12	NA
PA14_02090	A0A0H2ZJY4	Putative transcriptional regulator, TetR family	-1,12	NA
PA14_41080	A0A0H2Z9X7	Putative hydroxyacylglutathione hydrolase	-1,12	NA
PA14_18700	Q02QZ9	Ribonuclease T (EC 3.1.13.-) (Exoribonuclease T) (RNase T)	-1,12	NA
PA14_26890	Q02P38	Orotidine 5'-phosphate decarboxylase (EC 4.1.1.23) (OMP decarb	-1,12	NA
PA14_72810	A0A0H2ZJV7	Putative acyl-CoA thioester hydrolase	-1,12	NA
PA14_58470	A0A0H2ZGN6	Putative dipeptide ABC transporter	-1,12	NA
PA14_28810	A0A0H2ZCH2	DNA helicase (EC 3.6.4.12)	-1,12	NA
PA14_72450	Q02DM0	Thiol:disulfide interchange protein DsbA	-1,12	NA
PA14_69340	A0A0H2ZJ63	Putative ATP-binding component of ABC transporter	-1,12	NA
PA14_40440	A0A0H2Z9V0	Putative transcriptional regulator, LysR family	-1,12	NA
PA14_73400	Q02DE1	tRNA modification GTPase MnmE (EC 3.6.-.-)	-1,12	NA
PA14_14060	A0A0H2ZFC3	Putative long-chain-fatty-acid-CoA ligase	-1,12	NA
PA14_52790	A0A0H2Z7A1	Arginine/ornithine binding protein AotJ	-1,12	NA
PA14_66940	Q02EV1	Phosphoribosyl-AMP cyclohydrolase (PRA-CH) (EC 3.5.4.19)	-1,12	NA
PA14_15200	A0A0H2ZF56	Putative TRAP-type C4-dicarboxylate transport	-1,12	NA
PA14_54270	Q02HS6	tRNA U34 carboxymethyltransferase (EC 2.5.1.-)	-1,12	NA
PA14_60920	A0A0H2ZHH3	Putative cobalamin synthesis protein/P47K family protein	-1,12	NA
PA14_70260	Q02E41	Deoxyuridine 5'-triphosphate nucleotidohydrolase (dUTPase) (EC	-1,12	NA
PA14_02060	A0A0H2ZK89	Putative outer membrane protein	-1,11	NA
PA14_43530	A0A0H2Z874	Putative glutathione S-transferase	-1,11	NA
PA14_08870	Q02T78	50S ribosomal protein L23	-1,11	NA
PA14_30330	Q02NA4	Serine--tRNA ligase (EC 6.1.1.11) (Seryl-tRNA synthetase) (SerRS) (	-1,11	NA
PA14_50550	A0A0H2Z7J0	Branched-chain amino acid transport protein BraF	-1,11	NA
PA14_41730	A0A0H2Z9W9	Uncharacterized protein	-1,11	NA
PA14_64640	A0A0H2ZHZ5	Putative transcriptional regulator, TetR family	-1,11	NA
PA14_07310	A0A0H2ZL89	Putative hydrolase	-1,11	NA
PA14_05440	A0A0H2ZL36	Uncharacterized protein	-1,11	NA
PA14_04660	A0A0H2ZK04	Putative signal transduction histidine kinase	-1,11	NA
PA14_24260	A0A0H2ZDB9	Uncharacterized protein	-1,11	NA
PA14_22620	A0A0H2ZCE6	CyaB	-1,11	NA
PA14_07260	A0A0H2ZL84	Uncharacterized protein	-1,11	NA
PA14_30430	A0A0H2ZC53	Thiosulfate sulfurtransferase	-1,11	NA
PA14_39410	A0A0H2ZAC1	Putative acetyltransferase	-1,11	NA
PA14_66560	A0A0H2ZIF1	Glutamate synthase small chain	-1,11	NA
PA14_20860	A0A0H2ZE15	Putative Tfp pilus assembly protein FimV	-1,11	NA
PA14_66270	A0A0H2ZIM8	Glutamate-ammonia-ligase adenylyltransferase (EC 2.7.7.42) (Glu	-1,11	NA
PA14_71580	A0A0H2ZJK9	Uncharacterized protein	-1,11	NA
PA14_58410	A0A0H2ZH48	Putative outer membrane porin	-1,11	NA
PA14_16530	Q02RH2	Lysine--tRNA ligase (EC 6.1.1.6) (Lysyl-tRNA synthetase) (LysRS)	-1,11	NA
PA14_07030	A0A0H2ZLN0	Putative cytochrome c	-1,11	NA
PA14_66710	Q02EW8	50S ribosomal protein L31	-1,11	NA
PA14_58270	A0A0H2ZGU6	Uncharacterized protein	-1,11	NA
PA14_49480	A0A0H2Z6X8	Uncharacterized protein	-1,11	NA

PA14_25560	A0A0H2ZCW7	Ribonuclease E (RNase E) (EC 3.1.26.12)	-1,11	NA
PA14_69990	A0A0H2ZJ23	Alanine racemase (EC 5.1.1.1)	-1,11	NA
PA14_53510	A0A0H2Z7G0	Uncharacterized protein	-1,11	NA
PA14_27950	A0A0H2ZCN9	Putative anti-anti-sigma factor	-1,11	NA
PA14_28650	Q02NP1	Threonine--tRNA ligase (EC 6.1.1.3) (Threonyl-tRNA synthetase) (T	-1,11	NA
PA14_11510	Q02SL4	GTP cyclohydrolase-2 (EC 3.5.4.25) (GTP cyclohydrolase II)	-1,11	NA
PA14_17510	A0A0H2ZDG7	Putative TolB-like translocation protein	-1,11	NA
PA14_41400	Q02KT3	UDP-2,3-diacylglucosamine hydrolase (EC 3.6.1.54) (UDP-2,3-diac	-1,11	NA
PA14_72700	A0A0H2ZJG5	Putative N-formylglutamate amidohydrolase	-1,11	NA
PA14_59770	A0A0H2ZHG6	Putative two component response regulator	-1,11	NA
PA14_44670	Q02K11	Cell division protein ZipA	-1,11	NA
PA14_25020	A0A0H2ZD00	Putative ABC transporter, ATP-binding protein	-1,11	NA
PA14_08380	A0A0H2ZL25	Putative redox protein	-1,11	NA
PA14_53500	A0A0H2Z614	Uncharacterized protein	-1,11	NA
PA14_27490	A0A0H2ZCS2	Uncharacterized protein	-1,11	NA
PA14_55960	A0A0H2ZGG1	Chemotactic transducer PctC	-1,11	NA
PA14_71030	A0A0H2ZJH0	Putative glycine betaine/L-proline ABC transporter, periplasmic cc	-1,11	NA
PA14_00050	A0A0H2ZJS1	DNA gyrase subunit B (EC 5.99.1.3)	-1,11	NA
PA14_56680	A0A0H2ZFH1	Ferrous iron transport protein B	-1,11	NA
PA14_35540	A0A0H2Z9T9	Transcriptional regulator BkdR	-1,11	NA
PA14_09600	A0A0H2ZG97	D-alanine--D-alanine ligase (EC 6.3.2.4) (D-Ala-D-Ala ligase) (D-ala	-1,11	NA
PA14_02130	Q02UQ8	Uncharacterized protein PA14_02130	-1,11	NA
PA14_30650	A0A0H2ZC55	Response regulator GacA	-1,11	NA
PA14_23010	A0A0H2ZDE6	Putative ATP-binding component of ABC transporter	-1,11	NA
PA14_46890	A0A0H2Z8P8	Putative short-chain dehydrogenase	-1,11	NA
PA14_30310	Q02NA6	Outer-membrane lipoprotein carrier protein	-1,11	NA
PA14_03270	A0A0H2ZKN9	Uncharacterized protein	-1,11	NA
PA14_70450	Q02E25	DNA-directed RNA polymerase subunit omega (RNAP omega subu	-1,11	NA
PA14_62650	A0A0H2ZI19	Uncharacterized protein	-1,11	NA
PA14_05070	A0A0H2ZL16	Putative methionine biosynthesis protein	-1,11	NA
PA14_53040	A0A0H2Z780	Uncharacterized protein	-1,11	NA
PA14_46070	A0A0H2Z8R5	Guanidinobutyrase	-1,11	NA
PA14_32610	A0A0H2ZBN7	Thiol:disulfide interchange protein DsbG	-1,11	NA
PA14_17900	A0A0H2ZEN1	Transcriptional regulator MetR	-1,11	NA
PA14_59130	A0A0H2ZGU9	Uncharacterized protein	-1,11	NA
PA14_06040	A0A0H2ZK97	Uncharacterized protein	-1,11	NA
PA14_04640	A0A0H2ZL43	Putative SAM-dependent methyltransferase	-1,11	NA
PA14_08340	A0A0H2ZLF8	Anthranilate synthase component II	-1,11	NA
PA14_68610	Q02EH1	33 kDa chaperonin (Heat shock protein 33 homolog) (HSP33)	-1,11	NA
PA14_70730	Q02E04	4-hydroxybenzoate octaprenyltransferase (EC 2.5.1.-) (4-HB polyp	-1,11	NA
PA14_43720	A0A0H2Z9E9	Putative transcriptional regulator	-1,11	NA
PA14_16640	A0A0H2ZEW4	Putative lipoprotein	-1,11	NA
PA14_62690	A0A0H2ZHN3	Uncharacterized protein	-1,10	NA
PA14_37290	A0A0H2ZAI8	Putative allophanate hydrolase subunit 1	-1,10	NA
PA14_17220	Q02RB5	Lipid-A-disaccharide synthase (EC 2.4.1.182)	-1,10	NA
PA14_23450	A0A0H2ZC92	Putative NAD dependent epimerase/dehydratase	-1,10	NA
PA14_28200	A0A0H2ZCL4	Uncharacterized protein	-1,10	NA
PA14_11520	A0A0H2ZFU9	Uncharacterized protein	-1,10	NA
PA14_23200	A0A0H2ZDJ2	Putative oxidoreductase, short-chain dehydrogenase/reductase fa	-1,10	NA
PA14_61980	A0A0H2ZGP9	Uncharacterized protein	-1,10	NA
PA14_24580	A0A0H2ZD95	Uncharacterized protein	-1,10	NA
PA14_57690	A0A0H2ZFN5	Uncharacterized protein	-1,10	NA
PA14_52570	Q02I65	Carbon storage regulator homolog	-1,10	NA
PA14_50660	A0A0H2Z7I1	Putative cysteine synthase	-1,10	NA
PA14_41710	A0A0H2Z8L8	Putative membrain protein	-1,10	NA
PA14_04110	A0A0H2ZKC4	D-3-phosphoglycerate dehydrogenase	-1,10	NA
PA14_05460	A0A0H2ZLA8	Adenosylmethionine-8-amino-7-oxononanoate aminotransferase	-1,10	NA
PA14_23650	A0A0H2ZDB3	Putative short-chain dehydrogenase	-1,10	NA
PA14_58630	A0A0H2ZGX3	Putative ornithine decarboxylase	-1,10	NA

PA14_25580	A0A0H2ZBU3	Pseudouridine synthase (EC 5.4.99.-)	-1,10	NA
PA14_05250	A0A0H2ZL93	Dihydroorotase (DHOase) (EC 3.5.2.3)	-1,10	NA
PA14_59845	A0A0H2ZM14	Uncharacterized protein	-1,10	NA
PA14_58490	A0A0H2ZH52	Putative dipeptide transport system ATP-binding pr	-1,10	NA
PA14_48830	A0A0H2Z7W9	Putative transcriptional regulator	-1,10	NA
PA14_53150	A0A0H2Z7I4	Probable ATP-binding/permease fusion ABC transporter	-1,10	NA
PA14_22690	A0A0H2ZDM2	Trk system potassium uptake protein	-1,10	NA
PA14_64170	A0A0H2ZH24	Uncharacterized protein	-1,10	NA
PA14_66660	A0A0H2ZID6	Type 4 fimbrial biogenesis protein PilM	-1,10	NA
PA14_31840	A0A0H2ZBT1	Putative protease	-1,10	NA
PA14_57650	A0A0H2ZGZ2	Cell division protein ZapE (Z ring-associated protein ZapE)	-1,10	NA
PA14_06570	Q02TR2	ATP-dependent dethiobiotin synthetase BioD (EC 6.3.3.3) (DTB sy	-1,10	NA
PA14_68955	A0A0H2ZL71	Probable FAD-dependent oxidoreductase	-1,10	NA
PA14_60490	A0A0H2ZHL9	Putative cytochrome c	-1,10	NA
PA14_45800	A0A0H2Z8Z5	Flagellar motor switch protein FliM	-1,10	NA
PA14_66110	A0A0H2ZII4	Putative glycosyl transferase	-1,10	NA
PA14_43770	A0A0H2Z8Y2	Putative transcriptional regulator	-1,10	NA
PA14_25230	A0A0H2ZD58	Transcription-repair-coupling factor (TRCF) (EC 3.6.4.-)	-1,10	NA
PA14_44420	A0A0H2Z7Z9	Putative ferredoxin	-1,10	NA
PA14_50250	A0A0H2Z6T1	Flagellar protein FliS	-1,10	NA
PA14_73190	A0A0H2ZJK1	GlmR transcriptional regulator	-1,10	NA
PA14_21640	A0A0H2ZDY1	Putative oxidoreductase, short chain dehydrogenase/reductase	-1,10	NA
PA14_15680	A0A0H2ZF22	tRNA-specific adenosine deaminase (EC 3.5.4.33)	-1,10	NA
PA14_01970	A0A0H2ZJ93	Putative RND efflux transporter	-1,10	NA
PA14_16660	A0A0H2ZF06	Putative metal-transporting P-type ATPase	-1,10	NA
PA14_07090	Q02TL9	S-adenosylmethionine synthase (AdoMet synthase) (EC 2.5.1.6) (M	-1,10	NA
PA14_60270	A0A0H2ZH51	Putative Glycine/D-amino acid oxidases	-1,10	NA
PA14_10560	A0A0H2ZEV4	Uncharacterized protein	-1,10	NA
PA14_27940	A0A0H2ZCR0	Putative two-component response regulator	-1,10	NA
PA14_67720	Q02EN8	Protein-export protein SecB	-1,10	NA
PA14_58070	A0A0H2ZGU5	Putative tldD protein	-1,10	NA
PA14_11380	Q02SM4	Transcriptional repressor NrdR	-1,10	NA
PA14_08695	A0A0H2ZLU7	Protein translocase subunit SecE	-1,10	NA
PA14_27230	A0A0H2ZBI6	Putative transcriptional regulator, MarR family	-1,10	NA
PA14_70430	A0A0H2ZJF5	Putative alpha helix protein	-1,10	NA
PA14_17370	A0A0H2ZEU6	Putative transport permease protein	-1,10	NA
PA14_64360	A0A0H2ZI59	Putative phosphinothricin N-acetyltransferase	-1,10	NA
PA14_27770	A0A0H2ZCQ1	Putative ABC transporter, ATP-binding protein	-1,10	NA
PA14_39960	Q02L47	Phenazine biosynthesis protein PhzB 2	-1,10	NA
PA14_65640	A0A0H2ZI57	Uncharacterized protein	-1,10	NA
PA14_38080	A0A0H2ZAC4	Putative cysteine proteases	-1,10	NA
PA14_24445	A0A0H2ZM32	NAD-dependent glutamate dehydrogenase	-1,10	NA
PA14_44500	Q02K26	Adenine phosphoribosyltransferase (APRT) (EC 2.4.2.7)	-1,10	NA
PA14_44060	A0A0H2Z8W2	Succinate dehydrogenase, cytochrome b556 subunit	-1,10	NA
PA14_54410	A0A0H2Z6T8	Negative regulator for alginate biosynthesis MucB	-1,10	NA
PA14_14770	Q02RW5	Co-chaperone protein HscB homolog	-1,10	NA
PA14_25620	A0A0H2ZCW3	Uncharacterized protein	-1,10	NA
PA14_48760	A0A0H2Z8F2	Putative hydrolase	-1,10	NA
PA14_03360	A0A0H2ZK79	Uncharacterized protein	-1,10	NA
PA14_66790	Q02EW3	ATP-dependent protease ATPase subunit HslU (Unfoldase HslU)	-1,10	NA
PA14_19610	A0A0H2ZEB7	Uncharacterized protein	-1,10	NA
PA14_00710	A0A0H2ZJ00	Osmotically inducible protein OsmC	-1,10	NA
PA14_05360	A0A0H2ZKI7	Type IV pili methyl-accepting chemotaxis transducer PilJ	-1,10	NA
PA14_38570	A0A0H2Z951	Putative sigma-54 dependent transcriptional regulator	-1,10	NA
PA14_70620	A0A0H2ZI97	Putative rubredoxin reductase	-1,10	NA
PA14_06450	A0A0H2ZL34	Putative acyl-CoA carboxylase subunit	-1,10	NA
PA14_25450	A0A0H2ZD48	Putative lipoprotein releasing system, permease protein	-1,10	NA
PA14_41050	A0A0H2ZA09	DNA polymerase III, epsilon chain	-1,10	NA
PA14_35300	A0A0H2ZB37	Uncharacterized protein	-1,10	NA

PA14_36250	A0A0H2ZAU1	Uncharacterized protein	-1,10	NA
PA14_68210	A0A0H2ZIY2	dTDP-4-dehydrorhamnose 3,5-epimerase	-1,10	NA
PA14_14140	A0A0H2ZFB8	Uncharacterized protein	-1,10	NA
PA14_24770	A0A0H2ZBY5	Uncharacterized protein	-1,10	NA
PA14_35840	Q02M20	Uncharacterized protein PA14_35840	-1,10	NA
PA14_68060	A0A0H2ZHN5	Putative amino acid ABC transporter, ATP-binding protein	-1,10	NA
PA14_62720	Q02FT1	30S ribosomal protein S15	-1,10	NA
PA14_60460	Q02GA9	50S ribosomal protein L21	-1,10	NA
PA14_44000	A0A0H2Z8W8	Dihydrolipoyllysine-residue succinyltransferase component of 2-o	-1,10	NA
PA14_29575	A0A0H2ZM39	Uncharacterized protein	-1,09	NA
PA14_54370	Q02HR9	Elongation factor 4 (EF-4) (EC 3.6.5.n1) (Ribosomal back-transloca	-1,09	NA
PA14_07280	A0A0H2ZKU8	Putative periplasmic protein	-1,09	NA
PA14_20200	A0A0H2ZE88	Nitrous-oxide reductase (EC 1.7.2.4) (N(2)OR) (N2O reductase)	-1,09	NA
PA14_62300	A0A0H2ZHL6	Putative periplasmic binding protein	-1,09	NA
PA14_08480	Q02TA4	N-acetyl-gamma-glutamyl-phosphate reductase (AGPR) (EC 1.2.1.	-1,09	NA
PA14_25760	A0A0H2ZD49	DNA polymerase III, delta prime subunit	-1,09	NA
PA14_51780	Q02IC9	Holliday junction ATP-dependent DNA helicase RuvB (EC 3.6.4.12)	-1,09	NA
PA14_24700	A0A0H2ZBZ0	Uncharacterized protein	-1,09	NA
PA14_14690	A0A0H2ZF87	tRNA (cytidine/uridine-2'-O-)-methyltransferase TrmJ (EC 2.1.1.20	-1,09	NA
PA14_13190	A0A0H2ZFI7	Uncharacterized protein	-1,09	NA
PA14_62850	A0A0H2ZHT1	Dihydropteroate synthase (DHPS) (EC 2.5.1.15) (Dihydropteroate	-1,09	NA
PA14_41450	A0A0H2Z9N6	Uncharacterized protein	-1,09	NA
PA14_14830	Q02RW0	Dual-specificity RNA methyltransferase RlmN (EC 2.1.1.192) (23S r	-1,09	NA
PA14_03720	A0A0H2ZJT3	Putative sensory box/GGDEF domain/EAL domain protein	-1,09	NA
PA14_68810	A0A0H2ZJ25	Putative adenylate cyclase	-1,09	NA
PA14_15790	A0A0H2ZEU5	Putative phosphoenolpyruvate-protein phosphotransferase	-1,09	NA
PA14_65690	A0A0H2ZI99	3',5'-cyclic adenosine monophosphate phosphodiesterase CpdA (3	-1,09	NA
PA14_24690	A0A0H2ZD24	Putative D-alanyl-D-alanine carboxypeptidase	-1,09	NA
PA14_04670	Q02U58	Formamidopyrimidine-DNA glycosylase (Fapy-DNA glycosylase) (E	-1,09	NA
PA14_54620	A0A0H2Z5W5	Probable aldehyde dehydrogenase	-1,09	NA
PA14_31850	A0A0H2ZBL7	Putative protease	-1,09	NA
PA14_49380	Q02IY2	Succinyl-diaminopimelate desuccinylase (SDAP desuccinylase) (EC	-1,09	NA
PA14_66920	Q02EV2	Probable protein kinase UbiB (EC 2.7.-.-) (Ubiquinone biosynthesis	-1,09	NA
PA14_69640	A0A0H2ZJ01	Protein CyaY	-1,09	NA
PA14_69000	A0A0H2ZJ38	Aminopeptidase P	-1,09	NA
PA14_67750	A0A0H2ZIW4	Putative rhodanese-like domain protein	-1,09	NA
PA14_65350	Q02F72	DNA mismatch repair protein MutL	-1,09	NA
PA14_41590	A0A0H2Z8M2	Putative cytoplasmic membrane protein	-1,09	NA
PA14_23280	A0A0H2ZDL6	Prephenate dehydratase	-1,09	NA
PA14_43190	A0A0H2Z9I1	Probable oxidoreductase	-1,09	NA
PA14_57880	A0A0H2ZH07	Putative toluene tolerance ABC efflux transporter	-1,09	NA
PA14_09420	A0A0H2ZGI5	Probable phenazine biosynthesis protein	-1,09	NA
PA14_04390	Q02U79	RNA pyrophosphohydrolase (EC 3.6.1.-) ((Di)nucleoside polyphosp	-1,09	NA
PA14_12100	A0A0H2ZFH3	D-ala-D-ala-carboxypeptidase	-1,09	NA
PA14_15720	Q02RN8	Membrane-bound lytic murein transglycosylase F (EC 4.2.2.n1) (M	-1,09	NA
PA14_56700	A0A0H2ZGS8	Uncharacterized protein	-1,09	NA
PA14_30830	A0A0H2ZAT0	Putative two-component response regulator	-1,09	NA
PA14_31500	A0A0H2ZBV3	Putative acyl-coenzyme A synthetases/AMP-(Fatty) acid ligases	-1,09	NA
PA14_17960	A0A0H2ZEM7	Glycerol kinase (EC 2.7.1.30) (ATP:glycerol 3-phosphotransferase)	-1,09	NA
PA14_49470	A0A0H2Z7Z1	Ribonucleoside-diphosphate reductase subunit beta (EC 1.17.4.1)	-1,09	NA
PA14_71620	A0A0H2ZJC8	N5-carboxyaminoimidazole ribonucleotide mutase (N5-CAIR muta	-1,09	NA
PA14_11340	A0A0H2ZEP3	Putative thioredoxin	-1,09	NA
PA14_18710	Q02QZ8	Dihydroorotase (DHOase) (EC 3.5.2.3)	-1,09	NA
PA14_73220	Q02DF6	Bifunctional protein GlmU [Includes: UDP-N-acetylglucosamine py	-1,09	NA
PA14_49410	A0A0H2Z6Y0	Probable cold-shock protein	-1,09	NA
PA14_03350	A0A0H2ZKW0	Uncharacterized protein	-1,09	NA
PA14_08930	Q02T72	50S ribosomal protein L29	-1,09	NA
PA14_73250	Q02DF3	ATP synthase gamma chain (ATP synthase F1 sector gamma subur	-1,09	NA
PA14_51220	A0A0H2Z7M4	Probable glycosyl transferase	-1,09	NA

PA14_67010	A0A0H2ZIR1	Putative methyl-accepting chemotaxis transducer	-1,09	NA
PA14_14370	A0A0H2ZFF4	Putative ABC-transporter ATP-binding component	-1,09	NA
PA14_41680	Q02KR0	Putative phosphoenolpyruvate synthase regulatory protein (PEP s	-1,09	NA
PA14_69220	A0A0H2ZJ54	Exopolyphosphatase	-1,09	NA
PA14_70850	A0A0H2ZJH9	Phosphate ABC transporter, permease protein	-1,09	NA
PA14_67920	A0A0H2ZIX2	Imidazole glycerol phosphate synthase subunit HisH (EC 2.4.2.-) (I	-1,09	NA
PA14_01390	A0A0H2ZJU0	Putative Sco1/SenC family protein	-1,09	NA
PA14_71930	A0A0H2ZIJ7	Glycosyltransferase WbpX	-1,09	NA
PA14_15070	A0A0H2ZEY4	Outer membrane copper receptor OprC	-1,09	NA
PA14_15770	A0A0H2ZF17	Uncharacterized protein	-1,09	NA
PA14_05190	A0A0H2ZL88	Twitching motility protein PilU	-1,09	NA
PA14_31300	A0A0H2ZBY6	Uncharacterized protein	-1,09	NA
PA14_57600	A0A0H2ZGQ8	Putative oxidoreductase, aldo/keto reductase famil	-1,09	NA
PA14_04910	A0A0H2ZL67	Cell division ATP-binding protein FtsE	-1,09	NA
PA14_12160	A0A0H2ZFU8	Putative murein transglycosylase	-1,09	NA
PA14_06150	A0A0H2ZLF5	Uncharacterized protein	-1,09	NA
PA14_13600	A0A0H2ZFK6	Putative binding protein component of ABC transpor	-1,09	NA
PA14_09410	A0A0H2ZGB1	Probable pyrodoxamine 5'-phosphate oxidase	-1,09	NA
PA14_41900	A0A0H2Z9K5	2-dehydropantoate 2-reductase (EC 1.1.1.169) (Ketopantoate red	-1,09	NA
PA14_08860	Q02T79	50S ribosomal protein L4	-1,09	NA
PA14_60530	Q02GA3	UPF0114 protein PA14_60530	-1,09	NA
PA14_02840	A0A0H2ZKE7	Beta-ketoadipate enol-lactone hydrolase	-1,09	NA
PA14_26350	A0A0H2ZBP2	Putative metallo-beta-lactamase family protein	-1,09	NA
PA14_41190	A0A0H2ZA00	Peptidylprolyl isomerase (EC 5.2.1.8)	-1,09	NA
PA14_30800	A0A0H2ZC47	Putative nitroreductase	-1,09	NA
PA14_63070	A0A0H2ZHP6	Putative transcriptional regulator, GntR family	-1,09	NA
PA14_49460	A0A0H2Z855	Ribonucleoside-diphosphate reductase (EC 1.17.4.1)	-1,09	NA
PA14_68380	A0A0H2ZIP8	Putative DP compound hydrolase	-1,09	NA
PA14_37710	A0A0H2Z9A2	Elongation factor G (EF-G)	-1,09	NA
PA14_01520	A0A0H2ZJU8	Putative transcriptional regulator	-1,09	NA
PA14_15610	A0A0H2ZF34	Uncharacterized protein	-1,09	NA
PA14_49740	A0A0H2Z892	Uncharacterized protein	-1,09	NA
PA14_05060	A0A0H2ZK38	Uncharacterized protein	-1,09	NA
PA14_18870	A0A0H2ZEH7	Uncharacterized protein	-1,09	NA
PA14_45840	A0A0H2Z910	Uncharacterized protein	-1,09	NA
PA14_47800	A0A0H2Z8H8	Putative tonB-dependent receptor	-1,09	NA
PA14_63100	A0A0H2ZHX5	Putative ferredoxin	-1,09	NA
PA14_09040	Q02T61	50S ribosomal protein L15	-1,09	NA
PA14_42740	Q02KH3	Methylthioribulose-1-phosphate dehydratase (MTRu-1-P dehydra	-1,09	NA
PA14_04980	Q02U32	Thiazole synthase (EC 2.8.1.10)	-1,09	NA
PA14_64840	A0A0H2ZH69	Putative oxidoreductase	-1,09	NA
PA14_67040	A0A0H2ZIF9	Putative amino acid ABC transporter, permease protein	-1,09	NA
PA14_11400	A0A0H2ZFZ9	Riboflavin biosynthesis protein RibD	-1,09	NA
PA14_25320	A0A0H2ZCY5	Na(+)-translocating NADH-quinone reductase subunit C (Na(+)-NC	-1,09	NA
PA14_67470	A0A0H2ZHK8	Uncharacterized protein	-1,09	NA
PA14_54220	A0A0H2Z7B6	Inhibitor of cysteine peptidase	-1,09	NA
PA14_54450	A0A0H2Z7B0	L-aspartate oxidase (EC 1.4.3.16)	-1,09	NA
PA14_37610	A0A0H2ZAC8	Kynureninase (EC 3.7.1.3) (L-kynurenine hydrolase)	-1,09	NA
PA14_24665	A0A0H2ZLV8	Uncharacterized protein	-1,08	NA
PA14_66950	Q02EV0	Phosphoribosyl-ATP pyrophosphatase (PRA-PH) (EC 3.6.1.31)	-1,08	NA
PA14_19940	A0A0H2ZEE0	Uncharacterized protein	-1,08	NA
PA14_27480	Q02NZ3	Protease HtpX (EC 3.4.24.-) (Heat shock protein HtpX)	-1,08	NA
PA14_69820	A0A0H2ZI47	Uncharacterized protein	-1,08	NA
PA14_57330	Q02H29	UDP-N-acetylmuramate--L-alanine ligase (EC 6.3.2.8) (UDP-N-acet	-1,08	NA
PA14_08880	Q02T77	50S ribosomal protein L2	-1,08	NA
PA14_68710	A0A0H2ZJ16	Putative transcriptional accessory protein	-1,08	NA
PA14_11960	A0A0H2ZFR0	Uncharacterized protein	-1,08	NA
PA14_66960	Q02EU9	Sec-independent protein translocase protein TatA	-1,08	NA
PA14_63370	A0A0H2ZHR9	Putative glycine cleavage system H protein	-1,08	NA

PA14_52720	A0A0H2Z7L6	Acetylornithine aminotransferase (ACOAT) (EC 2.6.1.11)	-1,08	NA
PA14_52740	A0A0H2Z7A8	Transcriptional regulator ArgR	-1,08	NA
PA14_25990	A0A0H2ZCT5	Putative mMagnesium chelatase, subunit ChlI	-1,08	NA
PA14_45280	A0A0H2Z8I7	Cytochrome c-type biogenesis protein	-1,08	NA
PA14_62840	Q02FS3	Phosphoglucosamine mutase (EC 5.4.2.10)	-1,08	NA
PA14_60330	Q02GC0	4-hydroxy-3-methylbut-2-enyl diphosphate reductase (EC 1.17.7.4	-1,08	NA
PA14_24290	A0A0H2ZD55	Glycine betaine transmethylase	-1,08	NA
PA14_21230	A0A0H2ZCQ4	RNA polymerase-associated protein RapA (EC 3.6.4.-) (ATP-depen	-1,08	NA
PA14_26960	A0A0H2ZBK5	Putative acyl-CoA thioesterase	-1,08	NA
PA14_45810	A0A0H2Z8T2	Putative flagellar protein FliL	-1,08	NA
PA14_01940	A0A0H2ZKH1	Putative RND efflux membrane fusion protein	-1,08	NA
PA14_22550	A0A0H2ZDG9	Putative LysR-family transcriptional regulator	-1,08	NA
PA14_03950	A0A0H2ZJU9	Polyamine transport protein PotH	-1,08	NA
PA14_65500	Q02F61	Phosphatidylserine decarboxylase proenzyme (EC 4.1.1.65) [Cleav	-1,08	NA
PA14_53290	A0A0H2Z624	Thioredoxin reductase (EC 1.8.1.9)	-1,08	NA
PA14_59220	A0A0H2ZG14	Pyocin S5	-1,08	NA
PA14_06190	A0A0H2ZL17	Putative glutathione S-transferase	-1,08	NA
PA14_45380	A0A0H2Z8W4	Cytochrome c biogenesis ATP-binding export protein CcmA (EC 3.(	-1,08	NA
PA14_71440	A0A0H2ZJK0	L-threonine aldolase (EC 4.1.2.48)	-1,08	NA
PA14_57760	A0A0H2ZGS0	AlgW protein	-1,08	NA
PA14_70490	A0A0H2ZJ55	Putative lipoprotein	-1,08	NA
PA14_08850	Q02T80	50S ribosomal protein L3	-1,08	NA
PA14_18060	A0A0H2ZEQ1	Putative cytochrome B561	-1,08	NA
PA14_52750	A0A0H2Z7B3	Arginine/ornithine transport protein AotP	-1,08	NA
PA14_69150	Q02EC7	3-octaprenyl-4-hydroxybenzoate carboxy-lyase (EC 4.1.1.98) (Poly	-1,08	NA
PA14_50860	A0A0H2Z6N0	Uncharacterized protein	-1,08	NA
PA14_17140	A0A0H2ZDI4	Zinc metalloprotease (EC 3.4.24.-)	-1,08	NA
PA14_43690	Q02K94	3-oxoacyl-[acyl-carrier-protein] synthase 1 (EC 2.3.1.41) (3-oxoacy	-1,08	NA
PA14_18650	A0A0H2ZD96	Glutaredoxin	-1,08	NA
PA14_21340	A0A0H2ZDP4	Long-chain-fatty-acid--CoA ligase	-1,08	NA
PA14_65710	A0A0H2ZI61	Adenosine diphosphate sugar pyrophosphatase	-1,08	NA
PA14_65960	A0A0H2ZIK5	3-deoxy-D-manno-octulosonic-acid (KDO) transferase	-1,08	NA
PA14_52140	A0A0H2Z7E8	Uncharacterized protein	-1,08	NA
PA14_23980	A0A0H2ZD74	Secretion protein XcpP	-1,08	NA
PA14_58750	A0A0H2ZH73	Type 4 fimbrial biogenesis protein PilB	-1,08	NA
PA14_40630	Q02KZ2	Fe/S biogenesis protein NfuA	-1,08	NA
PA14_60410	A0A0H2ZHD0	Uncharacterized protein	-1,08	NA
PA14_63580	A0A0H2ZHT2	Nitrate-inducible formate dehydrogenase, beta subunit	-1,08	NA
PA14_45520	A0A0H2Z7P8	Putative plasmid partitioning protein	-1,08	NA
PA14_25880	A0A0H2ZD42	Electron transfer flavoprotein alpha-subunit	-1,08	NA
PA14_11550	Q02SL1	1-deoxy-D-xylulose-5-phosphate synthase (EC 2.2.1.7) (1-deoxyxy	-1,08	NA
PA14_66000	A0A0H2ZHD2	Putative oxidoreductase	-1,08	NA
PA14_07870	A0A0H2ZLM1	Putative binding protein component of ABC transporter	-1,08	NA
PA14_44050	A0A0H2Z9F0	Succinate dehydrogenase hydrophobic membrane anchor subuni	-1,08	NA
PA14_00060	A0A0H2ZJX4	Putative acyltransferase	-1,08	NA
PA14_29800	A0A0H2ZC84	Putative chemotaxis transducer	-1,08	NA
PA14_73410	Q02DE0	Membrane protein insertase YidC (Foldase YidC) (Membrane inte	-1,08	NA
PA14_62960	Q02FR2	Chaperone protein DnaJ	-1,08	NA
PA14_51740	A0A0H2Z770	TolR protein	-1,08	NA
PA14_41060	Q02KV8	Ribonuclease H (RNase H) (EC 3.1.26.4)	-1,08	NA
PA14_61960	A0A0H2ZHP0	Uncharacterized protein	-1,08	NA
PA14_65000	A0A0H2ZI65	Azurin	-1,08	NA
PA14_65590	A0A0H2ZI94	Uncharacterized protein	-1,08	NA
PA14_29980	A0A0H2ZC94	NADH dehydrogenase I chain E	-1,08	NA
PA14_69770	A0A0H2ZJC1	Uncharacterized protein	-1,08	NA
PA14_08750	Q02T88	50S ribosomal protein L7/L12	-1,08	NA
PA14_23090	A0A0H2ZCB4	2-keto-3-deoxy-6-phosphogluconate aldolase	-1,08	NA
PA14_60580	A0A0H2ZHE0	Uncharacterized protein	-1,08	NA
PA14_57260	Q02H34	UDP-3-O-acyl-N-acetylglucosamine deacetylase (UDP-3-O-acyl-Glc	-1,08	NA

PA14_12110	Q02SG5	UPF0250 protein PA14_12110	-1,08	NA
PA14_67580	Q02EP8	tRNA sulfurtransferase (EC 2.8.1.4) (Sulfur carrier protein ThiS sulf	-1,08	NA
PA14_14730	Q02RW8	Cysteine desulfurase IscS (EC 2.8.1.7)	-1,08	NA
PA14_51810	Q02IC6	Probable transcriptional regulatory protein PA14_51810	-1,08	NA
PA14_64390	A0A0H2ZI17	Urease subunit alpha (EC 3.5.1.5) (Urea amidohydrolase subunit a	-1,08	NA
PA14_52080	A0A0H2Z7F2	Uncharacterized protein	-1,08	NA
PA14_30180	A0A0H2ZC63	Monomeric isocitrate dehydrogenase	-1,08	NA
PA14_70950	Q02DY9	NAD/NADP-dependent betaine aldehyde dehydrogenase (BADH)	-1,08	NA
PA14_52280	A0A0H2Z7E0	Uncharacterized protein	-1,08	NA
PA14_63990	A0A0H2ZHZ8	Biosynthetic arginine decarboxylase (ADC) (EC 4.1.1.19)	-1,08	NA
PA14_60400	Q02GB4	30S ribosomal protein S20	-1,08	NA
PA14_45480	A0A0H2Z8G9	Uncharacterized protein	-1,08	NA
PA14_27210	Q02P14	Elongation factor P (EF-P)	-1,08	NA
PA14_17060	Q02RC8	30S ribosomal protein S2	-1,08	NA
PA14_62930	A0A0H2ZHP2	Carbamoyl-phosphate synthase small chain (EC 6.3.5.5) (Carbamo	-1,08	NA
PA14_25500	A0A0H2ZD64	Putative biopolymer transport protein	-1,08	NA
PA14_67540	A0A0H2ZHL1	Uncharacterized protein	-1,08	NA
PA14_16020	A0A0H2ZET5	Uncharacterized protein	-1,08	NA
PA14_73120	A0A0H2ZJJ5	Putative periplasmic transport protein	-1,08	NA
PA14_23320	Q02PW9	Cytidylate kinase (CK) (EC 2.7.4.25) (Cytidine monophosphate kina	-1,08	NA
PA14_57780	A0A0H2ZGJ2	Histidinol dehydrogenase (HDH) (EC 1.1.1.23)	-1,08	NA
PA14_71840	A0A0H2ZJN1	Uncharacterized protein	-1,08	NA
PA14_25660	A0A0H2ZD54	3-oxoacyl-[acyl-carrier-protein] reductase	-1,08	NA
PA14_14600	Q02RX7	Queuine tRNA-ribosyltransferase (EC 2.4.2.29) (Guanine insertion	-1,08	NA
PA14_36530	A0A0H2Z9L3	Uncharacterized protein	-1,08	NA
PA14_61770	A0A0H2ZHV4	Ribose-phosphate pyrophosphokinase (RPPK) (EC 2.7.6.1) (5-phos	-1,08	NA
PA14_62200	A0A0H2ZHP9	Penicillin-binding protein 1B (PBP-1b) (PBP1b) (Murein polymeras	-1,08	NA
PA14_61300	A0A0H2ZGH1	Putative methyl-accepting chemotaxis protein (MCP)	-1,08	NA
PA14_47350	A0A0H2Z8H1	Uncharacterized protein	-1,08	NA
PA14_61740	Q02G06	Outer-membrane lipoprotein LolB	-1,08	NA
PA14_50140	A0A0H2Z6T6	Flagellar M-ring protein	-1,08	NA
PA14_52260	A0A0H2Z7P1	Histidine kinase (EC 2.7.13.3)	-1,08	NA
PA14_73240	Q02DF4	ATP synthase subunit beta (EC 3.6.3.14) (ATP synthase F1 sector s	-1,08	NA
PA14_21000	A0A0H2ZE28	Uncharacterized protein	-1,08	NA
PA14_05840	A0A0H2ZL63	Glutaryl-CoA dehydrogenase	-1,08	NA
PA14_42180	A0A0H2Z9B0	Uncharacterized protein	-1,08	NA
PA14_11280	A0A0H2ZEP8	Putative lipoprotein	-1,08	NA
PA14_40390	A0A0H2Z8U0	Molybdate-binding periplasmic protein modA	-1,08	NA
PA14_04520	A0A0H2ZJZ2	Uncharacterized protein	-1,08	NA
PA14_54350	A0A0H2Z5X4	Signal peptidase I (EC 3.4.21.89)	-1,08	NA
PA14_60260	A0A0H2ZG94	Two-component response regulator PilR	-1,08	NA
PA14_64460	A0A0H2ZH44	Putative phosphate starvation-inducible protein	-1,08	NA
PA14_50300	A0A0H2Z7U3	Uncharacterized protein	-1,08	NA
PA14_25650	A0A0H2ZBT9	Malonyl CoA-acyl carrier protein transacylase (EC 2.3.1.39)	-1,08	NA
PA14_64110	A0A0H2ZI43	Biotin carboxylase	-1,08	NA
PA14_14390	A0A0H2ZE55	Putative ABC-type transport protein, periplasmic c	-1,08	NA
PA14_71960	A0A0H2ZJN8	Transport permease protein	-1,07	NA
PA14_03090	A0A0H2ZKI6	Putative CBS domain	-1,07	NA
PA14_49510	A0A0H2Z7R5	Immunity protein S3I structureal protein	-1,07	NA
PA14_44280	Q02K46	Ribosomal RNA large subunit methyltransferase M (EC 2.1.1.186)	-1,07	NA
PA14_60550	A0A0H2ZH67	Putative ATP-dependent protease	-1,07	NA
PA14_08560	A0A0H2ZKQ1	Tyrosine--tRNA ligase (EC 6.1.1.1) (Tyrosyl-tRNA synthetase)	-1,07	NA
PA14_72930	A0A0H2ZJW8	Uncharacterized protein	-1,07	NA
PA14_14220	A0A0H2ZFB3	Putative nucleoid-associated protein	-1,07	NA
PA14_57290	A0A0H2ZGN1	Cell division protein FtsA	-1,07	NA
PA14_43950	Q02K73	Succinate--CoA ligase [ADP-forming] subunit beta (EC 6.2.1.5) (Suc	-1,07	NA
PA14_11130	A0A0H2ZFW6	Putative short chain dehydrogenase	-1,07	NA
PA14_45400	A0A0H2Z7Q5	Uncharacterized protein	-1,07	NA
PA14_08910	Q02T74	30S ribosomal protein S3	-1,07	NA

PA14_62710	Q02FT2	Polyribonucleotide nucleotidyltransferase (EC 2.7.7.8) (Polynuclec	-1,07	NA
PA14_70200	A0A0H2ZI72	Putative binding protein component of ABC dipeptide transporter	-1,07	NA
PA14_57130	Q02H45	UPF0234 protein PA14_57130	-1,07	NA
PA14_08900	Q02T75	50S ribosomal protein L22	-1,07	NA
PA14_40070	A0A0H2Z9Y0	Putative glutathione S-transferase	-1,07	NA
PA14_36310	A0A0H2ZAN8	Hydrogen cyanide synthase HcnC	-1,07	NA
PA14_51820	Q02IC5	Aspartate--tRNA(Asp/Asn) ligase (EC 6.1.1.23) (Aspartyl-tRNA synt	-1,07	NA
PA14_17180	Q02RB8	UDP-3-O-acylglucosamine N-acyltransferase (EC 2.3.1.-)	-1,07	NA
PA14_03163	A0A0H2ZLJ8	Uncharacterized protein	-1,07	NA
PA14_25160	Q02PH1	LexA repressor (EC 3.4.21.88)	-1,07	NA
PA14_63410	A0A0H2ZHW7	Uncharacterized protein	-1,07	NA
PA14_52660	Q02I59	N-succinylarginine dihydrolase (EC 3.5.3.23)	-1,07	NA
PA14_60800	A0A0H2ZHF4	Putative ABC transporter, ATP-binding protein	-1,07	NA
PA14_07130	A0A0H2ZKT8	Transketolase (EC 2.2.1.1)	-1,07	NA
PA14_04410	A0A0H2ZKE1	Phosphoenolpyruvate-protein phosphotransferase	-1,07	NA
PA14_08450	A0A0H2ZLZ4	Putative membrane protein	-1,07	NA
PA14_05300	A0A0H2ZKI4	Putative tonB domain protein	-1,07	NA
PA14_03810	Q02UC5	Agmatine deiminase (EC 3.5.3.12) (Agmatine iminohydrolase)	-1,07	NA
PA14_19310	A0A0H2ZEF7	Putative transmembrane protein	-1,07	NA
PA14_14680	A0A0H2ZE36	Extragenic suppressor protein SuhB	-1,07	NA
PA14_09180	A0A0H2ZF88	UvrABC system protein A (UvrA protein) (Excinuclease ABC subun	-1,07	NA
PA14_63030	A0A0H2ZHU3	Outer membrane protein assembly factor BamE	-1,07	NA
PA14_28340	A0A0H2ZCM5	Putative peroxidase	-1,07	NA
PA14_09200	A0A0H2ZGD4	Single-stranded DNA-binding protein (SSB)	-1,07	NA
PA14_16560	A0A0H2ZDR1	Putative lipoprotein	-1,07	NA
PA14_24080	A0A0H2ZDC9	Type II secretion system protein L (T2SS protein L)	-1,07	NA
PA14_24330	A0A0H2ZDB4	Putative export protein	-1,07	NA
PA14_43940	A0A0H2Z828	Succinate--CoA ligase [ADP-forming] subunit alpha (EC 6.2.1.5)	-1,07	NA
PA14_19050	Q02QX1	Methionine--tRNA ligase (EC 6.1.1.10) (Methionyl-tRNA synthetas	-1,07	NA
PA14_17030	A0A0H2ZES5	Succinyldiaminopimelate transaminase	-1,07	NA
PA14_41690	A0A0H2Z9L9	Uncharacterized protein	-1,07	NA
PA14_25530	Q02PE2	3-deoxy-manno-octulosonate cytidylyltransferase (EC 2.7.7.38) (C	-1,07	NA
PA14_62420	A0A0H2ZGS5	Putative zinc metallopeptidase	-1,07	NA
PA14_21990	Q02Q78	Probable M18 family aminopeptidase 2 (EC 3.4.11.-)	-1,07	NA
PA14_11970	Q02SH7	Putative 3-methyladenine DNA glycosylase (EC 3.2.2.-)	-1,07	NA
PA14_12310	Q02SF0	Endoribonuclease YbeY (EC 3.1.-.-)	-1,07	NA
PA14_12490	A0A0H2ZFN6	AMP nucleosidase (EC 3.2.2.4)	-1,07	NA
PA14_48740	A0A0H2Z841	Putative oxidoreductase	-1,07	NA
PA14_11560	A0A0H2ZFY9	Geranyltranstransferase	-1,07	NA
PA14_64440	Q02FE6	Oxaloacetate decarboxylase (EC 4.1.1.3)	-1,07	NA
PA14_62780	Q02FS6	Ribosome maturation factor RimP	-1,07	NA
PA14_14940	A0A0H2ZF68	Putative aminotransferase	-1,07	NA
PA14_29850	A0A0H2ZC81	NADH-quinone oxidoreductase subunit N (EC 1.6.5.11) (NADH del	-1,07	NA
PA14_68980	A0A0H2ZIS9	2-octaprenyl-6-methoxyphenyl hydroxylase	-1,07	NA
PA14_08840	Q02T81	30S ribosomal protein S10	-1,07	NA
PA14_11680	A0A0H2ZFX6	Probable two-component regulator	-1,07	NA
PA14_66080	A0A0H2ZHD7	Lipid A export ATP-binding/permease protein MsbA (EC 3.6.3.-)	-1,07	NA
PA14_61190	A0A0H2ZHR0	Putative hemolysin activation/secretion protein	-1,07	NA
PA14_06990	A0A0H2ZKG2	Putative exported protein	-1,07	NA
PA14_28680	Q02NN8	50S ribosomal protein L20	-1,07	NA
PA14_68200	A0A0H2ZIM7	Glucose-1-phosphate thymidylyltransferase (EC 2.7.7.24)	-1,07	NA
PA14_12740	A0A0H2ZEG6	Uncharacterized protein	-1,07	NA
PA14_50710	A0A0H2Z7H6	Probable NADH dehydrogenase	-1,07	NA
PA14_17340	Q02RA5	2-C-methyl-D-erythritol 4-phosphate cytidylyltransferase (EC 2.7.7	-1,07	NA
PA14_25360	A0A0H2ZBV4	FAD:protein FMN transferase (EC 2.7.1.180)	-1,07	NA
PA14_57890	A0A0H2ZGR5	Arabinose 5-phosphate isomerase (API) (EC 5.3.1.13)	-1,07	NA
PA14_15030	A0A0H2ZF63	2-isopropylmalate synthase (EC 2.3.3.13) (Alpha-IPM synthase) (A	-1,07	NA
PA14_14630	A0A0H2ZF84	Protein translocase subunit SecD	-1,07	NA
PA14_43850	A0A0H2Z9D9	Chaperone protein HtpG (Heat shock protein HtpG) (High tempera	-1,07	NA

PA14_04100	A0A0H2ZL15	Uncharacterized protein	-1,07	NA
PA14_69670	A0A0H2ZJ02	Diaminopimelate decarboxylase (DAP decarboxylase) (DAPDC) (E(	-1,07	NA
PA14_48500	A0A0H2Z878	Putative transcriptional regulator	-1,07	NA
PA14_66220	A0A0H2ZIM2	Lipopolysaccharide core heptose(I) kinase (EC 2.7.1.-)	-1,07	NA
PA14_54320	Q02HS3	GTPase Era	-1,07	NA
PA14_18750	A0A0H2ZEI6	Lactoylglutathione lyase (EC 4.4.1.5) (Glyoxalase I)	-1,07	NA
PA14_08980	Q02T67	30S ribosomal protein S14	-1,07	NA
PA14_00990	A0A0H2ZK28	Uncharacterized protein	-1,07	NA
PA14_46960	A0A0H2Z8J7	Gamma-glutamyltranspeptidase	-1,07	NA
PA14_22460	A0A0H2ZCG0	Putative hydrolase, alpha/beta fold family	-1,07	NA
PA14_03960	A0A0H2ZKT6	Polyamine transport protein PotI	-1,07	NA
PA14_58330	A0A0H2ZH42	Uncharacterized protein	-1,07	NA
PA14_32940	A0A0H2ZBM1	Probable transcriptional regulator	-1,07	NA
PA14_07740	A0A0H2ZLL0	4-hydroxythreonine-4-phosphate dehydrogenase (EC 1.1.1.262) (4	-1,07	NA
PA14_58250	A0A0H2ZGM4	Putative membrane protein	-1,07	NA
PA14_22680	A0A0H2ZDP7	Putative permease of ABC transporter	-1,07	NA
PA14_42720	Q02KH5	Enolase-phosphatase E1 (EC 3.1.3.77) (2,3-diketo-5-methylthio-1-	-1,07	NA
PA14_10550	A0A0H2ZFU6	Putative sulfite or nitrite reductas	-1,07	NA
PA14_28710	A0A0H2ZC85	Phenylalanine--tRNA ligase beta subunit (EC 6.1.1.20) (Phenylalan	-1,07	NA
PA14_09080	Q02T58	30S ribosomal protein S13	-1,07	NA
PA14_58440	A0A0H2ZGW7	Putative dipeptide ABC transport system permease	-1,07	NA
PA14_65480	A0A0H2ZID7	Sulfurtransferase	-1,07	NA
PA14_00210	A0A0H2ZJL6	Putative lysin domain	-1,07	NA
PA14_14130	A0A0H2ZFD0	Uncharacterized protein	-1,06	NA
PA14_62400	A0A0H2ZHU5	Putative aminotransferase	-1,06	NA
PA14_05180	A0A0H2ZKV1	Twitching motility protein PilT	-1,06	NA
PA14_09470	A0A0H2ZGA6	Probable phenazine biosynthesis protein	-1,06	NA
PA14_08760	Q02T87	DNA-directed RNA polymerase subunit beta (RNAP subunit beta)	-1,06	NA
PA14_08810	Q02T84	30S ribosomal protein S7	-1,06	NA
PA14_40950	Q02KW6	NADH pyrophosphatase (EC 3.6.1.22)	-1,06	NA
PA14_10220	A0A0H2ZFX5	Putative SH3 domain protein	-1,06	NA
PA14_38820	Q02LD3	Coenzyme PQQ synthesis protein B (Pyrroloquinoline quinone bio	-1,06	NA
PA14_25960	A0A0H2ZD37	Putative cobalamin biosynthesis protein cobW	-1,06	NA
PA14_36330	A0A0H2ZAW9	Hydrogen cyanide synthase HcnA	-1,06	NA
PA14_21400	A0A0H2ZDX4	Putative ATP-dependent helicase	-1,06	NA
PA14_65605	A0A0H2ZL55	DNA topoisomerase 4 subunit A (EC 5.99.1.3) (Topoisomerase IV s	-1,06	NA
PA14_60350	A0A0H2ZH57	Peptidyl-prolyl cis-trans isomerase (EC 5.2.1.8)	-1,06	NA
PA14_10670	A0A0H2ZEU7	Aminoglycoside 3'-phosphotransferase type IIb	-1,06	NA
PA14_46470	Q02JM2	Erythronate-4-phosphate dehydrogenase (EC 1.1.1.290)	-1,06	NA
PA14_59550	A0A0H2ZHE4	Uncharacterized protein	-1,06	NA
PA14_15340	Q02RS2	GMP synthase [glutamine-hydrolyzing] (EC 6.3.5.2) (GMP syntheta	-1,06	NA
PA14_07020	A0A0H2ZL70	Putative membrane protein	-1,06	NA
PA14_25490	A0A0H2ZBU7	Putative tolQ-type transport protein	-1,06	NA
PA14_64200	Q02FG6	Bifunctional purine biosynthesis protein PurH [Includes: Phosphor	-1,06	NA
PA14_26530	A0A0H2ZCP7	Precorrin-3 methylase CobJ	-1,06	NA
PA14_66160	A0A0H2ZIL6	Putative glycosyl transferase	-1,06	NA
PA14_25740	Q02PC5	Thymidylate kinase (EC 2.7.4.9) (dTMP kinase)	-1,06	NA
PA14_56240	A0A0H2ZGA7	Pyruvate kinase (EC 2.7.1.40)	-1,06	NA
PA14_73260	Q02DF2	ATP synthase subunit alpha (EC 3.6.3.14) (ATP synthase F1 sector	-1,06	NA
PA14_34700	A0A0H2ZB08	Putative beta lactamase	-1,06	NA
PA14_27160	A0A0H2ZBJ1	Acyl-CoA thioesterase I	-1,06	NA
PA14_28660	A0A0H2ZCH3	Translation initiation factor IF-3	-1,06	NA
PA14_28830	A0A0H2ZC75	Uncharacterized protein	-1,06	NA
PA14_41130	A0A0H2ZA05	Putative binding protein component of ABC transporter	-1,06	NA
PA14_62730	Q02FT0	tRNA pseudouridine synthase B (EC 5.4.99.25) (tRNA pseudouridin	-1,06	NA
PA14_66120	A0A0H2ZIC4	Uncharacterized protein	-1,06	NA
PA14_25195	Q02PG9	Beta-hexosaminidase (EC 3.2.1.52) (Beta-N-acetylhexosaminidase	-1,06	NA
PA14_30290	A0A0H2ZC79	Cell division/stress response protein	-1,06	NA
PA14_25180	A0A0H2ZCY8	Transcriptional regulator PsrA	-1,06	NA

PA14_05260	Q02U10	Aspartate carbamoyltransferase (EC 2.1.3.2) (Aspartate transcarb	-1,06	NA
PA14_19290	A0A0H2ZD66	Putative ATP-dependent RNA helicase	-1,06	NA
PA14_65300	A0A0H2ZH96	GTPase HflX (GTP-binding protein HflX)	-1,06	NA
PA14_70270	Q02E40	Phosphomannomutase/phosphoglucomutase (PMM / PGM) (EC 5	-1,06	NA
PA14_64220	A0A0H2ZI48	Phosphoribosylamine--glycine ligase (EC 6.3.4.13) (GARS) (Glycina	-1,06	NA
PA14_12780	A0A0H2ZFD3	Probable two-component response regulator	-1,06	NA
PA14_12900	A0A0H2ZFK7	Histone-like protein HU form N	-1,06	NA
PA14_49870	A0A0H2Z7N0	Peptide deformylase (PDF) (EC 3.5.1.88) (Polypeptide deformylase	-1,06	NA
PA14_08710	A0A0H2ZF93	Transcription termination/antitermination protein NusG	-1,06	NA
PA14_07890	A0A0H2ZLC2	Putative permease of ABC transporter	-1,06	NA
PA14_16320	A0A0H2ZEY5	Peptidyl-prolyl cis-trans isomerase (EC 5.2.1.8)	-1,06	NA
PA14_70470	A0A0H2ZJ42	Guanosine-3',5'-bis(Diphosphate) 3'-pyrophosphohydrolase	-1,06	NA
PA14_59970	A0A0H2ZH26	Uncharacterized protein	-1,06	NA
PA14_15460	A0A0H2ZF37	Mercuric reductase (EC 1.16.1.1) (Hg(II) reductase)	-1,06	NA
PA14_60500	A0A0H2ZHE1	Peptidyl-prolyl cis-trans isomerase (EC 5.2.1.8)	-1,06	NA
PA14_69060	A0A0H2ZJ44	Putative permease of ABC transporter	-1,06	NA
PA14_17100	Q02RC5	Ribosome-recycling factor (RRF) (Ribosome-releasing factor)	-1,06	NA
PA14_67050	A0A0H2ZIR8	Putative amino acid ABC transporter, periplasmic amino acid-binc	-1,06	NA
PA14_25770	A0A0H2ZD30	Type 4 fimbrial biogenesis protein PilZ	-1,06	NA
PA14_01490	A0A0H2ZK55	Putative hemolysin	-1,06	NA
PA14_05990	A0A0H2ZL03	Putative drug resistance transporter, EmrB/QacA family	-1,06	NA
PA14_69810	A0A0H2ZJ10	Nitrogen regulatory protein PII	-1,06	NA
PA14_41950	A0A0H2Z9S4	Putative enoyl-CoA hydratase/isomerase	-1,06	NA
PA14_44820	A0A0H2Z968	5-hydroxyisourate hydrolase (HIU hydrolase) (HIUHase) (EC 3.5.2.	-1,06	NA
PA14_05860	A0A0H2ZLD3	Putative long-chain acyl-CoA thioester hydrolase family protein	-1,06	NA
PA14_44290	A0A0H2Z808	Aconitate hydratase (Aconitase) (EC 4.2.1.3)	-1,06	NA
PA14_15590	A0A0H2ZEV3	Uncharacterized protein	-1,06	NA
PA14_30050	A0A0H2ZAX9	Putative isocitrate lyase	-1,06	NA
PA14_69190	A0A0H2ZHW8	Transcription termination factor Rho (EC 3.6.4.-) (ATP-dependent	-1,06	NA
PA14_08730	Q02T90	50S ribosomal protein L1	-1,06	NA
PA14_67860	A0A0H2ZIL0	Putative ABC-type amino acid transporter	-1,06	NA
PA14_58420	A0A0H2ZGV7	Putative binding protein component of ABC dipeptid	-1,06	NA
PA14_29940	A0A0H2ZAY4	NADH-quinone oxidoreductase (EC 1.6.5.11)	-1,06	NA
PA14_19140	A0A0H2ZEJ4	Cyclohexadienyl dehydratase	-1,06	NA
PA14_22830	A0A0H2ZDL3	Putative SUA5/yciO/yrdC family:Sua5/YciO/YrdC/YwlC protein fan	-1,06	NA
PA14_05690	A0A0H2ZLC4	Cytosine deaminase	-1,06	NA
PA14_64590	A0A0H2ZI81	Putative MFS transporter	-1,06	NA
PA14_57870	A0A0H2ZGJ7	Putative toluene tolerance ABC efflux transporter	-1,06	NA
PA14_31560	A0A0H2ZBW5	Putative transcriptional regulator, LysR family	-1,06	NA
PA14_20980	A0A0H2ZCS4	Putative short chain dehydrogenas	-1,06	NA
PA14_29130	A0A0H2ZB44	Putative ATPase, AAA family	-1,06	NA
PA14_27150	A0A0H2ZCK3	Putative ABC transporter, ATP-binding protein	-1,06	NA
PA14_15960	A0A0H2ZF03	Signal recognition particle protein (Fifty-four homolog)	-1,06	NA
PA14_36320	A0A0H2Z9M8	Hydrogen cyanide synthase HcnB	-1,06	NA
PA14_23050	A0A0H2ZDM8	Putative aldose 1-epimerase	-1,06	NA
PA14_58390	A0A0H2ZGN2	Putative binding protein component of ABC transpor	-1,06	NA
PA14_05560	A0A0H2ZK67	ATP-dependent RNA helicase RhlE (EC 3.6.4.13)	-1,06	NA
PA14_70190	Q02E46	50S ribosomal protein L28	-1,06	NA
PA14_53490	A0A0H2Z760	Uncharacterized protein	-1,06	NA
PA14_53820	A0A0H2Z601	Uncharacterized protein	-1,06	NA
PA14_13130	A0A0H2ZFP2	Uncharacterized protein	-1,06	NA
PA14_60890	A0A0H2ZH93	Serine hydroxymethyltransferase (SHMT) (Serine methylase) (EC 2	-1,06	NA
PA14_41575	A0A0H2ZM60	ECF sigma factor SigX	-1,06	NA
PA14_72650	A0A0H2ZJL7	Putative transcriptional regulator	-1,06	NA
PA14_70550	A0A0H2ZJ47	Putative epimerase	-1,06	NA
PA14_58220	A0A0H2ZGU1	Putative carboxymuconolactone decarboxylase	-1,06	NA
PA14_48520	A0A0H2Z8H2	Membrane-bound lytic murein transglycosylase A (EC 4.2.2.n1) (N	-1,06	NA
PA14_12050	Q02SH1	Ribosomal RNA large subunit methyltransferase H (EC 2.1.1.177) (	-1,06	NA
PA14_52670	A0A0H2Z7C1	N-succinylglutamate 5-semialdehyde dehydrogenase (EC 1.2.1.71	-1,06	NA

PA14_00090	Q02V73	Glycine--tRNA ligase beta subunit (EC 6.1.1.14) (Glycyl-tRNA synth	-1,06	NA
PA14_15080	A0A0H2ZE18	Putative iron-regulated membrane protein	-1,06	NA
PA14_05660	A0A0H2ZKY6	Putative transciptional regulator	-1,06	NA
PA14_48590	A0A0H2Z7Y2	Uncharacterized protein	-1,06	NA
PA14_54330	Q02HS2	Ribonuclease 3 (EC 3.1.26.3) (Ribonuclease III) (RNase III)	-1,06	NA
PA14_65660	A0A0H2ZIJ2	DNA topoisomerase 4 subunit B (EC 5.99.1.3) (Topoisomerase IV s	-1,06	NA
PA14_25370	A0A0H2ZD73	Uncharacterized protein	-1,06	NA
PA14_08740	Q02T89	50S ribosomal protein L10	-1,06	NA
PA14_01040	A0A0H2ZK14	Putative secretion protein	-1,06	NA
PA14_31470	A0A0H2ZBW9	Putative acyl-CoA synthetases (AMP-forming)	-1,06	NA
PA14_66010	A0A0H2ZI86	Putative oxidoreductase	-1,06	NA
PA14_29860	A0A0H2ZC99	NADH dehydrogenase I chain M	-1,06	NA
PA14_27560	A0A0H2ZCR6	Putative ATPase	-1,06	NA
PA14_66650	A0A0H2ZHG7	Type 4 fimbrial biogenesis protein PilN	-1,05	NA
PA14_62480	A0A0H2ZHV0	Aminotransferase (EC 2.6.1.-)	-1,05	NA
PA14_55750	A0A0H2ZGE0	Putative chemotaxis transducer	-1,05	NA
PA14_41020	A0A0H2Z9Q9	Putative Orn/Arg/Lys decarboxylase	-1,05	NA
PA14_67740	A0A0H2ZIW2	Glutaredoxin	-1,05	NA
PA14_26670	A0A0H2ZCZ1	Putative biotin carboxylase/biotin carboxyl carrier protein	-1,05	NA
PA14_07930	A0A0H2ZKK7	Phosphoglycolate phosphatase (PGP) (PGPase) (EC 3.1.3.18)	-1,05	NA
PA14_00290	A0A0H2ZJY6	Shikimate dehydrogenase (NADP(+)) (SDH) (EC 1.1.1.25)	-1,05	NA
PA14_70280	Q02E39	Acetylglutamate kinase (EC 2.7.2.8) (N-acetyl-L-glutamate 5-phosp	-1,05	NA
PA14_67900	A0A0H2ZIX0	Uncharacterized protein	-1,05	NA
PA14_45930	A0A0H2Z8Y4	Uncharacterized protein	-1,05	NA
PA14_25280	Q02PG3	Na(+)-translocating NADH-quinone reductase subunit A (Na(+)-NC	-1,05	NA
PA14_29470	A0A0H2ZCB9	Uncharacterized protein	-1,05	NA
PA14_16460	A0A0H2ZEQ5	CheW domain protein	-1,05	NA
PA14_57570	A0A0H2ZGP4	Ubiquinol-cytochrome c reductase iron-sulfur subunit (EC 1.10.2.2	-1,05	NA
PA14_35850	A0A0H2ZB06	Uncharacterized protein	-1,05	NA
PA14_65540	A0A0H2ZHA6	Uncharacterized protein	-1,05	NA
PA14_41360	Q02KT6	Cysteine--tRNA ligase (EC 6.1.1.16) (Cysteinyl-tRNA synthetase) (C	-1,05	NA
PA14_27370	A0A0H2ZCI6	ATP-dependent RNA helicase DeaD (EC 3.6.4.13) (Cold-shock DEA	-1,05	NA
PA14_25250	Q02PG5	Glyceraldehyde-3-phosphate dehydrogenase-like protein (EC 1.2.1	-1,05	NA
PA14_00010	Q02V80	Chromosomal replication initiator protein DnaA	-1,05	NA
PA14_04630	Q02U62	Dihydroxy-acid dehydratase (DAD) (EC 4.2.1.9)	-1,05	NA
PA14_56080	A0A0H2ZGF7	Exodeoxyribonuclease I (EC 3.1.11.1)	-1,05	NA
PA14_46920	A0A0H2Z8S4	Putative permease of ABC transporter	-1,05	NA
PA14_58190	Q02GV7	Aspartyl/glutamyl-tRNA(Asn/Gln) amidotransferase subunit B (Asp	-1,05	NA
PA14_20750	A0A0H2ZDT9	Putative chemotaxis signal transduction protein	-1,05	NA
PA14_14700	A0A0H2ZF79	Serine O-acetyltransferase	-1,05	NA
PA14_13090	A0A0H2ZFK4	Probable 3-ketoacyl-CoA thiolase	-1,05	NA
PA14_47360	A0A0H2Z7A7	Putative oligopeptidase	-1,05	NA
PA14_47750	A0A0H2Z782	Putative nitroreductase	-1,05	NA
PA14_19230	A0A0H2ZEI9	Putative mechanosensitive ion channel family protein	-1,05	NA
PA14_09020	Q02T63	30S ribosomal protein S5	-1,05	NA
PA14_67090	Q02ET9	Glucans biosynthesis protein G	-1,05	NA
PA14_04380	A0A0H2ZL29	Putative hydrolase	-1,05	NA
PA14_51290	A0A0H2Z7L7	Bacterioferritin comigratory protein	-1,05	NA
PA14_65150	Q02F86	50S ribosomal protein L9	-1,05	NA
PA14_13350	A0A0H2ZEC4	Uncharacterized protein	-1,05	NA
PA14_68070	A0A0H2ZIL8	Putative ABC transporter, periplasmic amino acid-binding protein	-1,05	NA
PA14_69660	A0A0H2ZI37	Lipopeptide LppL	-1,05	NA
PA14_50290	A0A0H2Z7X1	Flagellin	-1,05	NA
PA14_61700	Q02G09	Peptide chain release factor 1 (RF-1)	-1,05	NA
PA14_03480	A0A0H2ZKX0	Putative transcriptional regulator, GntR family	-1,05	NA
PA14_30210	Q02NB3	ATP-dependent Clp protease adapter protein ClpS	-1,05	NA
PA14_07760	A0A0H2ZLB3	Chaperone SurA (Peptidyl-prolyl cis-trans isomerase SurA) (Rotam	-1,05	NA
PA14_25350	Q02PF8	Na(+)-translocating NADH-quinone reductase subunit F (Na(+)-NC	-1,05	NA
PA14_62450	Q02FV3	tRNA/tmRNA (uracil-C(5))-methyltransferase (EC 2.1.1.-) (EC 2.1.1	-1,05	NA

PA14_61820	A0A0H2ZHQ7	Ribosome-binding ATPase YchF	-1,05	NA
PA14_21790	Q02Q95	Recombination-associated protein RdgC	-1,05	NA
PA14_56830	A0A0H2ZGD9	Insulin-cleaving metalloproteinase outer membrane	-1,05	NA
PA14_16800	A0A0H2ZEL2	Putative efflux transmembrane protein	-1,05	NA
PA14_43970	A0A0H2Z9F5	Dihydrolipoyl dehydrogenase (EC 1.8.1.4)	-1,05	NA
PA14_66240	A0A0H2ZID3	Lipopolysaccharide heptosyltransferase I	-1,05	NA
PA14_16630	A0A0H2ZEX2	Putative outer membrane protein, OmpA	-1,05	NA
PA14_23330	A0A0H2ZDD5	30S ribosomal protein S1	-1,05	NA
PA14_66600	Q02EX9	3-dehydroquinate synthase (EC 4.2.3.4)	-1,05	NA
PA14_06500	Q02TR6	Biotin synthase (EC 2.8.1.6)	-1,05	NA
PA14_14500	A0A0H2ZF09	Putative permease	-1,05	NA
PA14_08990	Q02T66	30S ribosomal protein S8	-1,05	NA
PA14_29760	A0A0H2ZC87	Putative chemotaxis transducer	-1,05	NA
PA14_62760	Q02FS8	Translation initiation factor IF-2	-1,05	NA
PA14_23220	Q02PX7	Ubiquinone biosynthesis O-methyltransferase (2-polyprenyl-6-hy	-1,05	NA
PA14_66290	A0A0H2ZIL4	Pyruvate dehydrogenase E1 component (EC 1.2.4.1)	-1,05	NA
PA14_16130	A0A0H2ZEZ5	Putative carnitine dehydratase	-1,05	NA
PA14_65110	A0A0H2ZIG8	Alanine racemase (EC 5.1.1.1)	-1,05	NA
PA14_24220	Q02PQ1	NAD kinase (EC 2.7.1.23) (ATP-dependent NAD kinase)	-1,05	NA
PA14_01010	A0A0H2ZK88	Uncharacterized protein	-1,05	NA
PA14_16090	A0A0H2ZET1	Threonine synthase	-1,05	NA
PA14_01470	A0A0H2ZJU3	Putative transcriptional regulator	-1,05	NA
PA14_07690	Q02TH4	Thiosulfate sulfurtransferase GlpE (EC 2.8.1.1)	-1,05	NA
PA14_57060	A0A0H2ZGN0	Uncharacterized protein	-1,05	NA
PA14_53190	A0A0H2Z769	Morphogene protein BolA	-1,05	NA
PA14_69580	A0A0H2ZJ96	Uncharacterized protein	-1,05	NA
PA14_50590	A0A0H2Z6Q5	Probable heat shock protein (Hsp90 family)	-1,05	NA
PA14_54430	A0A0H2Z5X2	RNA polymerase sigma factor	-1,05	NA
PA14_50560	A0A0H2Z7U9	High-affinity branched-chain amino acid transport ATP-binding pr	-1,05	NA
PA14_62020	A0A0H2ZHP1	Paraquat-inducible protein B-like protein	-1,05	NA
PA14_44660	Q02K12	DNA ligase (EC 6.5.1.2) (Polydeoxyribonucleotide synthase [NAD(	-1,05	NA
PA14_22860	A0A0H2ZDF2	Segregation and condensation protein B	-1,05	NA
PA14_69420	A0A0H2ZJ68	Putative enzyme of heme biosynthesis	-1,05	NA
PA14_05280	Q02U08	Putative pre-16S rRNA nuclease (EC 3.1.-.-)	-1,05	NA
PA14_11690	A0A0H2ZFK1	Inorganic pyrophosphatase (EC 3.6.1.1) (Pyrophosphate phospho	-1,05	NA
PA14_19410	A0A0H2ZE52	Uncharacterized protein	-1,05	NA
PA14_57940	A0A0H2ZGR9	RNA polymerase sigma-54 factor	-1,05	NA
PA14_13410	Q02S69	Peptide chain release factor 3 (RF-3)	-1,05	NA
PA14_68370	A0A0H2ZIZ4	3'(2'),5'-bisphosphate nucleotidase	-1,05	NA
PA14_52060	A0A0H2Z7P7	Uncharacterized protein	-1,05	NA
PA14_04480	Q02U73	Thymidylate synthase (TS) (TSase) (EC 2.1.1.45)	-1,05	NA
PA14_07040	A0A0H2ZKT4	Putative exported protein	-1,05	NA
PA14_68360	A0A0H2ZIZ0	Putative beta-ketoacyl synthase	-1,05	NA
PA14_66670	A0A0H2ZIP9	Penicillin-binding protein 1A	-1,05	NA
PA14_57670	A0A0H2ZGQ0	Tryptophan--tRNA ligase (EC 6.1.1.2) (Tryptophanyl-tRNA synthet	-1,05	NA
PA14_07230	A0A0H2ZKH3	Fructose-1,6-bisphosphate aldolase	-1,05	NA
PA14_21160	Q02QE8	Phosphonates import ATP-binding protein PhnC 2 (EC 3.6.3.28)	-1,05	NA
PA14_64480	A0A0H2ZIC6	Osmotically inducible lipoprotein OsmE	-1,05	NA
PA14_56750	A0A0H2ZFH6	Uncharacterized protein	-1,05	NA
PA14_19130	A0A0H2ZEE6	Acyl-homoserine-lactone synthase (EC 2.3.1.184) (Autoinducer sy	-1,05	NA
PA14_60040	A0A0H2ZHB1	Uncharacterized protein	-1,05	NA
PA14_15740	A0A0H2ZDW9	Phosphoribosylformylglycinamidine synthase (FGAM synthase) (F	-1,05	NA
PA14_72400	A0A0H2ZIP7	Putative N-acetylmuramoyl-L-alanine amidase family protein	-1,05	NA
PA14_70360	A0A0H2ZJ40	Uncharacterized protein	-1,05	NA
PA14_24900	A0A0H2ZBX7	Molybdenum cofactor biosynthesis protein B	-1,05	NA
PA14_69430	A0A0H2ZJ90	Putative uroporphyrin-III C-methyltransferase	-1,05	NA
PA14_30990	A0A0H2ZAN6	Uncharacterized protein	-1,05	NA
PA14_73170	A0A0H2ZJZ3	Glutamine--fructose-6-phosphate aminotransferase [isomerizing]	-1,05	NA
PA14_24980	A0A0H2ZD90	Uncharacterized protein	-1,05	NA

PA14_27850	Q02NW3	NADPH-dependent 7-cyano-7-deazaguanine reductase (EC 1.7.1.1	-1,05	NA
PA14_57850	A0A0H2ZFP5	Uncharacterized protein	-1,04	NA
PA14_30160	A0A0H2ZC83	Putative NUDIX hydrolase	-1,04	NA
PA14_23800	A0A0H2ZDK9	Aspartate-semialdehyde dehydrogenase (ASA dehydrogenase) (A:	-1,04	NA
PA14_65450	A0A0H2ZI46	Chemotaxis protein MotA	-1,04	NA
PA14_05330	A0A0H2ZKW2	Type IV pilus response regulator PilH	-1,04	NA
PA14_51440	A0A0H2Z6E9	Methylated-DNA--protein-cysteine methyltransferase (EC 2.1.1.63	-1,04	NA
PA14_68480	A0A0H2ZJ04	Chorismate mutase (EC 5.4.99.5)	-1,04	NA
PA14_65410	Q02F67	Oligoribonuclease (EC 3.1.-.-)	-1,04	NA
PA14_60230	A0A0H2ZHK5	Outer membrane protein assembly factor BamD	-1,04	NA
PA14_65750	A0A0H2ZIF7	Putative outer membrane efflux protein	-1,04	NA
PA14_45950	A0A0H2Z7N2	Regulatory protein RsaL	-1,04	NA
PA14_23670	A0A0H2ZC77	Putative bleomycin resistance protein	-1,04	NA
PA14_61780	Q02G03	50S ribosomal protein L25 (General stress protein CTC)	-1,04	NA
PA14_09700	A0A0H2ZGC7	Putative FAD-dependent monooxygenase	-1,04	NA
PA14_66900	Q02EV4	Ubiquinone/menaquinone biosynthesis C-methyltransferase UbiE	-1,04	NA
PA14_12280	Q02SF2	Apolipoprotein N-acyltransferase (ALP N-acyltransferase) (EC 2.3.:	-1,04	NA
PA14_70010	A0A0H2ZJD3	Putative endoribonuclease L-PSP family protein	-1,04	NA
PA14_23420	A0A0H2ZDI1	Putative zinc-binding dehydrogenase	-1,04	NA
PA14_13620	A0A0H2ZEA6	Na+/H+ antiporter NhaP	-1,04	NA
PA14_05410	A0A0H2ZLA3	Putative chemotaxis protein methyltransferase CheR	-1,04	NA
PA14_66910	A0A0H2ZHH9	Uncharacterized protein	-1,04	NA
PA14_62900	A0A0H2ZHW6	Transcription elongation factor GreA (Transcript cleavage factor G	-1,04	NA
PA14_58320	A0A0H2ZGM8	Putative two-component sensor	-1,04	NA
PA14_05550	A0A0H2ZKJ8	Major intrinsic multiple antibiotic resistance efflux outer membra	-1,04	NA
PA14_62870	Q02FS0	Ribosomal RNA large subunit methyltransferase E (EC 2.1.1.166) (	-1,04	NA
PA14_46620	A0A0H2Z7H2	Putative pyridine nucleotide-disulphide oxidoreductase	-1,04	NA
PA14_17050	A0A0H2ZER3	Methionine aminopeptidase (MAP) (MetAP) (EC 3.4.11.18) (Peptic	-1,04	NA
PA14_15600	A0A0H2ZDX8	Uncharacterized protein	-1,04	NA
PA14_42670	A0A0H2Z958	Putative membrane protein	-1,04	NA
PA14_21175	A0A0H2ZLM2	Putative ABC-type phosphate/phosphonate transport system, per	-1,04	NA
PA14_60210	A0A0H2ZH46	Pseudouridine synthase (EC 5.4.99.-)	-1,04	NA
PA14_04330	A0A0H2ZKD8	Putative integral membrane protein	-1,04	NA
PA14_68560	A0A0H2ZIR2	Putative nitroreductase	-1,04	NA
PA14_62260	A0A0H2ZHT5	Uncharacterized protein	-1,04	NA
PA14_05340	A0A0H2ZL98	Type IV pili signal transduction protein PilI	-1,04	NA
PA14_26940	A0A0H2ZCL8	Uncharacterized protein	-1,04	NA
PA14_38500	A0A0H2ZAG7	Putative transcriptional regulator, IclR family	-1,04	NA
PA14_41210	A0A0H2Z9H8	DNA-binding protein HU	-1,04	NA
PA14_67890	Q02EM5	1-(5-phosphoribosyl)-5-[(5-phosphoribosylamino)methylideneam	-1,04	NA
PA14_50750	A0A0H2Z800	Uncharacterized protein	-1,04	NA
PA14_03510	A0A0H2ZJR8	Uncharacterized protein	-1,04	NA
PA14_22270	A0A0H2ZCH4	Possible recombinase	-1,04	NA
PA14_04320	A0A0H2ZL26	L-threonine dehydratase (EC 4.3.1.19) (Threonine deaminase)	-1,04	NA
PA14_61860	A0A0H2ZHI9	Carbonic anhydrase (EC 4.2.1.1) (Carbonate dehydratase)	-1,04	NA
PA14_63550	A0A0H2ZHX4	Protein FdhE homolog	-1,04	NA
PA14_60370	Q02GB7	Isoleucine--tRNA ligase (EC 6.1.1.5) (Isoleucyl-tRNA synthetase) (Il	-1,04	NA
PA14_61150	A0A0H2ZHH2	Putative oxidoreductase	-1,04	NA
PA14_50820	A0A0H2Z7Z7	Probable esterase	-1,04	NA
PA14_00200	A0A0H2ZK18	Peptide deformylase (PDF) (EC 3.5.1.88) (Polypeptide deformylase	-1,04	NA
PA14_02830	A0A0H2ZKG5	3-carboxy-cis,cis-muconate cycloisomerase	-1,04	NA
PA14_65270	A0A0H2ZIC7	Protein HflC	-1,04	NA
PA14_57010	Q02H55	60 kDa chaperonin (GroEL protein) (Protein Cpn60)	-1,04	NA
PA14_17070	Q02RC7	Elongation factor Ts (EF-Ts)	-1,04	NA
PA14_19620	A0A0H2ZEG5	D-erythro-7,8-dihydroneopterin triphosphate epimerase	-1,04	NA
PA14_63605	A0A0H2ZL50	Formate dehydrogenase-O, major subunit	-1,04	NA
PA14_12210	A0A0H2ZFQ7	LPS-assembly lipoprotein LptE	-1,04	NA
PA14_53260	A0A0H2Z710	Uncharacterized protein	-1,04	NA
PA14_07770	Q02TG8	LPS-assembly protein LptD	-1,04	NA

PA14_41610	A0A0H2Z9X3	Uncharacterized protein	-1,04	NA
PA14_73330	A0A0H2ZJW9	Chromosome partitioning protein	-1,04	NA
PA14_60990	A0A0H2ZHH7	DNA repair protein RadA	-1,04	NA
PA14_06870	A0A0H2ZKS2	Transcriptional regulator Dnr	-1,04	NA
PA14_54170	A0A0H2Z6N1	Bifunctional protein PutA	-1,04	NA
PA14_69450	Q02EA3	Porphobilinogen deaminase (PBG) (EC 2.5.1.61) (Hydroxymethylb	-1,04	NA
PA14_05540	A0A0H2ZLB4	RND multidrug efflux transporter MexB	-1,04	NA
PA14_47010	A0A0H2Z8J4	Uncharacterized protein	-1,04	NA
PA14_25210	A0A0H2ZBV8	Probable S-methyl-5'-thioinosine phosphorylase (EC 2.4.2.44) (5'-I	-1,04	NA
PA14_16070	A0A0H2ZF44	Homoserine dehydrogenase (EC 1.1.1.3)	-1,04	NA
PA14_31890	A0A0H2ZBU6	Putative RND efflux transporter	-1,04	NA
PA14_16190	A0A0H2ZEZ3	Uncharacterized protein	-1,04	NA
PA14_11250	A0A0H2ZFV6	Putative dTDP-4-rhamnose reductase-related protein	-1,04	NA
PA14_66060	Q02F21	Bifunctional protein HldE [Includes: D-beta-D-heptose 7-phosphat	-1,04	NA
PA14_23880	A0A0H2ZDG0	Folylpolyglutamate synthetase	-1,04	NA
PA14_56300	A0A0H2ZFE6	Putative fumarase	-1,04	NA
PA14_57370	A0A0H2ZGX6	UDP-N-acetylmuramoylalanine--D-glutamate ligase (EC 6.3.2.9) (D	-1,04	NA
PA14_63340	A0A0H2ZHZ2	Putative lipoprotein	-1,04	NA
PA14_24170	A0A0H2ZDE2	2,4-dienoyl-CoA reductase FadH1	-1,04	NA
PA14_14740	A0A0H2ZEZ6	Iron-sulfur cluster assembly scaffold protein IscU	-1,04	NA
PA14_08920	Q02T73	50S ribosomal protein L16	-1,04	NA
PA14_54210	A0A0H2Z5Y3	Lon protease (EC 3.4.21.53) (ATP-dependent protease La)	-1,04	NA
PA14_56510	A0A0H2ZGR6	Uncharacterized protein	-1,04	NA
PA14_58150	A0A0H2ZGL8	Rod shape-determining protein MreB	-1,04	NA
PA14_00190	Q02V63	Methionyl-tRNA formyltransferase (EC 2.1.2.9)	-1,04	NA
PA14_54260	Q02HS7	Carboxy-S-adenosyl-L-methionine synthase (Cx-SAM synthase) (E(	-1,04	NA
PA14_04140	A0A0H2ZJW5	Putative oxidoreductase, FAD-binding	-1,04	NA
PA14_07590	A0A0H2ZKJ2	7,8-dihydroneopterin aldolase (EC 4.1.2.25)	-1,04	NA
PA14_60380	A0A0H2ZHL4	Riboflavin biosynthesis protein (EC 2.7.1.26) (EC 2.7.7.2)	-1,04	NA
PA14_66630	A0A0H2ZIN8	Type 4 fimbrial biogenesis protein PilP	-1,04	NA
PA14_49130	Q02J03	C4-dicarboxylate transport protein 2	-1,04	NA
PA14_44610	Q02K17	Recombination protein RecR	-1,04	NA
PA14_25140	A0A0H2ZD62	Uncharacterized protein	-1,04	NA
PA14_12020	Q02SH3	Probable nicotinate-nucleotide adenylyltransferase (EC 2.7.7.18) (	-1,04	NA
PA14_62180	A0A0H2ZHZ6	Uncharacterized protein	-1,04	NA
PA14_66880	A0A0H2ZIQ5	Putative poly(Hydroxyalcanoate) granule associated protein	-1,04	NA
PA14_56010	A0A0H2ZGN3	Chemotactic transducer PctB	-1,04	NA
PA14_46120	A0A0H2Z8W5	Putative decarboxylase	-1,04	NA
PA14_46970	A0A0H2Z7E2	Glutaminase-asparaginase	-1,04	NA
PA14_10040	A0A0H2ZFZ0	Amidase family protein	-1,04	NA
PA14_18230	A0A0H2ZEL0	Transcriptional repressor of fru operon	-1,04	NA
PA14_57530	A0A0H2ZFM5	Stringent starvation protein A	-1,04	NA
PA14_55980	A0A0H2ZFC2	Uncharacterized protein	-1,04	NA
PA14_58080	A0A0H2ZFR8	Putative carbon-nitrogen hydrolase	-1,04	NA
PA14_23060	A0A0H2ZDJ9	Putative transcriptional regulator, RpiR family	-1,04	NA
PA14_17310	Q02RA8	2-dehydro-3-deoxyphosphooctonate aldolase (EC 2.5.1.55) (3-dec	-1,04	NA
PA14_12030	Q02SH2	Ribosomal silencing factor RsfS	-1,04	NA
PA14_57740	A0A0H2ZGQ5	GTP cyclohydrolase 1 type 2 homolog	-1,04	NA
PA14_14590	Q02RX8	S-adenosylmethionine:tRNA ribosyltransferase-isomerase (EC 2.4.	-1,04	NA
PA14_62770	A0A0H2ZI23	Transcription termination/antitermination protein NusA	-1,04	NA
PA14_51480	A0A0H2Z7J1	Uncharacterized protein	-1,04	NA
PA14_07660	Q02TH6	UPF0229 protein PA14_07660	-1,04	NA
PA14_44140	Q02K58	UPF0345 protein PA14_44140	-1,04	NA
PA14_24910	A0A0H2ZD93	Molybdenum cofactor biosynthesis protein A2	-1,04	NA
PA14_21820	A0A0H2ZDL5	Peptidyl-prolyl cis-trans isomerase (EC 5.2.1.8)	-1,04	NA
PA14_26510	A0A0H2ZCZ6	Precorrin-2 methyltransferase CobI	-1,04	NA
PA14_60470	A0A0H2ZGA4	Octylprenyl diphosphate synthase	-1,04	NA
PA14_20700	A0A0H2ZCU7	Putative glycosyltransferase	-1,04	NA
PA14_19205	A0A0H2ZM79	Uncharacterized protein	-1,04	NA

PA14_69050	A0A0H2ZIT4	Uncharacterized protein	-1,04	NA
PA14_26860	A0A0H2ZCW8	Putative transcriptional regulator, LysR family	-1,04	NA
PA14_44780	A0A0H2Z978	Putative transcriptional regulator	-1,04	NA
PA14_41920	A0A0H2Z9V4	Phospho-2-dehydro-3-deoxyheptonate aldolase (EC 2.5.1.54) (3-d	-1,04	NA
PA14_67100	Q02ET8	D-aminoacyl-tRNA deacylase (EC 3.1.1.96) (D-tyrosyl-tRNA(Tyr) de	-1,04	NA
PA14_69940	Q02E64	Xanthine phosphoribosyltransferase (XPRTase) (EC 2.4.2.22)	-1,04	NA
PA14_69230	A0A0H2ZJ53	Polyphosphate kinase (EC 2.7.4.1) (ATP-polyphosphate phosphotr	-1,04	NA
PA14_16590	A0A0H2ZEW9	Uncharacterized protein	-1,04	NA
PA14_65720	A0A0H2ZIJ5	Putative lipoprotein	-1,04	NA
PA14_17260	A0A0H2ZEI5	DNA-directed DNA polymerase (EC 2.7.7.7)	-1,04	NA
PA14_12390	Q02SE5	Glutamate-1-semialdehyde 2,1-aminomutase (GSA) (EC 5.4.3.8) ((	-1,04	NA
PA14_51205	A0A0H2ZM53	Probable transcriptional regulator	-1,04	NA
PA14_08820	A0A0H2ZGD8	Elongation factor G (EF-G)	-1,04	NA
PA14_45540	A0A0H2Z930	Chemotaxis protein, MotD	-1,04	NA
PA14_57210	A0A0H2ZFL0	Arginine biosynthesis bifunctional protein ArgJ	-1,04	NA
PA14_60310	Q02GC2	Type IV pilus biogenesis factor PilY1 (Pilus-associated adhesin PilY	-1,04	NA
PA14_16870	A0A0H2ZEW2	Putative ABC transporter ATP-binding protein	-1,04	NA
PA14_52040	Q02IA6	Phosphoribosylformylglycinamidine cyclo-ligase (EC 6.3.3.1) (AIR s	-1,04	NA
PA14_23270	Q02PX3	Phosphoserine aminotransferase (EC 2.6.1.52) (Phosphohydroxytl	-1,04	NA
PA14_52270	A0A0H2Z739	D-lactate dehydrogenase (Fermentative)	-1,04	NA
PA14_58380	A0A0H2ZFU5	Putative transcriptional regulator	-1,04	NA
PA14_72380	A0A0H2ZJR9	Two-component response regulator AlgB	-1,04	NA
PA14_20670	A0A0H2ZE30	Putative glutamine synthetase	-1,04	NA
PA14_49800	A0A0H2Z884	Probable oxidoreductase	-1,03	NA
PA14_38530	A0A0H2ZA54	Fumarylacetoacetase	-1,03	NA
PA14_38460	A0A0H2ZA59	Acyl-CoA carboxyltransferase beta chain	-1,03	NA
PA14_62490	A0A0H2ZHR6	RNA polymerase-binding transcription factor DksA	-1,03	NA
PA14_57840	A0A0H2ZGS4	Putative toluene tolerance protein	-1,03	NA
PA14_05480	A0A0H2ZKJ4	Putative monoamine oxidase	-1,03	NA
PA14_64500	A0A0H2ZI28	Putative transcriptional regulator	-1,03	NA
PA14_69500	Q02EA0	Argininosuccinate lyase (ASAL) (EC 4.3.2.1) (Arginosuccinase)	-1,03	NA
PA14_68660	Q02EG7	Probable alpha-L-glutamate ligase (EC 6.3.2.-)	-1,03	NA
PA14_23830	A0A0H2ZC68	Pilus assembly protein	-1,03	NA
PA14_07940	A0A0H2ZLM7	Anthranilate synthetase component I	-1,03	NA
PA14_62130	Q02FX9	Ketol-acid reductoisomerase (NADP(+)) (KARI) (EC 1.1.1.86) (Aceto	-1,03	NA
PA14_08680	Q02T82	Elongation factor Tu (EF-Tu)	-1,03	NA
PA14_46360	A0A0H2Z8U2	Putative two-component response regulator	-1,03	NA
PA14_62910	A0A0H2ZHT6	Carbamoyl-phosphate synthase (glutamine-hydrolyzing) (EC 6.3.5	-1,03	NA
PA14_46280	A0A0H2Z8X6	Uncharacterized protein	-1,03	NA
PA14_52180	A0A0H2Z7P3	GTP pyrophosphokinase	-1,03	NA
PA14_62160	A0A0H2ZGQ7	Acetolactate synthase (EC 2.2.1.6)	-1,03	NA
PA14_17460	Q02R96	Protein-L-isoaspartate O-methyltransferase (EC 2.1.1.77) (L-isoasp	-1,03	NA
PA14_60950	A0A0H2ZGE5	Carbon starvation protein	-1,03	NA
PA14_00470	A0A0H2ZIY0	Uncharacterized protein	-1,03	NA
PA14_36300	A0A0H2ZAT6	Putative transcriptional regulator, TetR family	-1,03	NA
PA14_57520	A0A0H2ZGQ3	Stringent starvation protein B	-1,03	NA
PA14_30730	A0A0H2ZC51	Uncharacterized protein	-1,03	NA
PA14_65200	A0A0H2ZI34	Ribonuclease R (RNase R) (EC 3.1.13.1)	-1,03	NA
PA14_60120	A0A0H2ZH41	Putative deoxycytidine deaminase	-1,03	NA
PA14_41170	A0A0H2Z8P5	Enoyl-[acyl-carrier-protein] reductase [NADH] (EC 1.3.1.9)	-1,03	NA
PA14_67560	A0A0H2ZIV2	GTP-binding protein TypA/BipA	-1,03	NA
PA14_15310	A0A0H2ZEW8	Inosine-5'-monophosphate dehydrogenase (IMP dehydrogenase)	-1,03	NA
PA14_66875	A0A0H2ZKV4	Polyhydroxyalkanoate synthesis protein PhaF	-1,03	NA
PA14_05110	A0A0H2ZKU7	Uncharacterized protein	-1,03	NA
PA14_42020	A0A0H2Z8J3	Putative glutaredoxin	-1,03	NA
PA14_58760	A0A0H2ZGY4	Type 4 fimbrial biogenesis protein pilC	-1,03	NA
PA14_61130	A0A0H2ZHQ5	Putative cytochrome c	-1,03	NA
PA14_07710	Q02TH2	Protein ApaG	-1,03	NA
PA14_41220	A0A0H2Z9W7	Lon protease (EC 3.4.21.53) (ATP-dependent protease La)	-1,03	NA

PA14_73290	Q02DF0	ATP synthase subunit b (ATP synthase F(0) sector subunit b) (ATPa	-1,03	NA
PA14_70440	A0A0H2ZJE1	Guanylate kinase (EC 2.7.4.8) (GMP kinase)	-1,03	NA
PA14_04730	A0A0H2ZK10	Probable gamma-glutamyltranspeptidase	-1,03	NA
PA14_46300	A0A0H2Z8U7	Putative O-methyltransferase	-1,03	NA
PA14_20850	A0A0H2ZE38	Putative transcriptional regulator, MarR family	-1,03	NA
PA14_44630	A0A0H2Z990	DNA polymerase subunits gamma and tau	-1,03	NA
PA14_51980	A0A0H2Z6A9	Arsenate reductase (EC 1.20.4.1)	-1,03	NA
PA14_67110	A0A0H2ZIR6	Proline iminopeptidase (EC 3.4.11.5)	-1,03	NA
PA14_52800	A0A0H2Z7A9	Acetyl-coenzyme A synthetase (AcCoA synthetase) (Acs) (EC 6.2.1.	-1,03	NA
PA14_23310	A0A0H2ZDN7	3-phosphoshikimate 1-carboxyvinyltransferase (EC 2.5.1.19)	-1,03	NA
PA14_66620	A0A0H2ZIP5	Type 4 fimbrial biogenesis outer membrane protein PilQ	-1,03	NA
PA14_69470	A0A0H2ZI20	Alginate biosynthesis regulatory protein AlgR	-1,03	NA
PA14_09630	A0A0H2ZG42	Putative acyl-CoA dehydrogenase	-1,03	NA
PA14_39790	A0A0H2ZA02	Uncharacterized protein	-1,03	NA
PA14_18920	A0A0H2ZEK4	Electron transport complex subunit C	-1,03	NA
PA14_58450	A0A0H2ZFV0	Dipeptide ABC transporter, permease protein	-1,03	NA
PA14_19090	Q02QW9	Deoxycytidine triphosphate deaminase (dCTP deaminase) (EC 3.5.	-1,03	NA
PA14_57050	A0A0H2ZGM0	Putative short chain dehydrogenase	-1,03	NA
PA14_67065	Q02EU0	Glucans biosynthesis glucosyltransferase H (EC 2.4.1.-)	-1,03	NA
PA14_30710	A0A0H2ZC37	Putative activator of osmoprotectant transporter	-1,03	NA
PA14_27960	Q02NV3	Transaldolase (EC 2.2.1.2)	-1,03	NA
PA14_05310	A0A0H2ZK52	Glutathione synthetase (EC 6.3.2.3) (GSH synthetase) (Glutathione	-1,03	NA
PA14_61590	A0A0H2ZHM1	Putative sugar nucleotide epimerase	-1,03	NA
PA14_63060	Q02FQ4	SsrA-binding protein (Small protein B)	-1,03	NA
PA14_17670	A0A0H2ZEN3	Putative DNA-binding response regulator, LuxR family	-1,03	NA
PA14_09820	A0A0H2ZGB4	Putative acetolactate synthase large subunit	-1,03	NA
PA14_54150	A0A0H2Z7B9	Sodium/proline symporter PutP	-1,03	NA
PA14_29770	A0A0H2ZCA2	Putative permease	-1,03	NA
PA14_12450	A0A0H2ZEI7	Possible acyl-CoA dehydrogenase	-1,03	NA
PA14_66090	A0A0H2ZI91	Putative toluene tolerance protein	-1,03	NA
PA14_17530	Q02R89	Protein RecA (Recombinase A)	-1,03	NA
PA14_69700	A0A0H2ZJB6	Uncharacterized protein	-1,03	NA
PA14_14930	Q02RV3	GTPase Der (GTP-binding protein EngA)	-1,03	NA
PA14_13510	A0A0H2ZFG2	Putative transcriptional regulator, LysR family	-1,03	NA
PA14_23030	A0A0H2ZCB8	Porin	-1,03	NA
PA14_29110	A0A0H2ZCD7	Cysteine synthase (EC 2.5.1.47)	-1,03	NA
PA14_09130	Q02T54	50S ribosomal protein L17	-1,03	NA
PA14_43340	A0A0H2Z9H2	Two-component response regulator KdpE	-1,03	NA
PA14_57930	A0A0H2ZH13	Putative ATP-binding component of ABC transporter	-1,03	NA
PA14_46950	A0A0H2Z8P3	Putative ATP-binding component of ABC transporter	-1,03	NA
PA14_69240	A0A0H2ZIV9	Delta-aminolevulinic acid dehydratase (EC 4.2.1.24)	-1,03	NA
PA14_33320	Q02MM0	CRISPR-associated protein Csy2	-1,03	NA
PA14_04890	A0A0H2ZL01	Putative zinc protease	-1,03	NA
PA14_60420	Q02GB2	Glutamate 5-kinase (EC 2.7.2.11) (Gamma-glutamyl kinase) (GK)	-1,03	NA
PA14_04030	A0A0H2ZJV5	Putative lysophospholipase	-1,03	NA
PA14_01100	A0A0H2ZJ28	Putative ClpA/B-type chaperone	-1,03	NA
PA14_67490	Q02EQ6	Fructose-1,6-bisphosphatase class 1 (FBPase class 1) (EC 3.1.3.11)	-1,03	NA
PA14_61650	A0A0H2ZHM8	Lipid A deacylase (EC 3.1.1.77) (LPS 3-O-deacylase) (Outer membr	-1,03	NA
PA14_04870	A0A0H2ZK22	Putative peptidase	-1,03	NA
PA14_14780	Q02RW4	Chaperone protein HscA homolog	-1,03	NA
PA14_65470	A0A0H2ZII2	Uncharacterized protein	-1,03	NA
PA14_03430	A0A0H2ZJR3	Succinate-semialdehyde dehydrogenase, NADP-dependent activit	-1,03	NA
PA14_24270	A0A0H2ZDF7	Aminopeptidase N	-1,03	NA
PA14_68090	A0A0H2ZIX8	Putative amino acid ABC transporter, permease protein	-1,03	NA
PA14_55770	A0A0H2ZGE9	Phosphate transporter	-1,03	NA
PA14_63020	A0A0H2ZHX0	Ferric uptake regulation protein	-1,03	NA
PA14_09000	Q02T65	50S ribosomal protein L6	-1,03	NA
PA14_62540	A0A0H2ZI15	Two-component response regulator CbrB	-1,03	NA
PA14_20970	A0A0H2ZDS4	Cytochrome P450	-1,03	NA

PA14_19065	A0A0H2ZL81	Iron-sulfur cluster carrier protein	-1,03	NA
PA14_66680	A0A0H2ZIP4	Malic enzyme	-1,03	NA
PA14_23290	A0A0H2ZDI8	Histidinol-phosphate aminotransferase (EC 2.6.1.9) (Imidazole ace	-1,03	NA
PA14_16700	Q02RF8	Adenylate kinase (AK) (EC 2.7.4.3) (ATP-AMP transphosphorylase)	-1,03	NA
PA14_44490	A0A0H2Z8S5	Transcriptional regulator Anr	-1,03	NA
PA14_27870	A0A0H2ZCF0	Uncharacterized protein	-1,03	NA
PA14_09260	A0A0H2ZGD1	Transcriptional regulator PchR	-1,03	NA
PA14_22470	A0A0H2ZDR5	Putative transcriptional regulator, LysR family	-1,03	NA
PA14_52130	A0A0H2Z748	Uncharacterized protein	-1,03	NA
PA14_27220	A0A0H2ZCJ7	Organic hydroperoxide resistance protein	-1,03	NA
PA14_03490	A0A0H2ZK86	Alkyl hydroperoxide reductase AhpD (EC 1.11.1.15)	-1,03	NA
PA14_72720	A0A0H2ZJM1	Putative C4-dicarboxylate transport transcriptional regulatory pro	-1,03	NA
PA14_17150	A0A0H2ZES0	Outer membrane protein assembly factor BamA	-1,03	NA
PA14_13680	A0A0H2ZEA2	Putative short-chain dehydrogenase	-1,03	NA
PA14_58550	A0A0H2ZH58	Lipopolysaccharide biosynthetic protein LpxO1	-1,03	NA
PA14_51360	A0A0H2Z7K2	Anthranilate synthase component 1 (EC 4.1.3.27)	-1,03	NA
PA14_08780	Q02T86	DNA-directed RNA polymerase subunit beta' (RNAP subunit beta')	-1,03	NA
PA14_13800	A0A0H2ZF54	Respiratory nitrate reductase beta subuni	-1,03	NA
PA14_15100	A0A0H2ZF58	Putative peptidase	-1,03	NA
PA14_24890	Q02PJ4	Molybdenum cofactor guanylyltransferase (MoCo guanylyltransfe	-1,03	NA
PA14_62590	Q02FU2	Pantothenate synthetase (PS) (EC 6.3.2.1) (Pantoate--beta-alanine	-1,03	NA
PA14_47490	A0A0H2Z8G2	Putative enzyme	-1,03	NA
PA14_10500	A0A0H2ZG25	Cytochrome c oxidase subunit (Cbb3-type)	-1,03	NA
PA14_25400	A0A0H2ZD53	Putative phosphodiesterase	-1,03	NA
PA14_33900	A0A0H2ZA56	Putative aldehyde dehydrogenase	-1,03	NA
PA14_13030	A0A0H2ZFK9	CioA, cyanide insensitive terminal oxidase	-1,03	NA
PA14_72600	A0A0H2ZJT8	Putative lipoprotein	-1,03	NA
PA14_68670	A0A0H2ZIR7	Putative carboxypeptidase	-1,03	NA
PA14_13420	A0A0H2ZEB9	Uncharacterized protein	-1,03	NA
PA14_47460	A0A0H2Z8K1	Ribonuclease D (RNase D) (EC 3.1.13.5)	-1,03	NA
PA14_72840	A0A0H2ZJN2	Putative short chain dehydrogenase	-1,03	NA
PA14_01780	A0A0H2ZK69	Putative nucleoside 2-deoxyribosyltransferase	-1,03	NA
PA14_62990	Q02FR0	Protein GrpE (HSP-70 cofactor)	-1,03	NA
PA14_09090	Q02T57	30S ribosomal protein S11	-1,02	NA
PA14_44300	A0A0H2Z9D2	Aerotaxis receptor Aer	-1,02	NA
PA14_46650	A0A0H2Z8R7	Putative transmembrane sensor	-1,02	NA
PA14_72550	A0A0H2ZIQ9	Putative zinc ABC transporter, periplasmic zinc-binding protein	-1,02	NA
PA14_07070	A0A0H2ZL75	Putative reductase	-1,02	NA
PA14_17270	Q02RB1	Acetyl-coenzyme A carboxylase carboxyl transferase subunit alpha	-1,02	NA
PA14_66580	A0A0H2ZID1	Putative Type II secretory pathway protein	-1,02	NA
PA14_53110	A0A0H2Z774	Probable oxidoreductase	-1,02	NA
PA14_31800	A0A0H2ZAI6	Putative sodium:alanine symporter	-1,02	NA
PA14_62630	A0A0H2ZGT3	Acetyl-coenzyme A synthetase (AcCoA synthetase) (Acs) (EC 6.2.1.	-1,02	NA
PA14_44360	A0A0H2Z804	Cbb3-type cytochrome c oxidase subunit	-1,02	NA
PA14_31680	A0A0H2ZAJ4	Putative outer membrane protein	-1,02	NA
PA14_19870	A0A0H2ZEE5	Leucine dehydrogenase	-1,02	NA
PA14_45620	A0A0H2Z7P6	Two-component response regulator CheY	-1,02	NA
PA14_10730	A0A0H2ZFT2	Putative decarboxylase family protein	-1,02	NA
PA14_47720	A0A0H2Z8I3	Cobalamin biosynthesis protein CobC	-1,02	NA
PA14_66230	A0A0H2ZIJ8	Lipopolysaccharide core biosynthesis protein WaaG	-1,02	NA
PA14_64510	A0A0H2ZH47	Putative exported protein	-1,02	NA
PA14_34600	A0A0H2ZBA0	Putative glyceraldehyde-3-phosphate dehydrogenase	-1,02	NA
PA14_22040	Q02Q74	Probable septum site-determining protein MinC	-1,02	NA
PA14_65250	Q02F79	ATP phosphoribosyltransferase regulatory subunit	-1,02	NA
PA14_71570	A0A0H2ZJL9	Putative aldose 1-epimerase	-1,02	NA
PA14_22020	A0A0H2ZDV1	Site-determining protein	-1,02	NA
PA14_53980	A0A0H2Z720	Uncharacterized protein	-1,02	NA
PA14_24380	A0A0H2ZDA8	Uncharacterized protein	-1,02	NA
PA14_47080	A0A0H2Z8I9	Putative transcriptional regulator	-1,02	NA

PA14_17400	A0A0H2ZDH3	S-(hydroxymethyl)glutathione dehydrogenase (EC 1.1.1.284)	-1,02	NA
PA14_19400	Q02QU5	tRNA 5-methylaminomethyl-2-thiouridine biosynthesis bifunction	-1,02	NA
PA14_51800	Q02IC7	Crossover junction endodeoxyribonuclease RuvC (EC 3.1.22.4) (Hc	-1,02	NA
PA14_72490	A0A0H2ZJK7	DNA polymerase I	-1,02	NA
PA14_67810	A0A0H2ZIK7	Putative carboxyl-terminal protease	-1,02	NA
PA14_55080	A0A0H2Z762	Uncharacterized protein	-1,02	NA
PA14_51320	A0A0H2Z7U6	Putative beta-barrel assembly-enhancing protease (EC 3.4.-.-)	-1,02	NA
PA14_26910	A0A0H2ZCX7	Putative MoxR protein	-1,02	NA
PA14_57450	Q02H20	Ribosomal RNA small subunit methyltransferase H (EC 2.1.1.199) (	-1,02	NA
PA14_57580	Q02H08	30S ribosomal protein S9	-1,02	NA
PA14_51910	A0A0H2Z6B4	Uncharacterized protein	-1,02	NA
PA14_68250	A0A0H2ZIN9	Probable two-component response regulator	-1,02	NA
PA14_47930	A0A0H2Z766	Uncharacterized protein	-1,02	NA
PA14_53250	Q02I11	Chitin-binding protein CbpD	-1,02	NA
PA14_12410	A0A0H2ZFT8	Phosphomethylpyrimidine kinase	-1,02	NA
PA14_49330	A0A0H2Z7Z8	Uncharacterized protein	-1,02	NA
PA14_22570	A0A0H2ZDQ5	CsaA protein	-1,02	NA
PA14_58650	A0A0H2ZGY1	Putative methyl-accepting chemotaxis protein	-1,02	NA
PA14_33280	A0A0H2ZA98	PvdL	-1,02	NA
PA14_57040	A0A0H2ZGV5	Uncharacterized protein	-1,02	NA
PA14_23380	A0A0H2ZDN3	UDP-N-acetyl-D-mannosaminuronate dehydrogenase	-1,02	NA
PA14_51330	Q02IG9	Quinolinate synthase A (EC 2.5.1.72)	-1,02	NA
PA14_40490	A0A0H2Z9N4	Putative membrane protein	-1,02	NA
PA14_64030	A0A0H2ZI98	Uncharacterized protein	-1,02	NA
PA14_04760	Q02U51	Phosphopantetheine adenylyltransferase (EC 2.7.7.3) (Dephosphc	-1,02	NA
PA14_08500	A0A0H2ZLG5	Putative integral membrane protein	-1,02	NA
PA14_51020	A0A0H2Z7N8	Uncharacterized protein	-1,02	NA
PA14_08620	A0A0H2ZGL0	Bifunctional ligase/repressor BirA (Biotin operon repressor) (Bioti	-1,02	NA
PA14_11320	A0A0H2ZG04	Uncharacterized protein	-1,02	NA
PA14_58090	A0A0H2ZGL5	Uncharacterized protein	-1,02	NA
PA14_68900	A0A0H2ZIS4	Putative iron ABC transporter, periplasmic iron-binding protein	-1,02	NA
PA14_63570	A0A0H2ZGY8	Nitrate-inducible formate dehydrogenase, gamma subunit	-1,02	NA
PA14_60700	A0A0H2ZH77	Cytochrome c551 peroxidase	-1,02	NA
PA14_50930	A0A0H2Z7Z0	Uncharacterized protein	-1,02	NA
PA14_46570	A0A0H2Z7H7	Putative transcriptional regulator	-1,02	NA
PA14_51840	A0A0H2Z7Q7	Probable cold-shock protein	-1,02	NA
PA14_16990	A0A0H2ZEV5	Uncharacterized protein	-1,02	NA
PA14_61280	A0A0H2ZHJ6	Putative epimerase	-1,02	NA
PA14_66800	A0A0H2ZIQ0	Uncharacterized protein	-1,02	NA
PA14_50180	A0A0H2Z839	Two-component response regulator	-1,02	NA
PA14_21440	A0A0H2ZE26	Putative HIT family protein	-1,02	NA
PA14_15700	A0A0H2ZF65	Putative amino acid permease	-1,02	NA
PA14_68680	A0A0H2ZHS9	Two-component sensor EnvZ	-1,02	NA
PA14_33010	A0A0H2ZAB7	Serine hydroxymethyltransferase (SHMT) (Serine methylase) (EC 2	-1,02	NA
PA14_68870	A0A0H2ZIT3	Aminomethyltransferase (EC 2.1.2.10) (Glycine cleavage system T	-1,02	NA
PA14_06980	A0A0H2ZKT0	Putative dehydrogenase	-1,02	NA
PA14_30150	Q02NB8	tRNA-specific 2-thiouridylase MnmA (EC 2.8.1.13)	-1,02	NA
PA14_41390	A0A0H2Z8N6	Peptidyl-prolyl cis-trans isomerase (PPIase) (EC 5.2.1.8)	-1,02	NA
PA14_30090	A0A0H2ZC67	Putative acyltransferase	-1,02	NA
PA14_72340	A0A0H2ZIP2	Proton-glutamate symporter	-1,02	NA
PA14_26485	A0A0H2ZLX1	Probable oxidoreductase	-1,02	NA
PA14_16550	A0A0H2ZEQ0	Putative transcriptional regulator, TetR family	-1,02	NA
PA14_60650	A0A0H2ZHF2	RNA-splicing ligase RtcB (EC 6.5.1.-)	-1,02	NA
PA14_13140	A0A0H2ZFA6	Uncharacterized protein	-1,02	NA
PA14_73370	Q02DE3	tRNA uridine 5-carboxymethylaminomethyl modification enzyme	-1,02	NA
PA14_05620	Q02TY0	Adenosylhomocysteinase (EC 3.3.1.1) (S-adenosyl-L-homocysteine	-1,02	NA
PA14_41240	A0A0H2Z9P5	ATP-dependent Clp protease proteolytic subunit (EC 3.4.21.92) (E	-1,02	NA
PA14_53480	A0A0H2Z6Z4	Phosphate acetyltransferase (EC 2.3.1.8) (Phosphotransacetylase)	-1,02	NA
PA14_48750	A0A0H2Z727	Putative redox protein	-1,02	NA

PA14_08950	Q02T70	50S ribosomal protein L14	-1,02	NA
PA14_22930	Q02PZ9	Glucokinase (EC 2.7.1.2) (Glucose kinase)	-1,02	NA
PA14_68170	A0A0H2ZIN4	dTDP-glucose 4,6-dehydratase (EC 4.2.1.46)	-1,02	NA
PA14_57220	Q02H37	Protein translocase subunit SecA	-1,02	NA
PA14_52770	A0A0H2Z7L2	Arginine/ornithine transport protein AotM	-1,02	NA
PA14_51240	Q02IH8	Phosphoribosylaminoimidazole-succinocarboxamide synthase (EC	-1,02	NA
PA14_35440	A0A0H2ZB07	L-asparaginase I	-1,02	NA
PA14_04310	Q02U86	Ribose-5-phosphate isomerase A (EC 5.3.1.6) (Phosphoriboisomer	-1,02	NA
PA14_73420	Q02DD9	Ribonuclease P protein component (RNase P protein) (RNaseP prc	-1,02	NA
PA14_45330	Q02JW1	Cytochrome c-type biogenesis protein CcmE (Cytochrome c matur	-1,02	NA
PA14_15350	A0A0H2ZE01	Putative integrase	-1,02	NA
PA14_52610	A0A0H2Z7M2	Possible threonine aldolase	-1,02	NA
PA14_66970	Q02EU8	Sec-independent protein translocase protein TatB	-1,02	NA
PA14_57190	A0A0H2ZGM7	Putative pyrophosphohydrolase	-1,02	NA
PA14_62190	A0A0H2ZHT0	Uncharacterized protein	-1,02	NA
PA14_62860	A0A0H2ZGU2	ATP-dependent zinc metalloprotease FtsH (EC 3.4.24.-)	-1,02	NA
PA14_14820	Q02RW1	Nucleoside diphosphate kinase (NDK) (NDP kinase) (EC 2.7.4.6) (N	-1,02	NA
PA14_62970	Q02FR1	Chaperone protein DnaK (HSP70) (Heat shock 70 kDa protein) (He	-1,02	NA
PA14_50840	A0A0H2Z7S4	Putative DNA helicase	-1,02	NA
PA14_56720	A0A0H2ZGJ8	Probable dehydrogenase	-1,02	NA
PA14_66200	A0A0H2ZHE7	Putative heptose kinase WapQ	-1,02	NA
PA14_51930	A0A0H2Z761	Probable thioredoxin	-1,02	NA
PA14_25630	Q02PD3	50S ribosomal protein L32	-1,02	NA
PA14_41250	Q02KU3	Trigger factor (TF) (EC 5.2.1.8) (PPIase)	-1,02	NA
PA14_73280	Q02DF1	ATP synthase subunit delta (ATP synthase F(1) sector subunit delt	-1,02	NA
PA14_63540	Q02FL3	L-seryl-tRNA(Sec) selenium transferase (EC 2.9.1.1) (Selenocystein	-1,02	NA
PA14_66480	A0A0H2ZIM6	Putative ATPase nvolved in chromosome partitioning	-1,02	NA
PA14_68190	A0A0H2ZHP4	dTDP-4-dehydrorhamnose reductase	-1,02	NA
PA14_68820	A0A0H2ZIS8	Putative general secretory pathway related protein	-1,02	NA
PA14_16600	A0A0H2ZF10	Putative hydrolase, alpha/beta family	-1,02	NA
PA14_12300	A0A0H2ZFG3	Putative Mg2+ and Co2+ transporter CorC	-1,02	NA
PA14_71780	A0A0H2ZJM4	Putative transcriptional regulator, RpiR family	-1,02	NA
PA14_57390	A0A0H2ZGN5	UDP-N-acetylmuramoyl-tripeptide--D-alanyl-D-alanine ligase (EC 6	-1,02	NA
PA14_60750	A0A0H2ZHF1	Putative activator of alkane oxidation	-1,02	NA
PA14_37820	A0A0H2ZAB3	Uncharacterized protein	-1,02	NA
PA14_66720	A0A0H2ZIE1	Primosomal protein N' (EC 3.6.4.-) (ATP-dependent helicase PriA)	-1,02	NA
PA14_01130	A0A0H2ZKA1	Uncharacterized protein	-1,01	NA
PA14_52760	A0A0H2Z669	Uncharacterized protein	-1,01	NA
PA14_18590	Q02R10	UPF0246 protein PA14_18590	-1,01	NA
PA14_16050	A0A0H2ZEZ9	Thiol:disulfide interchange protein DsbC	-1,01	NA
PA14_33310	Q02MM1	CRISPR-associated protein Csy3	-1,01	NA
PA14_08790	Q02T85	30S ribosomal protein S12	-1,01	NA
PA14_50220	A0A0H2Z7X4	Transcriptional regulator FleQ	-1,01	NA
PA14_17290	Q02RA9	CTP synthase (EC 6.3.4.2) (Cytidine 5'-triphosphate synthase) (Cyt	-1,01	NA
PA14_12230	Q02SF5	Leucine--tRNA ligase (EC 6.1.1.4) (Leucyl-tRNA synthetase) (LeuRS	-1,01	NA
PA14_70420	Q02E28	Ribonuclease PH (RNase PH) (EC 2.7.7.56) (tRNA nucleotidyltransf	-1,01	NA
PA14_56060	A0A0H2ZG95	Formyltetrahydrofolate deformylase (EC 3.5.1.10) (Formyl-FH(4) h	-1,01	NA
PA14_07190	Q02TL3	Phosphoglycerate kinase (EC 2.7.2.3)	-1,01	NA
PA14_31540	A0A0H2ZBW4	Putative acyl-CoA dehydrogenase	-1,01	NA
PA14_27730	A0A0H2ZCS3	Putative acyl-CoA dehydrogenase	-1,01	NA
PA14_38430	A0A0H2Z961	Regulatory gene of gnyRDBHAL cluster, GnyR	-1,01	NA
PA14_28260	A0A0H2ZCB7	Uncharacterized protein	-1,01	NA
PA14_64520	A0A0H2ZHY7	Putative bacterioferritin	-1,01	NA
PA14_48090	A0A0H2Z8K0	Alkaline protease secretion outer membrane protein AprF	-1,01	NA
PA14_58170	Q02GV9	Aspartyl/glutamyl-tRNA(Asn/Gln) amidotransferase subunit C (As	-1,01	NA
PA14_04040	A0A0H2ZKN8	Uncharacterized protein	-1,01	NA
PA14_35830	A0A0H2Z9R3	Cointegrate resolution protein T	-1,01	NA
PA14_17940	A0A0H2ZDE0	Glycerol-3-phosphate regulon repressor	-1,01	NA
PA14_16890	A0A0H2ZDK0	Putative auxiliary component of ABC transporter	-1,01	NA

PA14_58350	A0A0H2ZGV2	Putative binding protein component of ABC transpor	-1,01	NA
PA14_52190	Q02I93	23S rRNA (uracil(1939)-C(5))-methyltransferase RlmD (EC 2.1.1.19	-1,01	NA
PA14_23500	A0A0H2ZDC3	Aminotransferase (EC 2.6.1.-)	-1,01	NA
PA14_52370	A0A0H2Z7C8	Uncharacterized protein	-1,01	NA
PA14_19950	A0A0H2ZE07	Uncharacterized protein	-1,01	NA
PA14_23860	Q02PS5	Acetyl-coenzyme A carboxylase carboxyl transferase subunit beta	-1,01	NA
PA14_13230	Q02S84	Cyclic pyranopterin monophosphate synthase accessory protein (	-1,01	NA
PA14_41670	Q02KR1	Phosphoenolpyruvate synthase (PEP synthase) (EC 2.7.9.2) (Pyruv	-1,01	NA
PA14_30770	A0A0H2ZAT5	Putative transcriptional regulator, AsnC family	-1,01	NA
PA14_05820	A0A0H2ZK81	Putative acyl-CoA transferase	-1,01	NA
PA14_11450	A0A0H2ZFV5	N utilization substance protein B homolog (Protein NusB)	-1,01	NA
PA14_10700	A0A0H2ZG02	Putative bacteriophytochrome	-1,01	NA
PA14_42010	A0A0H2Z9J7	Putative glutamine amidotransferase	-1,01	NA
PA14_58375	A0A0H2ZM75	Probable metallopeptidase	-1,01	NA
PA14_23260	A0A0H2ZCA6	DNA gyrase subunit A (EC 5.99.1.3)	-1,01	NA
PA14_45020	A0A0H2Z8Y7	Putative oxidoreductase	-1,01	NA
PA14_19450	Q02QU2	UPF0502 protein PA14_19450	-1,01	NA
PA14_60250	A0A0H2ZHB7	Kinase sensor protein of two component regulatory	-1,01	NA
PA14_00280	Q02V57	Oxygen-dependent coproporphyrinogen-III oxidase (CPO) (Coprog	-1,01	NA
PA14_24060	A0A0H2ZC21	General secretion pathway protein J	-1,01	NA
PA14_19210	A0A0H2ZEE1	Putative dihydrofolate reductase	-1,01	NA
PA14_43130	A0A0H2Z927	Putative aminotransferase	-1,01	NA
PA14_71650	A0A0H2ZJ98	Aspartate ammonia-lyase (Aspartase) (EC 4.3.1.1)	-1,01	NA
PA14_70580	A0A0H2ZJG3	Putative signal transduction protein	-1,01	NA
PA14_23080	A0A0H2ZDE4	6-phosphogluconolactonase	-1,01	NA
PA14_30230	A0A0H2ZAW8	ATP-dependent clp protease, ATP-binding subunit ClpA	-1,01	NA
PA14_28580	A0A0H2ZCI7	Uncharacterized protein	-1,01	NA
PA14_15980	Q02RL7	Ribosome maturation factor RimM	-1,01	NA
PA14_66310	A0A0H2ZID8	Acetyltransferase component of pyruvate dehydrogenase comple	-1,01	NA
PA14_25900	Q02PB3	Enoyl-[acyl-carrier-protein] reductase [NADH] (ENR) (EC 1.3.1.9)	-1,01	NA
PA14_05390	A0A0H2ZL32	ChpA	-1,01	NA
PA14_57920	A0A0H2ZGK2	Lipopolysaccharide export system protein LptA	-1,01	NA
PA14_21310	A0A0H2ZDX9	(R)-specific enoyl-CoA hydratase	-1,01	NA
PA14_23790	A0A0H2ZDG4	3-isopropylmalate dehydrogenase (EC 1.1.1.85) (3-IPM-DH) (Beta-	-1,01	NA
PA14_27980	A0A0H2ZCN5	tRNA-dihydrouridine(20/20a) synthase (EC 1.3.1.91) (U20-specific	-1,01	NA
PA14_41930	A0A0H2Z9C7	Putative acetyltransferase	-1,01	NA
PA14_72520	A0A0H2ZJT2	Uncharacterized protein	-1,01	NA
PA14_66170	A0A0H2ZIJ0	Putative carbamoyltransferase	-1,01	NA
PA14_48710	A0A0H2Z7X5	Uncharacterized protein	-1,01	NA
PA14_04460	Q02U74	Prolipoprotein diacylglyceryl transferase (EC 2.4.99.-)	-1,01	NA
PA14_14880	Q02RV7	4-hydroxy-3-methylbut-2-en-1-yl diphosphate synthase (flavodox	-1,01	NA
PA14_70810	A0A0H2ZIB3	Phosphate import ATP-binding protein PstB (EC 3.6.3.27) (ABC ph	-1,01	NA
PA14_44980	A0A0H2Z7T7	Putative transcriptional regulator, TetR family	-1,01	NA
PA14_30450	A0A0H2ZC49	Putative transcriptional regulator, LysR family	-1,01	NA
PA14_71080	A0A0H2ZJ82	Uncharacterized protein	-1,01	NA
PA14_20730	A0A0H2ZE25	Putative negative regulator of flagellin synthesis, FlgM	-1,01	NA
PA14_61890	A0A0H2ZHN6	Uncharacterized protein	-1,01	NA
PA14_44770	A0A0H2Z7V3	Probable guanine deaminase	-1,01	NA
PA14_47120	A0A0H2Z8M6	Uncharacterized protein	-1,01	NA
PA14_38330	A0A0H2ZA70	Glutathione reductase	-1,01	NA
PA14_14890	Q02RV6	Histidine--tRNA ligase (EC 6.1.1.21) (Histidyl-tRNA synthetase) (His	-1,01	NA
PA14_49250	Q02IZ4	Periplasmic nitrate reductase (EC 1.7.99.4)	-1,01	NA
PA14_40560	A0A0H2Z9M7	Uncharacterized protein	-1,01	NA
PA14_61990	A0A0H2ZHJ7	Uncharacterized protein	-1,01	NA
PA14_44030	A0A0H2Z823	Succinate dehydrogenase flavoprotein subunit (EC 1.3.5.1)	-1,01	NA
PA14_57900	A0A0H2ZGT0	Putative phophatase	-1,01	NA
PA14_23250	Q02PX5	Methylthioribose-1-phosphate isomerase (M1Pi) (MTR-1-P isome	-1,01	NA
PA14_45580	A0A0H2Z912	Chemotaxis response regulator protein-glutamate methylesterase	-1,01	NA
PA14_14910	A0A0H2ZF77	Outer membrane protein assembly factor BamB	-1,01	NA

PA14_01760	A0A0H2ZJ79	Nonspecific ribonucleoside hydrolase	-1,01	NA
PA14_64930	A0A0H2ZI60	Putative ADP-ribose pyrophosphatase	-1,01	NA
PA14_20650	Q02QJ1	Probable D-serine dehydratase (EC 4.3.1.18) (D-serine deaminase	-1,01	NA
PA14_16510	A0A0H2ZF15	Peptide chain release factor 2 (RF-2)	-1,01	NA
PA14_51260	A0A0H2Z6G9	Putative lipoprotein	-1,01	NA
PA14_00070	A0A0H2ZK05	D,D-heptose 1,7-bisphosphate phosphatase (EC 3.1.3.-)	-1,01	NA
PA14_42760	Q02KH1	Chorismate synthase (CS) (EC 4.2.3.5) (5-enolpyruvylshikimate-3-p	-1,01	NA
PA14_57480	A0A0H2ZGY2	Putative lipoprotein	-1,01	NA
PA14_07650	A0A0H2ZLS6	Putative sporulation protein	-1,01	NA
PA14_50970	A0A0H2Z7P9	Probable glutathione S-transferase	-1,01	NA
PA14_01600	A0A0H2ZJV2	Putative aldehyde dehydrogenase	-1,01	NA
PA14_43290	A0A0H2Z890	Putative lipoprotein	-1,01	NA
PA14_16580	A0A0H2ZEX5	Uncharacterized protein	-1,01	NA
PA14_27330	A0A0H2ZCV0	Phospho-2-dehydro-3-deoxyheptonate aldolase (EC 2.5.1.54)	-1,01	NA
PA14_17080	Q02RC6	Uridylate kinase (UK) (EC 2.7.4.22) (Uridine monophosphate kinas	-1,01	NA
PA14_30200	A0A0H2ZC07	Cold-shock protein CspD	-1,01	NA
PA14_21540	A0A0H2ZDW4	Putative 3-oxoacyl-(Acyl-carrier-protein) synthase III	-1,01	NA
PA14_30240	Q02NB1	Translation initiation factor IF-1	-1,01	NA
PA14_56850	A0A0H2ZGK8	Putative lipoprotein	-1,01	NA
PA14_51880	A0A0H2Z7G7	Basic amino acid, basic peptide and imipenem outer membrane p	-1,01	NA
PA14_35500	A0A0H2ZB21	Dihydrolipoamide acetyltransferase component of pyruvate dehy	-1,01	NA
PA14_12400	Q02SE4	Thiamine-phosphate synthase (TP synthase) (TPS) (EC 2.5.1.3) (Th	-1,01	NA
PA14_71670	A0A0H2ZIH5	Putative membrane protein	-1,01	NA
PA14_67410	A0A0H2ZHK4	Uncharacterized protein	-1,01	NA
PA14_51900	Q02IB8	Proline--tRNA ligase (EC 6.1.1.15) (Prolyl-tRNA synthetase) (ProRS	-1,01	NA
PA14_14440	A0A0H2ZF14	Valine--tRNA ligase (EC 6.1.1.9) (Valyl-tRNA synthetase)	-1,01	NA
PA14_07170	A0A0H2ZL79	D-erythrose-4-phosphate dehydrogenase (EC 1.2.1.72)	-1,01	NA
PA14_56660	A0A0H2ZGJ3	Probable xenobiotic reductase B	-1,01	NA
PA14_65010	A0A0H2ZH82	Cytokinin riboside 5'-monophosphate phosphoribohydrolase (EC :	-1,01	NA
PA14_05510	Q02TY9	UPF0312 protein PA14_05510	-1,01	NA
PA14_15120	A0A0H2ZEY0	Putative membrane or periplasmic protein	-1,01	NA
PA14_25040	A0A0H2ZD86	Universal stress protein	-1,01	NA
PA14_60290	A0A0H2ZHD1	Type 4 fimbrial biogenesis protein PilW	-1,01	NA
PA14_65740	Q02F45	Phosphomethylpyrimidine synthase (EC 4.1.99.17) (Hydroxymethy	-1,01	NA
PA14_45970	A0A0H2Z8C8	Putative cation-transporting P-type ATPase	-1,01	NA
PA14_15480	A0A0H2ZEV9	Regulatory protein merR	-1,01	NA
PA14_08960	Q02T69	50S ribosomal protein L24	-1,01	NA
PA14_29990	Q02ND1	NADH-quinone oxidoreductase subunit C/D (EC 1.6.5.11) (NADH c	-1,01	NA
PA14_53220	A0A0H2Z7H8	Fumarate hydratase class II (Fumarase C) (EC 4.2.1.2)	-1,01	NA
PA14_62880	A0A0H2ZHN7	Putative RNA-binding protein	-1,01	NA
PA14_72640	A0A0H2ZIR4	Putative ABC transporter, periplasmic binding protein	-1,01	NA
PA14_41380	Q02KT5	Glutamine--tRNA ligase (EC 6.1.1.18) (Glutaminyl-tRNA synthetase	-1,01	NA
PA14_52980	A0A0H2Z785	Transcriptional regulator PhhR	-1,00	NA
PA14_70560	A0A0H2ZI92	Putative transcriptional regulator, LysR family	-1,00	NA
PA14_30630	Q02N79	2-heptyl-3-hydroxy-4(1H)-quinolone synthase (EC 1.14.13.182) (2-	-1,00	NA
PA14_31650	A0A0H2ZBU4	Exodeoxyribonuclease III	-1,00	NA
PA14_12010	Q02SH4	Gamma-glutamyl phosphate reductase (GPR) (EC 1.2.1.41) (Glutai	-1,00	NA
PA14_54390	A0A0H2Z7B2	Serine protease MucD	-1,00	NA
PA14_53330	Q02I05	Sulfate transporter CysZ	-1,00	NA
PA14_47500	A0A0H2Z797	3-mercaptopyruvate sulfurtransferase	-1,00	NA
PA14_07910	A0A0H2ZKY8	Ribulose-phosphate 3-epimerase (EC 5.1.3.1)	-1,00	NA
PA14_01560	A0A0H2ZK57	Putative alkylphosphonate uptake protein	-1,00	NA
PA14_58780	A0A0H2ZFX2	Dephospho-CoA kinase (EC 2.7.1.24) (Dephosphocoenzyme A kina	-1,00	NA
PA14_26460	A0A0H2ZBN8	Precorrin-6x reductase	-1,00	NA
PA14_12330	A0A0H2ZEJ1	PhoH family protein	-1,00	NA
PA14_54500	A0A0H2Z7A5	Probable two-component sensor	-1,00	NA
PA14_56160	A0A0H2ZGH4	Putative methanol dehydrogenase regulatory protein	-1,00	NA
PA14_70050	A0A0H2ZJ22	Uncharacterized protein	-1,00	NA
PA14_52150	A0A0H2Z7E1	Lipopolysaccharide biosynthetic protein LpxO2	-1,00	NA

PA14_67420	A0A0H2ZII8	Histidine utilization genes repressor protein	-1,00	NA
PA14_16690	A0A0H2ZEW7	Phosphoenolpyruvate carboxylase (PEPC) (PEPCase) (EC 4.1.1.31)	-1,00	NA
PA14_66250	A0A0H2ZHE9	Heptosyltransferase II	-1,00	NA
PA14_60320	A0A0H2ZGA0	Type 4 fimbrial biogenesis protein PilE	-1,00	NA
PA14_09050	A0A0H2ZGC8	Protein translocase subunit SecY	-1,00	NA
PA14_09400	A0A0H2ZGC1	Flavin-containing monooxygenase	-1,00	NA
PA14_16840	A0A0H2ZES8	Putative lipoprotein	-1,00	NA
PA14_14230	A0A0H2ZFG4	Putative periplasmic transport protein	-1,00	NA
PA14_45610	Q02JU1	Protein phosphatase CheZ (EC 3.1.3.-) (Chemotaxis protein CheZ)	-1,00	NA
PA14_16430	A0A0H2ZEY3	Putative methyl-accepting chemotaxis transducer	-1,00	NA
PA14_70400	A0A0H2ZJ50	Uncharacterized protein	-1,00	NA
PA14_44690	A0A0H2Z7V8	Putative transcriptional regulator, GntR family	-1,00	NA
PA14_57340	Q02H28	UDP-N-acetylglucosamine--N-acetylmuramyl-(pentapeptide) pyro	-1,00	NA
PA14_65560	A0A0H2ZI51	Phosphoserine phosphatase	-1,00	NA
PA14_49850	A0A0H2Z6W0	Uncharacterized protein	-1,00	NA
PA14_15470	A0A0H2ZF85	Periplasmic mercury ion-binding protein	-1,00	NA
PA14_17320	Q02RA7	Enolase (EC 4.2.1.11) (2-phospho-D-glycerate hydro-lyase) (2-pho	-1,00	NA
PA14_27000	A0A0H2ZCL3	Putative methyl-accepting chemotaxis protein	-1,00	NA
PA14_66140	A0A0H2ZHE2	Uncharacterized protein	-1,00	NA
PA14_57980	A0A0H2ZGK7	Putative phosphoryl carrier protein	-1,00	NA
PA14_00820	A0A0H2ZJZ5	Uncharacterized protein	-1,00	NA
PA14_11270	A0A0H2ZFN3	Outer membrane protein OprG	-1,00	NA
PA14_42690	A0A0H2Z9E1	Polyamine aminopropyltransferase (EC 2.5.1.16) (Putrescine amin	-1,00	NA
PA14_63170	A0A0H2ZHX9	Putative transcriptional regulator	-1,00	NA
PA14_50800	Q02IL5	Pyridoxine/pyridoxamine 5'-phosphate oxidase (EC 1.4.3.5) (PNP/	-1,00	NA
PA14_29590	A0A0H2ZC98	Putative transcriptional regulator	-1,00	NA
PA14_64980	Q02F98	NH(3)-dependent NAD(+) synthetase (EC 6.3.1.5)	-1,00	NA
PA14_09115	Q02T55	DNA-directed RNA polymerase subunit alpha (RNAP subunit alpha	-1,00	NA
PA14_31530	A0A0H2ZAK2	Putative acyl-CoA thiolase	-1,00	NA
PA14_44380	A0A0H2Z8T6	Putative cytochrome c oxidase, cbb3-type, subunit II	-1,00	NA
PA14_04090	A0A0H2ZKP3	Putative binding protein component of ABC transporter	-1,00	NA
PA14_57680	A0A0H2ZGR3	Putative hydrolase	-1,00	NA
PA14_19360	A0A0H2ZD61	Putative acetyltransferase, GNAT family	-1,00	NA
PA14_40710	A0A0H2Z9Z9	Uncharacterized protein	1,00	NA
PA14_25440	A0A0H2ZD68	Lipoprotein-releasing system ATP-binding protein LolD (EC 3.6.3.-	1,00	NA
PA14_69630	A0A0H2ZJ97	Nucleoside diphosphate kinase regulator	1,00	NA
PA14_20720	A0A0H2ZE48	Putative export chaperone involved in flagellar synthesis	1,00	NA
PA14_19010	A0A0H2ZEH3	Uncharacterized protein	1,00	NA
PA14_47420	A0A0H2Z7A2	Uncharacterized protein	1,00	NA
PA14_44020	A0A0H2Z979	Succinate dehydrogenase (B subunit)	1,00	NA
PA14_30550	A0A0H2ZAU9	Putative periplasmic aliphatic sulfonate-binding protein	1,00	NA
PA14_23590	A0A0H2ZDB8	Putative transcriptional regulator	1,00	NA
PA14_57950	A0A0H2ZGT5	Putative ribosomal subunit interface protein	1,00	NA
PA14_73310	Q02DE8	ATP synthase subunit a (ATP synthase F0 sector subunit a) (F-ATPa	1,00	NA
PA14_69070	A0A0H2ZJ45	Putative ATP-binding/permease fusion ABC transporter	1,00	NA
PA14_52600	Q02I63	Alanine--tRNA ligase (EC 6.1.1.7) (Alanyl-tRNA synthetase) (AlaRS)	1,00	NA
PA14_72000	A0A0H2ZIL5	Oxidoreductase Rmd	1,00	NA
PA14_62620	Q02FU0	Glucose-6-phosphate isomerase (GPI) (EC 5.3.1.9) (Phosphoglucos	1,00	NA
PA14_25590	A0A0H2ZD59	Putative hydrolase	1,00	NA
PA14_61460	A0A0H2ZHJ4	Putative hypoxanthine phosphoribosyltransferase	1,00	NA
PA14_11890	A0A0H2ZFS5	Uncharacterized protein	1,00	NA
PA14_15970	Q02RL8	30S ribosomal protein S16	1,00	NA
PA14_70720	Q02E05	Probable chorismate pyruvate-lyase (CL) (CPL) (EC 4.1.3.40)	1,00	NA
PA14_16970	A0A0H2ZET0	Putative arsenate reductase, glutaredoxin family	1,00	NA
PA14_44010	A0A0H2Z9D0	2-oxoglutarate dehydrogenase E1 subunit	1,00	NA
PA14_02260	A0A0H2ZJB8	Putative two-component response regulator	1,00	NA
PA14_01020	A0A0H2ZJR5	Uncharacterized protein	1,00	NA
PA14_21030	A0A0H2ZDR8	ATP-dependent Clp protease proteolytic subunit (EC 3.4.21.92) (E	1,00	NA
PA14_65420	Q02F66	Putative ribosome biogenesis GTPase RsgA (EC 3.6.1.-)	1,00	NA

PA14_44430	A0A0H2Z9C4	Uncharacterized protein	1,00	NA
PA14_70590	A0A0H2ZJF0	Uncharacterized protein	1,00	NA
PA14_22710	A0A0H2ZDF8	Putative nitroreductase family protein	1,00	NA
PA14_14080	A0A0H2ZFH5	Carboxylesterase	1,00	NA
PA14_68860	A0A0H2ZJ30	Glycine cleavage system H protein	1,00	NA
PA14_19660	A0A0H2ZEB2	Putative flavodoxin	1,00	NA
PA14_57590	Q02H07	50S ribosomal protein L13	1,00	NA
PA14_25860	A0A0H2ZBS5	Electron transfer flavoprotein beta-subunit	1,00	NA
PA14_56090	A0A0H2ZGG9	Uncharacterized protein	1,00	NA
PA14_38640	A0A0H2Z946	Putative CoA transferase, subunit B	1,00	NA
PA14_71970	A0A0H2ZJN9	GDP-mannose pyrophosphorylase	1,00	NA
PA14_62560	A0A0H2ZHV5	Poly(A) polymerase I (PAP I) (EC 2.7.7.19)	1,00	NA
PA14_05270	Q02U09	Bifunctional protein PyrR [Includes: Pyrimidine operon regulatory	1,00	NA
PA14_21020	A0A0H2ZE56	Putative non-ribosomal peptide synthetase	1,00	NA
PA14_38610	A0A0H2ZAD8	Putative short-chain fatty acid transporter	1,00	NA
PA14_53970	A0A0H2Z6W1	Probable aconitate hydratase	1,00	NA
PA14_69280	Q02EB7	UPF0178 protein PA14_69280	1,00	NA
PA14_72050	A0A0H2ZIL9	Uncharacterized protein	1,00	NA
PA14_14270	A0A0H2ZE65	Putative hydrolase, isochorismatase family	1,00	NA
PA14_38690	A0A0H2ZAD4	Putative AMP-(Fatty) acid ligase	1,00	NA
PA14_70860	Q02DZ3	Phosphate-binding protein PstS	1,00	NA
PA14_00100	Q02V72	Glycine--tRNA ligase alpha subunit (EC 6.1.1.14) (Glycyl-tRNA synt	1,00	NA
PA14_21890	A0A0H2ZDW2	Putative quinone oxidoreductase	1,00	NA
PA14_14460	A0A0H2ZFA3	DNA polymerase holoenzyme chi subunit	1,00	NA
PA14_45790	A0A0H2Z8E6	Flagellar motor switch protein FliN	1,00	NA
PA14_01980	A0A0H2ZK95	Putative transcriptional regulator, LysR family	1,00	NA
PA14_52580	A0A0H2Z678	Aspartokinase (EC 2.7.2.4)	1,00	NA
PA14_05050	A0A0H2ZKH4	Non-canonical purine NTP pyrophosphatase (EC 3.6.1.19) (Non-st	1,01	NA
PA14_64230	A0A0H2ZI10	RetS (Regulator of Exopolysaccharide and Type III Secretion)	1,01	NA
PA14_07560	Q02TI4	30S ribosomal protein S21	1,01	NA
PA14_67270	A0A0H2ZIT1	Putative ATP-binding component of ABC transporter	1,01	NA
PA14_53070	A0A0H2Z633	4-hydroxyphenylpyruvate dioxygenase	1,01	NA
PA14_41430	A0A0H2Z9G6	Putative tRNA-(Ms(2)io(6)a)-hydroxylase	1,01	NA
PA14_23680	A0A0H2ZDI9	Putative small heat shock protein	1,01	NA
PA14_05530	A0A0H2ZKX6	RND multidrug efflux membrane fusion protein MexA	1,01	NA
PA14_61720	A0A0H2ZHF6	Uncharacterized protein	1,01	NA
PA14_09010	Q02T64	50S ribosomal protein L18	1,01	NA
PA14_03450	A0A0H2ZKQ4	4-aminobutyrate aminotransferase	1,01	NA
PA14_21370	A0A0H2ZCP3	Long-chain-fatty-acid--CoA ligase	1,01	NA
PA14_01710	Q02UU0	Alkyl hydroperoxide reductase subunit C (EC 1.11.1.15) (Peroxired	1,01	NA
PA14_58700	A0A0H2ZGX8	Nicotinate-nucleotide pyrophosphorylase	1,01	NA
PA14_63250	A0A0H2ZGW6	Putative acyl-CoA thiolase	1,01	NA
PA14_41160	A0A0H2Z9Q0	Putative ATP-binding component of ABC transporter	1,01	NA
PA14_26560	A0A0H2ZCY0	Putative outer membrane protein	1,01	NA
PA14_41350	Q02KT7	Bifunctional protein FolD [Includes: Methylenetetrahydrofolate de	1,01	NA
PA14_20600	A0A0H2ZE34	Uncharacterized protein	1,01	NA
PA14_31810	A0A0H2ZBU9	Probable thiol peroxidase (EC 1.11.1.-)	1,01	NA
PA14_53590	A0A0H2Z750	Uncharacterized protein	1,01	NA
PA14_64050	A0A0H2ZI38	Putative two-component response regulator	1,01	NA
PA14_68530	A0A0H2ZJ03	Putative 3-hydroxyacyl-CoA dehydrogenase, NAD binding domain	1,01	NA
PA14_57320	A0A0H2ZFL3	D-alanine--D-alanine ligase (EC 6.3.2.4) (D-Ala-D-Ala ligase) (D-ala	1,01	NA
PA14_01550	A0A0H2ZK60	Putative lipoprotein	1,01	NA
PA14_63270	A0A0H2ZHQ9	Putative short-chain dehydrogenase	1,01	NA
PA14_40890	A0A0H2ZA18	Putative short-chain dehydrogenase	1,01	NA
PA14_00180	A0A0H2ZJY3	Ribosomal RNA small subunit methyltransferase B (EC 2.1.1.-)	1,01	NA
PA14_62940	Q02FR3	4-hydroxy-tetrahydrodipicolinate reductase (HTPA reductase) (EC	1,01	NA
PA14_09070	Q02T59	50S ribosomal protein L36 1	1,01	NA
PA14_51270	A0A0H2Z7V1	4-hydroxy-tetrahydrodipicolinate synthase (HTPA synthase) (EC 4.	1,01	NA
PA14_57360	A0A0H2ZGG8	Putative lipid II flippase FtsW (Cell division protein FtsW)	1,01	NA

PA14_25540	A0A0H2ZD43	Phosphotyrosine protein phosphatase	1,01	NA
PA14_00250	A0A0H2ZJU1	Quinone oxidoreductase	1,01	NA
PA14_14710	A0A0H2ZFC9	Putative Rrf2 family protein	1,01	NA
PA14_02450	A0A0H2ZKK8	Putative NAD(P) transhydrogenase, subunit alpha part 1	1,01	NA
PA14_72540	A0A0H2ZJF7	Putative ribonucleotide reductase	1,01	NA
PA14_09580	A0A0H2ZGA8	Uncharacterized protein	1,01	NA
PA14_05950	A0A0H2ZKL6	RNA helicase DbpA	1,01	NA
PA14_51790	Q02IC8	Holliday junction ATP-dependent DNA helicase RuvA (EC 3.6.4.12)	1,01	NA
PA14_17990	A0A0H2ZEQ7	Cys-tRNA(Pro)/Cys-tRNA(Cys) deacylase (EC 4.2.-.-)	1,01	NA
PA14_50630	A0A0H2Z7S2	Uncharacterized protein	1,01	NA
PA14_57275	A0A0H2ZM25	Cell division protein FtsZ	1,01	NA
PA14_23560	Q02PU7	Glutamate--tRNA ligase (EC 6.1.1.17) (Glutamyl-tRNA synthetase)	1,01	NA
PA14_12370	A0A0H2ZFP1	Uncharacterized protein	1,01	NA
PA14_32420	A0A0H2ZBQ3	Putative Zn-dependent oxidoreductase	1,01	NA
PA14_23340	Q02PW7	Integration host factor subunit beta (IHF-beta)	1,01	NA
PA14_44160	A0A0H2Z9E0	NAD kinase (EC 2.7.1.-) (EC 2.7.1.23)	1,01	NA
PA14_14900	A0A0H2ZE27	Uncharacterized protein	1,01	NA
PA14_17130	Q02RC2	1-deoxy-D-xylulose 5-phosphate reductoisomerase (DXP reductoi	1,01	NA
PA14_25730	A0A0H2ZBT3	Putative aminodeoxychorismate lyase	1,01	NA
PA14_63880	A0A0H2ZHU9	Putative transcriptional regulator	1,01	NA
PA14_24640	Q02PL5	Dihydroorotate dehydrogenase (quinone) (EC 1.3.5.2) (DHOdehas	1,01	NA
PA14_11420	A0A0H2ZEN7	3,4-dihydroxy-2-butanone 4-phosphate synthase (DHBP synthase	1,01	NA
PA14_65160	A0A0H2ZI75	Putative membrane protein	1,01	NA
PA14_34500	A0A0H2ZA13	Putative ATP-binding component of ABC transporter	1,01	NA
PA14_25840	A0A0H2ZCU5	Putative electron transfer flavoprotein-ubiquinone oxidoreductas	1,01	NA
PA14_39070	A0A0H2ZAE3	Uncharacterized protein	1,01	NA
PA14_61390	A0A0H2ZHK6	Putative lipoprotein	1,01	NA
PA14_69980	A0A0H2ZI56	Putative transcriptional regulator	1,01	NA
PA14_72390	A0A0H2ZJE8	Putative two-component sensor	1,01	NA
PA14_30100	A0A0H2ZC08	Uncharacterized protein	1,01	NA
PA14_60445	Q02GB1	GTPase Obg (EC 3.6.5.-) (GTP-binding protein Obg)	1,01	NA
PA14_53300	A0A0H2Z7H4	Probable alkyl hydroperoxide reductase	1,01	NA
PA14_67520	A0A0H2ZIU9	Uncharacterized protein	1,01	NA
PA14_43380	A0A0H2Z885	Potassium-transporting ATPase ATP-binding subunit (EC 3.6.3.12)	1,01	NA
PA14_62150	A0A0H2ZHP5	Acetolactate synthase isozyme III small subunit	1,01	NA
PA14_57460	Q02H19	Transcriptional regulator MraZ	1,01	NA
PA14_29460	A0A0H2ZCD3	Putative quinone oxidoreductase	1,01	NA
PA14_23920	A0A0H2ZC62	Amidophosphoribosyltransferase (ATase) (EC 2.4.2.14) (Glutamin	1,01	NA
PA14_03930	A0A0H2ZL00	Polyamine transport protein	1,01	NA
PA14_06640	A0A0H2ZKD5	Putative acyl-CoA dehydrogenase	1,01	NA
PA14_18690	A0A0H2ZE94	Putative alkyl hydroperoxide reductase subunit	1,01	NA
PA14_70800	A0A0H2ZJ65	Phosphate-specific transport system accessory protein PhoU	1,01	NA
PA14_55810	A0A0H2ZGE3	Putative two-component response regulator	1,01	NA
PA14_43270	Q02KC8	tRNA 2-selenouridine synthase (EC 2.9.1.-) (Selenophosphate-dep	1,01	NA
PA14_43310	A0A0H2Z914	Uncharacterized protein	1,01	NA
PA14_40960	A0A0H2ZA14	Pilin biosynthetic protein	1,01	NA
PA14_38470	A0A0H2ZAE6	Putative enoyl-CoA hydratase	1,01	NA
PA14_08520	A0A0H2ZM02	Anhydro-N-acetylmuramic acid kinase (EC 2.7.1.170) (AnhMurNA	1,01	NA
PA14_44470	A0A0H2Z7Z6	Coproporphyrinogen-III oxidase (EC 1.3.99.22)	1,01	NA
PA14_69200	A0A0H2ZIU3	Thioredoxin	1,01	NA
PA14_35760	A0A0H2ZAY1	Uncharacterized protein	1,01	NA
PA14_03710	A0A0H2ZK99	Uncharacterized protein	1,01	NA
PA14_04650	A0A0H2ZKF3	Protease PfpI	1,01	NA
PA14_42080	A0A0H2Z9J2	Putative 3-hydroxyacyl-CoA dehydrogenase	1,01	NA
PA14_24430	A0A0H2ZDA3	Putative MoxR-like ATPase	1,01	NA
PA14_33330	Q02ML9	CRISPR-associated protein Csy1	1,01	NA
PA14_07550	A0A0H2ZKW8	Uncharacterized protein	1,01	NA
PA14_72870	A0A0H2ZJW3	Putative glutamate-1-semialdehyde aminotransferase	1,01	NA
PA14_28690	Q02NN7	Phenylalanine--tRNA ligase alpha subunit (EC 6.1.1.20) (Phenylala	1,01	NA

PA14_72360	A0A0H2ZJR7	Uncharacterized protein	1,01	NA
PA14_14040	Q02S21	ATP-dependent RNA helicase RhlB (EC 3.6.4.13)	1,01	NA
PA14_26550	A0A0H2ZCZ9	Putative lipoprotein	1,01	NA
PA14_06530	A0A0H2ZKD0	Putative biotin biosynthesis protein bioH	1,01	NA
PA14_24990	A0A0H2ZD72	Uncharacterized protein	1,01	NA
PA14_14660	A0A0H2ZF00	Putative outer membrane protein	1,01	NA
PA14_71890	A0A0H2ZJF3	Putative coenzyme A transferase	1,01	NA
PA14_14470	Q02RY8	Probable cytosol aminopeptidase (EC 3.4.11.1) (Leucine aminope	1,01	NA
PA14_64950	A0A0H2ZI18	Nicotinamidase	1,01	NA
PA14_08970	Q02T68	50S ribosomal protein L5	1,02	NA
PA14_68580	Q02EH2	Phosphoenolpyruvate carboxykinase [ATP] (PCK) (PEP carboxykin	1,02	NA
PA14_69390	A0A0H2ZIV8	Alginate regulatory protein AlgQ	1,02	NA
PA14_25600	A0A0H2ZD38	Putative peptidase	1,02	NA
PA14_61210	A0A0H2ZHH6	Glycerate dehydrogenase	1,02	NA
PA14_00790	A0A0H2ZK71	Oligopeptidase A	1,02	NA
PA14_54640	A0A0H2Z6K8	Probable enoyl-CoA hydratase/isomerase	1,02	NA
PA14_63310	A0A0H2ZGX1	tRNA 5-carboxymethoxyuridine methyltransferase (EC 2.1.1.-) (cm	1,02	NA
PA14_41230	Q02KU5	ATP-dependent Clp protease ATP-binding subunit ClpX	1,02	NA
PA14_49400	A0A0H2Z7Z4	Uncharacterized protein	1,02	NA
PA14_08360	Q02TB5	Indole-3-glycerol phosphate synthase (IGPS) (EC 4.1.1.48)	1,02	NA
PA14_30340	Q02NA3	Siroheme synthase [Includes: Uroporphyrinogen-III C-methyltrans	1,02	NA
PA14_30620	A0A0H2ZC40	Putative transcriptional regulator, AraC family	1,02	NA
PA14_03920	Q02UB7	Putrescine-binding periplasmic protein SpuD	1,02	NA
PA14_30190	Q02NB5	Isocitrate dehydrogenase [NADP] (IDH) (EC 1.1.1.42) (IDP) (NADP(	1,02	NA
PA14_04300	A0A0H2ZKQ5	Uncharacterized protein	1,02	NA
PA14_65230	Q02F80	Adenylosuccinate synthetase (AMPSase) (AdSS) (EC 6.3.4.4) (IMP-	1,02	NA
PA14_66260	A0A0H2ZIA9	Branched-chain amino acid transferase	1,02	NA
PA14_07840	A0A0H2ZLT6	Putative two-component response regulator	1,02	NA
PA14_71740	A0A0H2ZII0	Putative biotin carboxylase subunit of a transcarboxylase	1,02	NA
PA14_62230	A0A0H2ZGR2	Uncharacterized protein	1,02	NA
PA14_08510	Q02TA1	Iron-sulfur cluster insertion protein ErpA	1,02	NA
PA14_69370	A0A0H2ZIW7	Alginate regulatory protein AlgP	1,02	NA
PA14_30280	A0A0H2ZC61	Thioredoxin reductase (EC 1.8.1.9)	1,02	NA
PA14_24020	A0A0H2ZDD3	General secretion pathway protein G	1,02	NA
PA14_17690	A0A0H2ZES7	Putative glutamate synthase, large subunit	1,02	NA
PA14_24970	Q02PI7	Probable lipid kinase YegS-like (EC 2.7.1.-)	1,02	NA
PA14_04900	A0A0H2ZKT5	Signal recognition particle receptor FtsY (SRP receptor)	1,02	NA
PA14_71990	A0A0H2ZJC0	GDP-mannose 4,6-dehydratase (EC 4.2.1.47) (GDP-D-mannose de	1,02	NA
PA14_13580	A0A0H2ZFH0	Putative ABC-type proline/glycine betaine transpor	1,02	NA
PA14_04920	A0A0H2ZKG8	Cell division protein FtsX	1,02	NA
PA14_56690	A0A0H2ZGD3	Putative iron transport protein	1,02	NA
PA14_24150	A0A0H2ZC19	Uncharacterized protein	1,02	NA
PA14_23930	A0A0H2ZDH9	O-succinylhomoserine sulfhydrylase (OSH sulfhydrylase) (OSHS su	1,02	NA
PA14_40840	A0A0H2Z9K2	Putative protease	1,02	NA
PA14_41870	A0A0H2Z9D1	Transcriptional regulator CysB	1,02	NA
PA14_00170	A0A0H2ZJT5	Potassium uptake protein TrkA	1,02	NA
PA14_15860	A0A0H2ZDV9	Putative NUDIX hydrolase	1,02	NA
PA14_25390	Q02PF5	Soluble pyridine nucleotide transhydrogenase (STH) (EC 1.6.1.1) (I	1,02	NA
PA14_11260	A0A0H2ZG08	Putative NAD-dependent epimerase	1,02	NA
PA14_25690	A0A0H2ZD57	3-oxoacyl-[acyl-carrier-protein] synthase 2 (EC 2.3.1.179)	1,02	NA
PA14_56950	A0A0H2ZGV0	Putative DNA-binding response regulator ColR	1,02	NA
PA14_23070	A0A0H2ZDP9	Glucose-6-phosphate 1-dehydrogenase (G6PD) (EC 1.1.1.49)	1,02	NA
PA14_16930	A0A0H2ZEV8	Putative cysteine sulfinate desulfinase	1,02	NA
PA14_09890	A0A0H2ZG60	Peptidylprolyl isomerase (EC 5.2.1.8)	1,02	NA
PA14_61430	A0A0H2ZHD3	Uncharacterized protein	1,02	NA
PA14_03800	A0A0H2ZKS8	Anaerobically-induced outer membrane porin OprE	1,02	NA
PA14_16330	A0A0H2ZF28	Uncharacterized protein	1,02	NA
PA14_66760	A0A0H2ZIQ4	Putative cell division protein	1,02	NA
PA14_05640	A0A0H2ZK72	Putative outer membrane ferric siderophore receptor	1,02	NA

PA14_61400	A0A0H2ZHI8	Probable malate:quinone oxidoreductase (EC 1.1.5.4) (MQO) (Ma	1,02	NA
PA14_17520	A0A0H2ZEQ4	Putative competence-damaged protein	1,02	NA
PA14_42820	A0A0H2Z9P0	Putative hydrolase, isochorismatase family	1,02	NA
PA14_53430	A0A0H2Z7G5	Peptidyl-prolyl cis-trans isomerase (EC 5.2.1.8)	1,02	NA
PA14_67770	Q02EN5	2,3-bisphosphoglycerate-independent phosphoglycerate mutase (	1,02	NA
PA14_59610	A0A0H2ZH64	Uncharacterized protein	1,02	NA
PA14_57470	A0A0H2ZGH3	Ribosomal RNA small subunit methyltransferase I (EC 2.1.1.198) (1	1,02	NA
PA14_50980	A0A0H2Z6M3	Putative penicillin amidase	1,02	NA
PA14_67975	A0A0H2ZKV8	Putative outer membrane biogenesis protein	1,02	NA
PA14_08640	A0A0H2ZG75	Uncharacterized protein	1,02	NA
PA14_57410	A0A0H2ZGP8	UDP-N-acetylmuramoyl-L-alanyl-D-glutamate--2,6-diaminopimela	1,02	NA
PA14_19190	Q02QW0	NAD-dependent malic enzyme (NAD-ME) (EC 1.1.1.38)	1,02	NA
PA14_57030	A0A0H2ZGF2	Putative membrane protein	1,02	NA
PA14_65190	A0A0H2ZH91	23S rRNA (guanosine-2'-O-)-methyltransferase RlmB (EC 2.1.1.185	1,02	NA
PA14_70080	A0A0H2ZJD8	Leucine responsive regulatory protein	1,02	NA
PA14_20770	Q02QI1	STAS-domain containing protein PA14_20770	1,02	NA
PA14_68730	Q02EG1	Glutamate--cysteine ligase (EC 6.3.2.2) (Gamma-ECS) (GCS) (Gamr	1,02	NA
PA14_11910	A0A0H2ZFW1	Uncharacterized protein	1,02	NA
PA14_50460	A0A0H2Z7T3	Basal-body rod modification protein FlgD	1,02	NA
PA14_47540	A0A0H2Z8F8	Putative outer membrane protein	1,02	NA
PA14_24300	A0A0H2ZC15	Uncharacterized protein	1,02	NA
PA14_28280	A0A0H2ZBB1	Putative phenazine biosynthesis protein, PhzF family	1,02	NA
PA14_23370	A0A0H2ZDI5	Putative UDP-N-acetylglucosamine 2-epimerase	1,02	NA
PA14_33040	A0A0H2ZBK9	Aminomethyltransferase (EC 2.1.2.10)	1,02	NA
PA14_28730	A0A0H2ZB79	Putative transcriptional regulator	1,02	NA
PA14_68800	A0A0H2ZJ21	Putative phosphate transport regulator	1,02	NA
PA14_29970	A0A0H2ZC76	NADH dehydrogenase I chain F	1,02	NA
PA14_43280	A0A0H2Z9A9	Selenide, water dikinase (EC 2.7.9.3) (Selenium donor protein) (Se	1,02	NA
PA14_19350	A0A0H2ZE57	Putative peptidase or cellulase	1,02	NA
PA14_52160	A0A0H2Z697	Uncharacterized protein	1,02	NA
PA14_17440	Q02R98	tRNA pseudouridine synthase D (EC 5.4.99.27) (tRNA pseudouridi	1,02	NA
PA14_34820	A0A0H2Z9Y8	Putative regulatory protein	1,02	NA
PA14_25970	A0A0H2ZD16	Cobalamin biosynthetic protein CobN	1,02	NA
PA14_68400	A0A0H2ZIP1	Putative LysM domain protein	1,02	NA
PA14_32300	A0A0H2ZAG2	Putative kinase	1,02	NA
PA14_25130	A0A0H2ZD82	Uncharacterized protein	1,02	NA
PA14_58060	A0A0H2ZGT1	UPF0307 protein PA14_58060	1,02	NA
PA14_25780	A0A0H2ZD52	Putative deoxyribonuclease, TatD family	1,03	NA
PA14_66640	A0A0H2ZIF6	Type 4 fimbrial biogenesis protein PilO	1,03	NA
PA14_38480	A0A0H2ZA94	Alpha subunit of geranoyl-CoA carboxylase, GnyA	1,03	NA
PA14_52330	A0A0H2Z7N7	Uncharacterized protein	1,03	NA
PA14_51390	A0A0H2Z7U1	3-oxoacyl-[acyl-carrier-protein] synthase III	1,03	NA
PA14_16710	A0A0H2ZEW0	Putative protease with chaperone activity	1,03	NA
PA14_03370	A0A0H2ZJQ8	Uncharacterized protein	1,03	NA
PA14_18610	A0A0H2ZEJ6	Ornithine carbamoyltransferase, catabolic (OTCase) (EC 2.1.3.3)	1,03	NA
PA14_28050	A0A0H2ZBD0	Putative methyl-accepting chemotaxis protein	1,03	NA
PA14_01830	Q02UT0	Adenine deaminase (ADE) (EC 3.5.4.2) (Adenine aminohydrolase)	1,03	NA
PA14_57560	A0A0H2ZGY7	Cytochrome b	1,03	NA
PA14_11810	A0A0H2ZFS1	Putative aldehyde dehydrogenase	1,03	NA
PA14_18630	A0A0H2ZEM3	Putative serine protease	1,03	NA
PA14_52050	A0A0H2Z6A5	Phosphoribosylglycinamide formyltransferase (EC 2.1.2.2) (5'-pho	1,03	NA
PA14_54480	A0A0H2Z6Z5	tRNA-modifying protein YgfZ	1,03	NA
PA14_54660	A0A0H2Z6R7	Probable enoyl-CoA hydratase/isomerase	1,03	NA
PA14_53010	A0A0H2Z637	Aminotransferase (EC 2.6.1.-)	1,03	NA
PA14_57500	Q02H15	Phosphoheptose isomerase (EC 5.3.1.28) (Sedoheptulose 7-phosp	1,03	NA
PA14_06740	A0A0H2ZKE3	Cytochrome c-551	1,03	NA
PA14_27755	A0A0H2ZKT7	Probable glutathione S-transferase	1,03	NA
PA14_22980	A0A0H2ZDN1	Putative binding protein component of ABC sugar transporter	1,03	NA
PA14_67880	Q02EM6	Imidazole glycerol phosphate synthase subunit HisF (EC 4.1.3.-) (I(	1,03	NA

PA14_28920	A0A0H2ZB64	Putative chaperone	1,03	NA
PA14_60190	A0A0H2ZHB4	Chaperone protein ClpB	1,03	NA
PA14_51410	A0A0H2Z7A3	PqsC	1,03	NA
PA14_51280	A0A0H2Z7B1	Putative glycine cleavage system transcriptional repressor	1,03	NA
PA14_12060	A0A0H2ZEK5	FtsI / penicillin-binding protein 2	1,03	NA
PA14_25800	A0A0H2ZBS8	Putative transcriptional regulator, TetR family	1,03	NA
PA14_24010	A0A0H2ZDF1	General secretion pathway protein F	1,03	NA
PA14_58050	A0A0H2ZH23	PmbA protein	1,03	NA
PA14_51420	A0A0H2Z7K6	PqsB	1,03	NA
PA14_61040	A0A0H2ZHQ1	Catalase (EC 1.11.1.6)	1,03	NA
PA14_11700	A0A0H2ZEM2	Uncharacterized protein	1,03	NA
PA14_56780	A0A0H2ZGT4	Superoxide dismutase (EC 1.15.1.1)	1,03	NA
PA14_63090	Q02FQ1	L-lactate dehydrogenase (EC 1.1.-.-)	1,03	NA
PA14_48420	A0A0H2Z8D1	Putative transcriptional regulator	1,03	NA
PA14_05450	A0A0H2ZKX2	Ribosomal RNA small subunit methyltransferase E (EC 2.1.1.193)	1,03	NA
PA14_45300	A0A0H2Z8W9	Cytochrome C biogenesis protein CcmG	1,03	NA
PA14_18180	A0A0H2ZEP0	Putative Antibiotic biosynthesis monooxygenase	1,03	NA
PA14_22890	A0A0H2ZDK8	Glyceraldehyde-3-phosphate dehydrogenase (EC 1.2.1.-)	1,03	NA
PA14_15890	Q02RM4	Phosphoribosylglycinamide formyltransferase 2 (GART 2) (EC 2.1.:	1,03	NA
PA14_56070	A0A0H2ZGN7	Transcriptional regulator MvaT, P16 subunit	1,03	NA
PA14_09100	Q02T56	30S ribosomal protein S4	1,03	NA
PA14_33300	Q02MM2	CRISPR-associated endonuclease Cas6/Csy4 (EC 3.1.-.-)	1,03	NA
PA14_08470	A0A0H2ZKP5	Putative membrane protein	1,03	NA
PA14_23970	A0A0H2ZDH6	General secretion pathway protein D	1,03	NA
PA14_42790	A0A0H2Z8D9	50S ribosomal protein L3 glutamine methyltransferase (L3 MTase)	1,03	NA
PA14_28000	A0A0H2ZBD4	Uncharacterized protein	1,03	NA
PA14_72420	A0A0H2ZJS2	Putative GGDEF domain protein	1,03	NA
PA14_23460	A0A0H2ZDK1	Putative group 4 glycosyl transferase	1,03	NA
PA14_41470	A0A0H2Z8N2	Aconitate hydratase B (EC 4.2.1.3) (EC 4.2.1.99) (2-methylisocitrat	1,03	NA
PA14_23510	A0A0H2ZC86	UvrABC system protein B (Protein UvrB) (Excinuclease ABC subuni	1,03	NA
PA14_16950	A0A0H2ZEK0	2,3,4,5-tetrahydropyridine-2,6-dicarboxylate N-succinyltransferas	1,03	NA
PA14_41630	A0A0H2Z9E7	Putative cytoplasmic membrane-associated protein	1,03	NA
PA14_06290	Q02TT1	Malate synthase G (EC 2.3.3.9)	1,03	NA
PA14_27710	A0A0H2ZCG1	Uncharacterized protein	1,03	NA
PA14_23110	A0A0H2ZDJ6	Pseudouridine synthase (EC 5.4.99.-)	1,03	NA
PA14_17170	A0A0H2ZEQ6	Putative outer membrane protein OmpH	1,03	NA
PA14_30360	A0A0H2ZAW2	Putative anthranilate phosphoribosyltransferase	1,03	NA
PA14_38490	A0A0H2Z956	3-hydroxy-gamma-carboxygeranoyl-CoA lyase, GnyL	1,03	NA
PA14_21960	A0A0H2ZDS6	Putative ABC transporter, periplasmic substrate-binding protein	1,03	NA
PA14_44070	A0A0H2Z9C6	Citrate synthase	1,03	NA
PA14_70770	A0A0H2ZJ73	Putative metal ion transporter	1,03	NA
PA14_53950	A0A0H2Z6P5	Citrate synthase	1,03	NA
PA14_07520	A0A0H2ZLA4	RNA polymerase sigma factor RpoD (Sigma-70)	1,03	NA
PA14_69100	A0A0H2ZHW3	Putative flagellar protein FliL	1,03	NA
PA14_44080	A0A0H2Z976	Uncharacterized protein	1,03	NA
PA14_12130	Q02SG3	Lipoyl synthase (EC 2.8.1.8) (Lip-syn) (LS) (Lipoate synthase) (Lipoi	1,04	NA
PA14_53180	Q02I17	UPF0176 protein PA14_53180	1,04	NA
PA14_31060	A0A0H2ZC01	Uncharacterized protein	1,04	NA
PA14_10090	A0A0H2ZG96	Possible LysR family transcription regulator	1,04	NA
PA14_65570	A0A0H2ZII6	Uncharacterized protein	1,04	NA
PA14_47790	A0A0H2Z835	Cob(I)alamin adenosyltransferase	1,04	NA
PA14_16610	A0A0H2ZEP5	Putative lipoprotein	1,04	NA
PA14_51380	A0A0H2Z6F6	Quinolone signal response protein	1,04	NA
PA14_12570	A0A0H2ZFN2	Probable AsnC-family transcriptional regulator	1,04	NA
PA14_45940	A0A0H2Z8S3	Acyl-homoserine-lactone synthase (EC 2.3.1.184) (Autoinducer sy	1,04	NA
PA14_62830	Q02FS4	Triosephosphate isomerase (TIM) (TPI) (EC 5.3.1.1) (Triose-phospl	1,04	NA
PA14_60450	Q02GB0	50S ribosomal protein L27	1,04	NA
PA14_44680	A0A0H2Z913	Chromosome partition protein Smc	1,04	NA
PA14_22910	A0A0H2ZDQ9	6-phosphogluconate dehydratase	1,04	NA

PA14_51010	A0A0H2Z7F3	Uncharacterized protein	1,04	NA
PA14_73230	Q02DF5	ATP synthase epsilon chain (ATP synthase F1 sector epsilon subur	1,04	NA
PA14_69720	A0A0H2ZJA1	Putative hydrolase	1,04	NA
PA14_52990	A0A0H2Z794	Phenylalanine-4-hydroxylase	1,04	NA
PA14_65180	Q02F83	30S ribosomal protein S6	1,04	NA
PA14_14990	A0A0H2ZEY8	Putative oxidoreductase	1,04	NA
PA14_08890	Q02T76	30S ribosomal protein S19	1,04	NA
PA14_06340	A0A0H2ZKC2	Putative molybdenum transport regulator	1,04	NA
PA14_05230	A0A0H2ZKV7	Probable cystathionine gamma-lyase	1,04	NA
PA14_73140	A0A0H2ZIV3	Putative cyclopropan-fatty-acyl-phospholipid synthase	1,04	NA
PA14_66690	A0A0H2ZIG1	Putative protease	1,04	NA
PA14_62580	Q02FU3	3-methyl-2-oxobutanoate hydroxymethyltransferase 2 (EC 2.1.2.1	1,04	NA
PA14_68000	Q02EL6	Probable Fe(2+)-trafficking protein	1,04	NA
PA14_67450	A0A0H2ZIU4	Outer membrane lipoprotein Blc	1,04	NA
PA14_61290	A0A0H2ZHI0	Putative lipoprotein	1,04	NA
PA14_03680	A0A0H2ZKL7	Sulfate transport protein CysT	1,04	NA
PA14_70040	A0A0H2ZJB9	D-amino acid dehydrogenase (EC 1.4.99.-)	1,04	NA
PA14_17120	A0A0H2ZEJ0	Phosphatidate cytidylyltransferase (EC 2.7.7.41)	1,04	NA
PA14_00670	A0A0H2ZJX7	Uncharacterized protein	1,04	NA
PA14_71720	A0A0H2ZJA2	Putative transcarboxylase subunit	1,04	NA
PA14_28400	A0A0H2ZCK2	Putative outer membrane porin, OprD family	1,04	NA
PA14_12840	A0A0H2ZFL1	Possible acetyltransferase, GNAT family	1,04	NA
PA14_00720	A0A0H2ZJY2	Uncharacterized protein	1,04	NA
PA14_23750	Q02PT4	3-isopropylmalate dehydratase large subunit (EC 4.2.1.33) (Alpha-	1,04	NA
PA14_27920	A0A0H2ZCE5	Putative lipoprotein	1,04	NA
PA14_65280	A0A0H2ZI80	Protease subunit HflK	1,04	NA
PA14_66750	Q02EW6	Arginine--tRNA ligase (EC 6.1.1.19) (Arginyl-tRNA synthetase) (Arg	1,04	NA
PA14_67600	A0A0H2ZIV4	Glutamine synthetase (EC 6.3.1.2)	1,04	NA
PA14_11845	A0A0H2ZLZ2	UDP-N-acetylmuramate--L-alanyl-gamma-D-glutamyl-meso-2,6-di	1,04	NA
PA14_38440	A0A0H2ZAG9	Citronelloyl-CoA dehydrogenase, GnyD	1,04	NA
PA14_66150	A0A0H2ZI96	Uncharacterized protein	1,04	NA
PA14_11570	Q02SK9	Exodeoxyribonuclease 7 small subunit (EC 3.1.11.6) (Exodeoxyribo	1,04	NA
PA14_32280	A0A0H2ZBJ2	Uncharacterized protein	1,04	NA
PA14_30320	A0A0H2ZC58	Putative ATPase associated with chromosome architecture	1,04	NA
PA14_61360	A0A0H2ZGH6	Putative acetyltransferase	1,04	NA
PA14_38630	A0A0H2ZA83	Acetyl-CoA acetyltransferase	1,04	NA
PA14_24790	A0A0H2ZD83	Putative outer membrane porin	1,04	NA
PA14_05130	A0A0H2ZKH5	Putative YGGT family protein	1,04	NA
PA14_03770	A0A0H2ZKZ0	Agmatinase	1,04	NA
PA14_58180	Q02GV8	Glutamyl-tRNA(Gln) amidotransferase subunit A (Glu-ADT subunit	1,04	NA
PA14_29160	A0A0H2ZCE4	Uncharacterized protein	1,04	NA
PA14_11410	A0A0H2ZFM3	Riboflavin synthase alpha chain	1,04	NA
PA14_57820	A0A0H2ZH02	Uncharacterized protein	1,04	NA
PA14_43240	A0A0H2Z919	Uncharacterized protein	1,04	NA
PA14_44350	A0A0H2Z960	Putative cytochrome c oxidase subunit	1,04	NA
PA14_70140	A0A0H2ZJ32	Putative aldehyde dehydrogenase	1,04	NA
PA14_35810	A0A0H2ZAX7	Uncharacterized protein	1,04	NA
PA14_25640	Q02PD2	Phosphate acyltransferase (EC 2.3.1.n2) (Acyl-ACP phosphotransa	1,04	NA
PA14_48160	A0A0H2Z756	Putative sensor/response regulator hybrid	1,04	NA
PA14_29270	A0A0H2ZCD5	Putative outer membrane lipoprotein	1,04	NA
PA14_58820	A0A0H2ZGZ0	Uncharacterized protein	1,04	NA
PA14_57960	A0A0H2ZFQ5	Nitrogen regulatory IIA protein	1,04	NA
PA14_45110	A0A0H2Z953	Sulfate-binding protein of ABC transporter	1,04	NA
PA14_16160	A0A0H2ZDU1	Uncharacterized protein	1,04	NA
PA14_19430	A0A0H2ZD56	Putative acyl-CoA thiolase	1,04	NA
PA14_69010	Q02ED9	UPF0149 protein PA14_69010	1,04	NA
PA14_56000	A0A0H2ZG92	Chemotactic transducer PctA	1,04	NA
PA14_30750	Q02N70	Tryptophan 2,3-dioxygenase (TDO) (EC 1.13.11.11) (Tryptamin 2,3	1,04	NA
PA14_28180	A0A0H2ZCL9	Putative oxidoreductase	1,05	NA

PA14_07860	A0A0H2ZKK4	Polyamine-transporting ATPase (EC 3.6.3.31)	1,05	NA
PA14_01960	A0A0H2ZJX6	Putative RND efflux membrane fusion protein	1,05	NA
PA14_61870	A0A0H2ZHY0	Uncharacterized protein	1,05	NA
PA14_25220	A0A0H2ZD78	Uncharacterized protein	1,05	NA
PA14_17490	A0A0H2ZEH4	Ferredoxin I	1,05	NA
PA14_65380	A0A0H2ZIH8	Putative ATPase	1,05	NA
PA14_53420	A0A0H2Z616	Glutathione peroxidase	1,05	NA
PA14_62410	A0A0H2ZHR3	Uncharacterized protein	1,05	NA
PA14_18160	A0A0H2ZEJ8	Putative zinc-binding dehydrogenase	1,05	NA
PA14_51850	Q02IC2	Protein SlyX homolog	1,05	NA
PA14_54630	A0A0H2Z795	Probable acyl-CoA dehydrogenase	1,05	NA
PA14_38510	Q02LF6	Homogentisate 1,2-dioxygenase (HGDO) (EC 1.13.11.5) (Homoger	1,05	NA
PA14_69570	A0A0H2ZJA8	Magnesium/cobalt transport protein	1,05	NA
PA14_23720	A0A0H2ZDA9	Putative translation initiation inhibitor	1,05	NA
PA14_12760	A0A0H2ZFL6	Probable ATP-dependent RNA helicase	1,05	NA
PA14_28670	Q02NN9	50S ribosomal protein L35	1,05	NA
PA14_68300	A0A0H2ZIY9	Arginine/ornithine antiporter	1,05	NA
PA14_62120	A0A0H2ZHS5	Phosphatidylserine synthase	1,05	NA
PA14_50520	A0A0H2Z7T0	Branched-chain amino acid transport protein BraC	1,05	NA
PA14_66190	A0A0H2ZIC9	Uncharacterized protein	1,05	NA
PA14_65430	A0A0H2ZHA1	Chemotaxis protein MotB	1,05	NA
PA14_05080	Q02U23	Homoserine O-acetyltransferase (EC 2.3.1.31) (Homoserine O-trar	1,05	NA
PA14_05220	A0A0H2ZL24	Cystathionine beta-synthase	1,05	NA
PA14_72560	A0A0H2ZJL1	Transcriptional regulator np20	1,05	NA
PA14_43640	Q02K99	Glycerol-3-phosphate dehydrogenase [NAD(P)+] (EC 1.1.1.94) (NA	1,05	NA
PA14_57180	A0A0H2ZGW5	Putative cob(I)alamin adenosyltransferase	1,05	NA
PA14_55660	A0A0H2ZF98	RecBCD enzyme subunit RecD (EC 3.1.11.5) (Exonuclease V subun	1,05	NA
PA14_54040	A0A0H2Z6V7	Probable amino acid permease	1,05	NA
PA14_50880	A0A0H2Z7G1	Probable outer membrane protein	1,05	NA
PA14_07780	A0A0H2ZLT0	Putative phosphotransferase	1,05	NA
PA14_30400	A0A0H2ZBZ7	Putative sulfur reductase protein	1,05	NA
PA14_00020	A0A0H2ZIW1	DNA polymerase III subunit beta (EC 2.7.7.7)	1,05	NA
PA14_07700	Q02TH3	Bis(5'-nucleosyl)-tetraphosphatase, symmetrical (EC 3.6.1.41) (Ap	1,05	NA
PA14_58730	A0A0H2ZFW8	Type IV pilin structural subunit	1,05	NA
PA14_06750	A0A0H2ZLD6	Nitrite reductase	1,05	NA
PA14_57510	A0A0H2ZGN9	Putative secreted lipoprotein	1,05	NA
PA14_15710	A0A0H2ZEU9	Uncharacterized protein	1,05	NA
PA14_68500	A0A0H2ZHR8	Putative alcohol dehydrogenase, iron-containing	1,05	NA
PA14_24740	A0A0H2ZDC1	Uncharacterized protein	1,05	NA
PA14_23950	A0A0H2ZDF6	Putative short-chain dehydrogenase	1,05	NA
PA14_57770	A0A0H2ZFP0	Histidinol-phosphate aminotransferase (EC 2.6.1.9) (Imidazole ace	1,05	NA
PA14_66330	Q02EZ9	Peptide methionine sulfoxide reductase MsrA (Protein-methionin	1,05	NA
PA14_35520	A0A0H2ZB02	2-oxoisovalerate dehydrogenase, beta subunit	1,05	NA
PA14_72660	A0A0H2ZJT4	Putative amidase	1,05	NA
PA14_45260	A0A0H2Z944	Uncharacterized protein	1,05	NA
PA14_55650	A0A0H2ZGE2	Nuclease SbcCD subunit C	1,05	NA
PA14_16150	A0A0H2ZES6	Putative phage shock protein	1,05	NA
PA14_47610	A0A0H2Z8F4	Putative transcriptional regulator	1,05	NA
PA14_61710	Q02G08	Glutamyl-tRNA reductase (GluTR) (EC 1.2.1.70)	1,05	NA
PA14_17600	A0A0H2ZEP9	Putative secreted protein	1,05	NA
PA14_00770	A0A0H2ZJY8	Putative phosphatase	1,05	NA
PA14_21900	A0A0H2ZDT3	Putative HAD-superfamily hydrolase	1,06	NA
PA14_03860	A0A0H2ZKA8	Putative glutamine synthetase	1,06	NA
PA14_53230	A0A0H2Z6V6	Probable oxidoreductase	1,06	NA
PA14_12080	A0A0H2ZFQ6	Soluble lytic transglycosylase B	1,06	NA
PA14_66980	A0A0H2ZIF4	Sec-independent protein translocase protein TatC	1,06	NA
PA14_51830	A0A0H2Z6B9	Probable dna-binding stress protein	1,06	NA
PA14_35530	A0A0H2ZAU0	2-oxoisovalerate dehydrogenase (Alpha subunit)	1,06	NA
PA14_27510	Q02NZ0	Peptide methionine sulfoxide reductase MsrB (EC 1.8.4.12) (Pepti	1,06	NA

PA14_72460	A0A0H2ZJF2	Cytochrome c4	1,06	NA
PA14_45290	A0A0H2Z933	Cytochrome C-type biogenesis protein CcmH	1,06	NA
PA14_32520	A0A0H2ZAE8	Putative flavin-dependent oxidoreductase	1,06	NA
PA14_25270	A0A0H2ZD85	Aromatic amino acid transport protein AroP1	1,06	NA
PA14_72620	Q02DK6	Methionine import ATP-binding protein MetN 2 (EC 3.6.3.-)	1,06	NA
PA14_68260	A0A0H2ZHP8	Probable c4-dicarboxylate-binding protein	1,06	NA
PA14_16280	A0A0H2ZF31	Putative transciptional regulator	1,06	NA
PA14_22080	A0A0H2ZDJ8	Possible resolvase	1,06	NA
PA14_01930	A0A0H2ZK80	Transcriptional regulator PcaR	1,06	NA
PA14_70160	A0A0H2ZJE2	Putative pyridoxal-dependent aminotransferase	1,06	NA
PA14_38710	A0A0H2Z943	Uncharacterized protein	1,06	NA
PA14_24610	A0A0H2ZBZ5	Putative hydrolase	1,06	NA
PA14_46910	A0A0H2Z7E7	Putative binding protein component of ABC transporter	1,06	NA
PA14_18140	A0A0H2ZDC2	3-hydroxyisobutyrate dehydrogenase (HIBADH) (EC 1.1.1.31)	1,06	NA
PA14_20870	A0A0H2ZE66	Putative membrane protein	1,06	NA
PA14_44370	A0A0H2Z9C8	Cytochrome oxidase subunit (Cbb3-type)	1,06	NA
PA14_17480	A0A0H2ZEU1	RNA polymerase sigma factor RpoS (Sigma S) (Sigma-38)	1,06	NA
PA14_14510	A0A0H2ZE46	Putative permease	1,06	NA
PA14_16140	A0A0H2ZF40	Uncharacterized protein	1,06	NA
PA14_06330	A0A0H2ZKP1	Stress response kinase A (EC 2.7.11.1) (Serine/threonine-protein k	1,06	NA
PA14_38350	A0A0H2ZAA4	UTP--glucose-1-phosphate uridylyltransferase (EC 2.7.7.9) (UDP-g	1,06	NA
PA14_36450	A0A0H2ZAS6	Uncharacterized protein	1,06	NA
PA14_06970	A0A0H2ZLM3	Putative transcriptional regulator, Cro/CI family	1,06	NA
PA14_47670	Q02JC0	Nicotinate-nucleotide--dimethylbenzimidazole phosphoribosyltra	1,06	NA
PA14_01030	A0A0H2ZJ24	Uncharacterized protein	1,06	NA
PA14_61600	A0A0H2ZHK3	Putative oxidoreductase	1,06	NA
PA14_23620	A0A0H2ZDJ4	Putative 2-keto-3-deoxygluconate 6-phosphate aldolase	1,06	NA
PA14_67820	A0A0H2ZIW6	Putative divergent polysaccharide deacetylas	1,06	NA
PA14_29620	A0A0H2ZB04	Putative transcriptional regulator	1,06	NA
PA14_41640	Q02KR3	Putative 4-hydroxy-4-methyl-2-oxoglutarate aldolase (HMG aldola	1,06	NA
PA14_68620	A0A0H2ZIQ6	Uncharacterized protein	1,06	NA
PA14_31510	A0A0H2ZBP4	Putative short-chain dehydrogenase	1,06	NA
PA14_18740	Q02QZ6	Argininosuccinate synthase (EC 6.3.4.5) (Citrulline--aspartate ligas	1,06	NA
PA14_38660	A0A0H2ZAG0	Putative CoA transferase, subunit A	1,06	NA
PA14_54290	Q02HS5	Pyridoxine 5'-phosphate synthase (PNP synthase) (EC 2.6.99.2)	1,06	NA
PA14_03900	A0A0H2ZKM9	Putrescine aminotransferase	1,06	NA
PA14_64190	A0A0H2ZIA5	DNA-binding protein Fis	1,06	NA
PA14_69130	A0A0H2ZJ49	Glycerol-3-phosphate transporter	1,06	NA
PA14_08630	Q02T96	Type III pantothenate kinase (EC 2.7.1.33) (PanK-III) (Pantothenic	1,06	NA
PA14_70740	A0A0H2ZJF8	Putative exported protein	1,06	NA
PA14_05320	A0A0H2ZL28	Type IV pili response regulator PilG	1,06	NA
PA14_43110	A0A0H2Z9L7	Putative glutathione S-transferase	1,06	NA
PA14_45500	A0A0H2Z917	Putative purine-binding chemotaxis protein CheW	1,06	NA
PA14_03980	A0A0H2ZKN4	Putative penicillin amidase	1,06	NA
PA14_26610	A0A0H2ZCX6	Uncharacterized protein	1,06	NA
PA14_04160	A0A0H2ZKP7	Putatitve transcriptional regulator	1,07	NA
PA14_67240	A0A0H2ZHJ5	N-formylglutamate amidohydrolase	1,07	NA
PA14_02100	A0A0H2ZJA5	Uncharacterized protein	1,07	NA
PA14_47410	Q02JE0	UPF0260 protein PA14_47410	1,07	NA
PA14_09490	A0A0H2ZG51	Probable phenazine-specific methyltransferase	1,07	NA
PA14_51860	A0A0H2Z765	Uncharacterized protein	1,07	NA
PA14_25430	A0A0H2ZCX8	Putative lipoprotein releasing system, permease protein	1,07	NA
PA14_27580	A0A0H2ZBG2	Putative glutathione S-transferase	1,07	NA
PA14_61670	A0A0H2ZHK9	Molybdopterin biosynthesis MoeB protein	1,07	NA
PA14_38550	A0A0H2ZAE2	Maleylacetoacetate isomerase	1,07	NA
PA14_45630	A0A0H2Z926	RNA polymerase sigma factor FliA (RNA polymerase sigma factor f	1,07	NA
PA14_67840	A0A0H2ZIL7	Putative ABC-type amino acid transporter	1,07	NA
PA14_07680	A0A0H2ZKX3	Uncharacterized protein	1,07	NA
PA14_13730	A0A0H2ZF59	Putative nitrate/nitrite response regulator NarL	1,07	NA

PA14_09790	A0A0H2ZG74	Putative transcriptional regulator	1,07	NA
PA14_18880	A0A0H2ZEF6	Endonuclease III (EC 4.2.99.18) (DNA-(apurinic or apyrimidinic site	1,07	NA
PA14_09610	A0A0H2ZGG7	Uncharacterized protein	1,07	NA
PA14_61250	A0A0H2ZGG5	APC family lysine-specific permease	1,07	NA
PA14_00925	A0A0H2ZKR2	Uncharacterized protein	1,07	NA
PA14_52700	A0A0H2Z674	Arginine N-succinyltransferase (EC 2.3.1.109)	1,07	NA
PA14_41140	A0A0H2Z9I3	Putative ABC transporter, permease protein	1,07	NA
PA14_45640	A0A0H2Z8F9	Site-determining protein	1,07	NA
PA14_25550	A0A0H2ZD69	UDP-N-acetylenolpyruvoylglucosamine reductase (EC 1.3.1.98) (U	1,07	NA
PA14_14975	A0A0H2ZLX7	Uncharacterized protein	1,07	NA
PA14_68330	A0A0H2ZIP3	Arginine deiminase (ADI) (EC 3.5.3.6) (Arginine dihydrolase)	1,07	NA
PA14_44440	A0A0H2Z8T0	Putative cation-transporting P-type ATPase	1,07	NA
PA14_18890	Q02QY3	Electron transport complex subunit E	1,07	NA
PA14_08390	Q02TB2	S-adenosylmethionine decarboxylase proenzyme (AdoMetDC) (SA	1,07	NA
PA14_29730	A0A0H2ZC88	Putative two-component response regulator	1,07	NA
PA14_51310	A0A0H2Z6G2	Uncharacterized protein	1,07	NA
PA14_54690	A0A0H2Z791	Uncharacterized protein	1,07	NA
PA14_14520	A0A0H2ZF97	Putative membrane protein	1,07	NA
PA14_24880	A0A0H2ZD09	Putative lipoprotein	1,07	NA
PA14_14170	A0A0H2ZF32	Putative glutathione S-transferase	1,07	NA
PA14_66550	Q02EY3	Uroporphyrinogen decarboxylase (UPD) (URO-D) (EC 4.1.1.37)	1,07	NA
PA14_22740	A0A0H2ZDL8	Putative periplasmic inhibitor/zinc-resistance associated protein	1,07	NA
PA14_45590	A0A0H2Z8U8	Putative two-component sensor	1,07	NA
PA14_03855	A0A0H2ZM12	Probable periplasmic polyamine binding protein	1,07	NA
PA14_66460	A0A0H2ZIN6	Putative universal stress protein	1,07	NA
PA14_21220	A0A0H2ZDQ2	Uncharacterized protein	1,07	NA
PA14_36290	A0A0H2ZAV6	Putative NADP-dependent oxidoreductase	1,07	NA
PA14_20690	A0A0H2ZDU4	Uncharacterized protein	1,07	NA
PA14_72480	Q02DL7	Probable GTP-binding protein EngB	1,08	NA
PA14_16920	A0A0H2ZES2	Putative Fe-S center assembly protein	1,08	NA
PA14_70600	A0A0H2ZJ52	Putative DNA-binding protein HU family	1,08	NA
PA14_61450	A0A0H2ZHL3	Uncharacterized protein	1,08	NA
PA14_29920	Q02ND6	NADH-quinone oxidoreductase subunit I (EC 1.6.5.11) (NADH deh	1,08	NA
PA14_14020	A0A0H2ZFD5	Putative ornithine cyclodeaminase	1,08	NA
PA14_73360	Q02DE4	Ribosomal RNA small subunit methyltransferase G (EC 2.1.1.170) (	1,08	NA
PA14_18080	A0A0H2ZDC8	Putative transcriptional regulator, TetR family	1,08	NA
PA14_44880	A0A0H2Z974	Putative membrane protein	1,08	NA
PA14_22010	Q02Q76	Cell division topological specificity factor	1,08	NA
PA14_67440	A0A0H2ZIU2	Putative chlorohydrolase	1,08	NA
PA14_52010	A0A0H2Z7F6	Uncharacterized protein	1,08	NA
PA14_11140	A0A0H2ZG18	Putative nonribosomal peptide synthetase	1,08	NA
PA14_67250	Q02ES4	Imidazolonepropionase (EC 3.5.2.7) (Imidazolone-5-propionate hy	1,08	NA
PA14_34810	A0A0H2ZAZ7	Putative non-ribosomal peptide synthetase	1,08	NA
PA14_24760	A0A0H2ZD19	Putative membrane protein	1,08	NA
PA14_72850	A0A0H2ZJU6	Putative glutamine synthetase	1,08	NA
PA14_35080	A0A0H2ZB60	Putative arsenical resistance protein	1,08	NA
PA14_42230	A0A0H2Z8H9	Periplasmic beta-glucosidase	1,08	NA
PA14_72140	A0A0H2ZJQ2	Putative permease	1,08	NA
PA14_01730	A0A0H2ZKF2	Uncharacterized protein	1,08	NA
PA14_02270	A0A0H2ZKB6	Putative chemotaxis transducer	1,08	NA
PA14_13990	A0A0H2ZFH8	Putative amino acid ABC transporter	1,08	NA
PA14_30110	A0A0H2ZAX4	Adenylosuccinate lyase (ASL) (EC 4.3.2.2) (Adenylosuccinase)	1,08	NA
PA14_20880	A0A0H2ZDS9	Uncharacterized protein	1,08	NA
PA14_24730	Q02PK7	Deoxyguanosinetriphosphate triphosphohydrolase-like protein	1,08	NA
PA14_06600	A0A0H2ZLI5	Putative acyl-CoA dehydrogenase	1,08	NA
PA14_17380	A0A0H2ZEH9	Putative transcriptional regulator, LysR family	1,08	NA
PA14_37270	A0A0H2ZAN1	UPF0271 protein PA14_37270	1,08	NA
PA14_50570	A0A0H2Z7S6	Uncharacterized protein	1,08	NA
PA14_57300	A0A0H2ZGP2	Cell division protein FtsQ	1,08	NA

PA14_63050	A0A0H2ZGV1	Putative lipid transport protein	1,08	NA
PA14_13720	A0A0H2ZFJ9	Uncharacterized protein	1,08	NA
PA14_08490	A0A0H2ZLS8	Uncharacterized protein	1,08	NA
PA14_62170	A0A0H2ZHK7	Uncharacterized protein	1,08	NA
PA14_67510	A0A0H2ZIU7	Esterase EstA	1,08	NA
PA14_56370	A0A0H2ZGH5	Putative amidase/protease	1,08	NA
PA14_61660	Q02G12	Glutamate racemase (EC 5.1.1.3)	1,08	NA
PA14_37590	Q02LM8	Kynurenine formamidase (KFA) (KFase) (EC 3.5.1.9) (Arylformamic	1,08	NA
PA14_23890	A0A0H2ZDK5	Putative DedD protein	1,08	NA
PA14_02910	A0A0H2ZKF0	Putative transcriptional regulator, IclR family	1,08	NA
PA14_31690	A0A0H2ZBV6	Uncharacterized protein	1,09	NA
PA14_43460	A0A0H2Z879	Putative 3-hydroxyacyl-CoA dehydrogenase	1,09	NA
PA14_50430	A0A0H2Z825	Flagellar basal-body rod protein FlgG (Distal rod protein)	1,09	NA
PA14_22410	A0A0H2ZDS0	Uncharacterized protein	1,09	NA
PA14_68770	A0A0H2ZHT4	Acetylornithine deacetylase (EC 3.5.1.16)	1,09	NA
PA14_14610	A0A0H2ZF92	Putative preprotein translocase	1,09	NA
PA14_00110	A0A0H2ZIW5	DNA-3-methyladenine glycosidase I	1,09	NA
PA14_23210	A0A0H2ZDP1	Phosphoglycolate phosphatase (PGP) (PGPase) (EC 3.1.3.18)	1,09	NA
PA14_67930	Q02EM2	Imidazoleglycerol-phosphate dehydratase (IGPD) (EC 4.2.1.19)	1,09	NA
PA14_16260	A0A0H2ZEZ4	Putative oxidoreductase, FAD/FMN-binding	1,09	NA
PA14_47240	A0A0H2Z8Q5	Putative transcriptional regulator	1,09	NA
PA14_04510	A0A0H2ZKE6	Uncharacterized protein	1,09	NA
PA14_18600	A0A0H2ZD99	Putative ABC transporter, ATP-binding protein	1,09	NA
PA14_40830	A0A0H2ZA23	Putative alcohol dehydrogenase, zinc-containing	1,09	NA
PA14_63530	A0A0H2ZI04	Selenocysteine-specific elongation factor	1,09	NA
PA14_69090	A0A0H2ZIU6	Putative membrane fusion protein	1,09	NA
PA14_25520	Q02PE3	UPF0434 protein PA14_25520	1,09	NA
PA14_17330	A0A0H2ZER5	Cell division protein FtsB	1,09	NA
PA14_24590	A0A0H2ZDD1	Uncharacterized protein	1,09	NA
PA14_40980	A0A0H2Z9J3	Putative Enoyl-CoA hydratase	1,09	NA
PA14_06000	A0A0H2ZLE4	Putative ClpA/B protease ATP binding subunit	1,09	NA
PA14_71600	A0A0H2ZIH0	N5-carboxyaminoimidazole ribonucleotide synthase (N5-CAIR syn	1,09	NA
PA14_22760	A0A0H2ZDR9	Putative transcriptional regulator in 2-component system	1,09	NA
PA14_22000	A0A0H2ZCJ1	Ribosomal large subunit pseudouridine synthase A	1,09	NA
PA14_30700	A0A0H2ZAT9	Putative sensor/response regulator hybrid	1,09	NA
PA14_11490	A0A0H2ZEN4	Uncharacterized protein	1,09	NA
PA14_43350	A0A0H2Z9A7	Two-component sensor KdpD	1,09	NA
PA14_35490	A0A0H2ZB32	Dihydrolipoyl dehydrogenase (EC 1.8.1.4)	1,09	NA
PA14_08420	A0A0H2ZKP0	Putative HIT family protein	1,09	NA
PA14_56880	A0A0H2ZFI5	Putative membrane fusion protein	1,10	NA
PA14_40030	A0A0H2Z8W6	Putative enzyme	1,10	NA
PA14_53940	A0A0H2Z7C9	2-methylisocitrate lyase (2-MIC) (MICL) (EC 4.1.3.30) ((2R,3S)-2-m	1,10	NA
PA14_23410	A0A0H2ZDK6	Putative glycosyl transferase	1,10	NA
PA14_37210	Q02LQ6	UPF0317 protein PA14_37210	1,10	NA
PA14_37080	A0A0H2Z9G7	Putative transcriptional regulator	1,10	NA
PA14_69110	A0A0H2ZIT8	Putative oxidoreductase, zinc-binding	1,10	NA
PA14_54490	A0A0H2Z5X0	Uncharacterized protein	1,10	NA
PA14_51990	A0A0H2Z7Q0	NAD(P)H dehydrogenase (quinone) (EC 1.6.5.2) (NAD(P)H:quinone	1,10	NA
PA14_15360	A0A0H2ZF48	Uncharacterized protein	1,10	NA
PA14_41260	A0A0H2Z8P1	Putative two-component response regulator	1,10	NA
PA14_09150	A0A0H2ZGK4	Catalase (EC 1.11.1.6)	1,10	NA
PA14_30380	A0A0H2ZC74	Putative DsrH family protein	1,10	NA
PA14_14860	A0A0H2ZEZ2	Uncharacterized protein	1,10	NA
PA14_62510	Q02FU9	Glutamyl-Q tRNA(Asp) synthetase (Glu-Q-RSs) (EC 6.1.1.-)	1,10	NA
PA14_17420	Q02R99	2-C-methyl-D-erythritol 2,4-cyclodiphosphate synthase (MECDP-s	1,10	NA
PA14_60110	A0A0H2ZG88	Uncharacterized protein	1,10	NA
PA14_66440	A0A0H2ZHF7	Homocysteine synthase	1,10	NA
PA14_30080	A0A0H2ZC89	Uncharacterized protein	1,10	NA
PA14_44340	A0A0H2Z9B3	Putative cytochrome oxidase subunit (Cbb3-type)	1,10	NA

PA14_59150	A0A0H2ZH21	Single-stranded DNA-binding protein	1,10	NA
PA14_17550	A0A0H2ZEP2	Putative lysine decarboxylase	1,10	NA
PA14_20560	Q02QK0	Aliphatic amidase (EC 3.5.1.4) (Acylamide amidohydrolase)	1,10	NA
PA14_17110	A0A0H2ZEV2	Ditrans,polycis-undecaprenyl-diphosphate synthase ((2E,6E)-farn	1,10	NA
PA14_68350	A0A0H2ZIN7	Carbamate kinase	1,10	NA
PA14_53160	A0A0H2Z6Y2	Uncharacterized protein	1,11	NA
PA14_08440	A0A0H2ZLG1	Putative short chain alcohol dehydrogenase	1,11	NA
PA14_15990	Q02RL6	tRNA (guanine-N(1)-)-methyltransferase (EC 2.1.1.228) (M1G-met	1,11	NA
PA14_61120	A0A0H2ZHA9	Putative xanthine dehydrogenase accessory factor X	1,11	NA
PA14_03610	A0A0H2ZKL4	Putative putative Zn-dependent protease with chaperone functio	1,11	NA
PA14_05150	A0A0H2ZK42	Pyrroline-5-carboxylate reductase (P5C reductase) (P5CR) (EC 1.5.	1,11	NA
PA14_62680	A0A0H2ZGT7	Uncharacterized protein	1,11	NA
PA14_49690	A0A0H2Z7P0	Probable oxidoreductase	1,11	NA
PA14_04070	A0A0H2ZJW0	Uncharacterized protein	1,11	NA
PA14_11940	A0A0H2ZFS0	Uncharacterized protein	1,11	NA
PA14_33000	A0A0H2ZBD3	Glycine dehydrogenase (decarboxylating) (EC 1.4.4.2) (Glycine cle	1,11	NA
PA14_42850	A0A0H2Z9D3	GTP cyclohydrolase 1 (EC 3.5.4.16) (GTP cyclohydrolase I)	1,11	NA
PA14_57800	Q02GZ2	ATP phosphoribosyltransferase (ATP-PRT) (ATP-PRTase) (EC 2.4.2.	1,11	NA
PA14_13650	A0A0H2ZFF2	Putative sugar phosphatase	1,11	NA
PA14_61750	Q02G05	4-diphosphocytidyl-2-C-methyl-D-erythritol kinase (CMK) (EC 2.7.:	1,11	NA
PA14_19030	A0A0H2ZEJ9	Uncharacterized protein	1,11	NA
PA14_43710	A0A0H2Z8Y8	Putative methyl-accepting chemotaxis transducer	1,11	NA
PA14_66210	A0A0H2ZIA4	Putative lipopolysaccharide core biosynthesis protein	1,11	NA
PA14_21750	A0A0H2ZCL0	Putative acetyltransferase	1,11	NA
PA14_16790	A0A0H2ZEW6	Putative transcriptional regulator, tetR family	1,11	NA
PA14_69925	A0A0H2ZKR5	Pyruvate dehydrogenase (Cytochrome)	1,12	NA
PA14_56550	A0A0H2ZGC5	Uncharacterized protein	1,12	NA
PA14_04290	A0A0H2ZKW1	Putative integral membrane protein	1,12	NA
PA14_17580	A0A0H2ZEH0	Uncharacterized protein	1,12	NA
PA14_51710	A0A0H2Z7G9	Peptidoglycan associated lipoprotein OprL	1,12	NA
PA14_57170	A0A0H2ZGF9	Putative two-component response regulator	1,12	NA
PA14_58300	A0A0H2ZFT6	Putative DNA-binding response regulator	1,12	NA
PA14_19370	A0A0H2ZEF2	Putative asparagine synthetase, glutamine-hydrolyzing	1,12	NA
PA14_56900	A0A0H2ZGU3	Nicotinate phosphoribosyltransferase (NAPRTase) (EC 6.3.4.21)	1,12	NA
PA14_65520	A0A0H2ZI89	Uncharacterized protein	1,12	NA
PA14_20960	A0A0H2ZE61	Putativ isomerase	1,12	NA
PA14_57020	Q02H54	10 kDa chaperonin (GroES protein) (Protein Cpn10)	1,12	NA
PA14_19490	A0A0H2ZEH5	Putative thiol-specific antioxidant protein	1,12	NA
PA14_72510	Q02DL4	Homoserine kinase (HK) (HSK) (EC 2.7.1.39)	1,12	NA
PA14_04690	A0A0H2ZKS3	Uncharacterized protein	1,12	NA
PA14_64350	Q02FF4	Urease subunit gamma (EC 3.5.1.5) (Urea amidohydrolase subuni	1,12	NA
PA14_38140	A0A0H2ZA90	Putative glutamine synthetase	1,12	NA
PA14_68340	A0A0H2ZHQ2	Ornithine carbamoyltransferase, catabolic (OTCase) (EC 2.1.3.3)	1,12	NA
PA14_13320	A0A0H2ZFH7	Putative lipid carrier protein	1,12	NA
PA14_16370	A0A0H2ZEY1	Putative membrane protein	1,12	NA
PA14_00030	Q02V78	DNA replication and repair protein RecF	1,12	NA
PA14_22780	A0A0H2ZCD2	Putative cytoplasmic protein	1,12	NA
PA14_11720	A0A0H2ZFT5	Probable membrane protein	1,12	NA
PA14_63280	A0A0H2ZI49	Putative transcriptional regulator	1,12	NA
PA14_43040	A0A0H2Z8A7	Uncharacterized protein	1,12	NA
PA14_57970	Q02GX6	Nucleotide-binding protein PA14_57970	1,12	NA
PA14_24840	A0A0H2ZD97	Putative transcriptional regulator	1,12	NA
PA14_12360	A0A0H2ZFQ1	Uncharacterized protein	1,12	NA
PA14_04610	A0A0H2ZKR9	Putative xanthine/uracil permease family protein	1,12	NA
PA14_67700	A0A0H2ZIK4	Uncharacterized protein	1,12	NA
PA14_04080	A0A0H2ZKU5	Putative permease of ABC transporter	1,13	NA
PA14_39110	A0A0H2Z925	Uncharacterized protein	1,13	NA
PA14_19920	A0A0H2ZEB3	Dihydrolipoamide acetyltransferase component of pyruvate dehy	1,13	NA
PA14_53810	A0A0H2Z732	Uncharacterized protein	1,13	NA

PA14_32410	A0A0H2ZBR1	Transcriptional regulator MexT	1,13	NA
PA14_21710	A0A0H2ZDU8	Putative acetyltransferase	1,13	NA
PA14_20080	A0A0H2ZE98	Putative membrane protein	1,13	NA
PA14_17410	A0A0H2ZER0	S-formylglutathione hydrolase (EC 3.1.2.12)	1,13	NA
PA14_26480	A0A0H2ZD02	Precorrin-6y-dependent methyltransferase CobL	1,13	NA
PA14_57910	A0A0H2ZFQ0	Lipopolysaccharide export system protein LptC	1,13	NA
PA14_12770	A0A0H2ZFR5	Uncharacterized protein	1,13	NA
PA14_49640	A0A0H2Z844	Probable transcriptional regulator	1,13	NA
PA14_12970	A0A0H2ZFL5	TauD	1,13	NA
PA14_21760	A0A0H2ZDX1	Cold acclimation protein B	1,13	NA
PA14_56890	A0A0H2ZGE4	Putative RND efflux transporter	1,13	NA
PA14_61470	Q02G28	Uracil phosphoribosyltransferase (EC 2.4.2.9) (UMP pyrophospho	1,13	NA
PA14_53200	A0A0H2Z779	Uncharacterized protein	1,13	NA
PA14_23760	Q02PT3	3-isopropylmalate dehydratase small subunit (EC 4.2.1.33) (Alpha	1,14	NA
PA14_36270	A0A0H2Z9N2	Putative 3-hydroxyisobutyrate dehydrogenase	1,14	NA
PA14_71640	A0A0H2ZJL4	Putative transcriptional regulator, LysR family	1,14	NA
PA14_60860	A0A0H2ZHF9	Transcriptional regulatory protein NfxB	1,14	NA
PA14_40780	A0A0H2Z9K9	Uncharacterized protein	1,14	NA
PA14_68700	A0A0H2ZIR0	Two-component response regulator OmpR	1,14	NA
PA14_64920	A0A0H2ZIA7	Putative methyl-accepting chemotaxis protein	1,14	NA
PA14_14450	A0A0H2ZE51	Uncharacterized protein	1,14	NA
PA14_53000	Q02I31	Putative pterin-4-alpha-carbinolamine dehydratase (PHS) (EC 4.2.	1,14	NA
PA14_00440	Q02V45	Tryptophan synthase alpha chain (EC 4.2.1.20)	1,14	NA
PA14_25820	A0A0H2ZD26	Putative lipoprotein	1,14	NA
PA14_56420	A0A0H2ZGR1	Putative membrane protein	1,14	NA
PA14_40080	A0A0H2Z8W1	Uncharacterized protein	1,14	NA
PA14_21830	A0A0H2ZCK7	Uncharacterized protein	1,14	NA
PA14_03880	A0A0H2ZKT2	Glutamine synthetase	1,14	NA
PA14_50510	A0A0H2Z7V5	Uncharacterized protein	1,14	NA
PA14_12670	A0A0H2ZFE3	Possible nuclease or phosphatase	1,14	NA
PA14_51730	A0A0H2Z7R1	TolA protein	1,15	NA
PA14_66610	Q02EX8	Shikimate kinase (SK) (EC 2.7.1.71)	1,15	NA
PA14_48810	A0A0H2Z8E5	Putative transcriptional regulator, Sir2 family	1,15	NA
PA14_20900	A0A0H2ZCT0	Putative MFS transporter	1,15	NA
PA14_19740	A0A0H2ZD28	Putative enoyl CoA-hydratase/isomerase family protein	1,15	NA
PA14_05160	A0A0H2ZL20	Putative PLP dependent enzyme	1,15	NA
PA14_66340	A0A0H2ZIB4	Ribosomal RNA large subunit methyltransferase J (EC 2.1.1.266) (2	1,15	NA
PA14_50760	A0A0H2Z7H1	Putative glycerate kinase	1,15	NA
PA14_56590	A0A0H2ZGK0	Putative universal stress protein	1,15	NA
PA14_68470	A0A0H2ZIZ8	Uncharacterized protein	1,15	NA
PA14_16740	A0A0H2ZDL2	Putative carboxyvinyl-carboxyphosphonate phosphorylmutase	1,15	NA
PA14_12850	A0A0H2ZFR1	Probable acetyltransferase	1,15	NA
PA14_50500	A0A0H2Z7J5	Uncharacterized protein	1,15	NA
PA14_57200	A0A0H2ZGN8	Putative glutathione S-transferase	1,15	NA
PA14_49840	A0A0H2Z7X2	Probable deoxyguanosinetriphosphate triphosphohydrolase (dGT	1,16	NA
PA14_60560	A0A0H2ZHM4	Uncharacterized protein	1,16	NA
PA14_09810	A0A0H2ZG65	Uncharacterized protein	1,16	NA
PA14_45960	A0A0H2Z901	Transcriptional regulator LasR	1,16	NA
PA14_66380	Q02EZ6	K(+)/H(+) antiporter NhaP2 (Potassium/proton antiporter NhaP2)	1,16	NA
PA14_11750	A0A0H2ZFJ6	Probable acetyltransferase	1,16	NA
PA14_20890	Q02QH1	ADP-L-glycero-D-manno-heptose-6-epimerase (EC 5.1.3.20) (ADP-	1,16	NA
PA14_19730	A0A0H2ZE22	Putative oxidoreductase, short-chain dehydrogenase/reductase fa	1,16	NA
PA14_23810	A0A0H2ZDA7	Putative aspartate-semialdehyde dehydrogenase	1,16	NA
PA14_42730	Q02KH4	Acireductone dioxygenase (1,2-dihydroxy-3-keto-5-methylthioper	1,16	NA
PA14_49280	A0A0H2Z802	Probable transglycolase	1,16	NA
PA14_45680	A0A0H2Z8U3	Flagellar biosynthesis protein FlhA	1,16	NA
PA14_18120	A0A0H2ZEC6	Methylmalonate-semialdehyde dehydrogenase	1,16	NA
PA14_20140	A0A0H2ZE93	Ferredoxin--NADP+ reductase	1,16	NA
PA14_09030	Q02T62	50S ribosomal protein L30	1,16	NA

PA14_27280	A0A0H2ZCT8	Putative transcriptional regulator, LysR family	1,16	NA
PA14_60300	A0A0H2ZHC4	Type 4 fimbrial biogenesis protein PilX	1,16	NA
PA14_25305	Q02PG2	Na(+)-translocating NADH-quinone reductase subunit B (Na(+)-NC	1,17	NA
PA14_31430	A0A0H2ZBX4	Uncharacterized protein	1,17	NA
PA14_33780	A0A0H2ZA66	Putative transmembrane sensor	1,17	NA
PA14_51350	A0A0H2Z7L1	Anthranilate synthase component II	1,17	NA
PA14_14960	A0A0H2ZFB9	Putative amidohydrolase	1,17	NA
PA14_06830	A0A0H2ZLE2	Nitric-oxide reductase subunit B	1,17	NA
PA14_41030	A0A0H2Z8Q2	Uncharacterized protein	1,17	NA
PA14_28440	A0A0H2ZCL5	Uncharacterized protein	1,17	NA
PA14_72260	A0A0H2ZJJ1	Putative acetyltransferase	1,17	NA
PA14_49020	A0A0H2Z706	Uncharacterized protein	1,17	NA
PA14_66820	A0A0H2ZIG5	Poly(3-hydroxyalkanoic acid) synthase 1	1,17	NA
PA14_56220	A0A0H2ZFE1	Putative universal stress protein	1,17	NA
PA14_68280	A0A0H2ZIN2	Probable dicarboxylate transporter	1,17	NA
PA14_06120	A0A0H2ZL85	Putative glutathione S-transferase	1,18	NA
PA14_67320	Q02ER8	Histidine ammonia-lyase (Histidase) (EC 4.3.1.3)	1,18	NA
PA14_47440	A0A0H2Z856	Putative 2-hydroxyacid dehydrogenase	1,18	NA
PA14_41830	A0A0H2Z8K8	Homoserine kinase	1,18	NA
PA14_42860	Q02KG3	Bacteriohemerythrin	1,18	NA
PA14_43840	A0A0H2Z8X9	Putative dienelactone hydrolase family protein	1,18	NA
PA14_14010	A0A0H2ZE79	Putative amino acid oxidase	1,18	NA
PA14_06400	A0A0H2ZKP6	Putative transcriptional regulator, LysR family	1,18	NA
PA14_71630	A0A0H2ZJM2	Alcohol dehydrogenase, zinc-containing	1,18	NA
PA14_29600	A0A0H2ZC22	6-carboxy-5,6,7,8-tetrahydropterin synthase (EC 4.-.-.-)	1,18	NA
PA14_43650	A0A0H2Z999	Putative outer membrane receptor protein	1,18	NA
PA14_57100	A0A0H2ZGW0	Putative permease	1,19	NA
PA14_62470	Q02FV2	Sugar fermentation stimulation protein homolog	1,19	NA
PA14_05520	A0A0H2ZL40	Multidrug resistance operon repressor MexR	1,19	NA
PA14_35270	A0A0H2Z9V7	Putative cytochrome c	1,19	NA
PA14_01400	A0A0H2ZJ57	Putative acyltransferase	1,19	NA
PA14_45310	A0A0H2Z7Q9	Cytochrome C-type biogenesis protein CcmF	1,19	NA
PA14_22250	A0A0H2ZDW1	Uncharacterized protein	1,19	NA
PA14_56790	A0A0H2ZGK3	Putative GGDEF domain/EAL domain protein	1,19	NA
PA14_25670	A0A0H2ZD34	Acyl carrier protein (ACP)	1,19	NA
PA14_19380	A0A0H2ZED2	Putative transcriptional regulator	1,19	NA
PA14_36010	A0A0H2ZAQ2	Uncharacterized protein	1,20	NA
PA14_03830	A0A0H2ZKM5	N-carbamoylputrescine amidohydrolase	1,20	NA
PA14_44480	A0A0H2Z9C0	Uncharacterized protein	1,20	NA
PA14_40770	A0A0H2ZA28	Sulfite reductase	1,20	NA
PA14_27460	Q02NZ5	Thiopurine S-methyltransferase (EC 2.1.1.67) (Thiopurine methylt	1,20	NA
PA14_43320	Q02KC3	Glutaminase (EC 3.5.1.2)	1,20	NA
PA14_12550	A0A0H2ZEI1	Uncharacterized protein	1,20	NA
PA14_41840	Q02KP7	Phosphoadenosine phosphosulfate reductase (EC 1.8.4.8) (3'-pho	1,20	NA
PA14_13240	A0A0H2ZFJ5	Molybdopterin synthase sulfur carrier subunit	1,21	NA
PA14_39280	A0A0H2ZA34	Ribokinase (RK) (EC 2.7.1.15)	1,21	NA
PA14_57710	A0A0H2ZGI7	ATP sulfurylase GTP-binding subunit/APS kinase	1,21	NA
PA14_72580	Q02DK9	Zinc import ATP-binding protein ZnuC (EC 3.6.3.-)	1,21	NA
PA14_00450	A0A0H2ZK36	Tryptophan synthase beta chain (EC 4.2.1.20)	1,21	NA
PA14_70790	A0A0H2ZJG2	Putative two-component response regulator	1,21	NA
PA14_57380	Q02H25	Phospho-N-acetylmuramoyl-pentapeptide-transferase (EC 2.7.8.1	1,22	NA
PA14_56540	A0A0H2ZFG1	Putative flavoprotein	1,22	NA
PA14_53470	Q02HZ4	Acetate kinase (EC 2.7.2.1) (Acetokinase)	1,22	NA
PA14_52930	A0A0H2Z652	Probable transcriptional regulator	1,22	NA
PA14_70930	A0A0H2ZJ67	Putative long-chain acyl-CoA thioester hydrolase	1,22	NA
PA14_11770	A0A0H2ZEL8	Ethanolamine ammonia-lyase large subunit, EutB	1,22	NA
PA14_04710	A0A0H2ZKF6	Uncharacterized protein	1,22	NA
PA14_03700	A0A0H2ZKY4	Sulfate-binding protein	1,22	NA
PA14_54590	Q02HQ1	Uracil-DNA glycosylase (UDG) (EC 3.2.2.27)	1,22	NA

PA14_43620	A0A0H2Z8Z2	Putative phosphohistidine phosphatase SixA	1,23	NA
PA14_04860	A0A0H2ZKG3	Ribosomal RNA small subunit methyltransferase D (EC 2.1.1.171)	1,23	NA
PA14_00160	A0A0H2ZIW8	Putative tetratricopeptide repeat domain	1,23	NA
PA14_17570	A0A0H2ZET8	Putative metallo-beta-lactamase superfamily protein	1,23	NA
PA14_25830	A0A0H2ZD47	Uncharacterized protein	1,23	NA
PA14_31720	A0A0H2ZBU0	Putative lysophospholipas	1,23	NA
PA14_59840	A0A0H2ZH89	Uncharacterized protein	1,23	NA
PA14_06250	A0A0H2ZL23	Putative acetyltransferase, GNAT family	1,24	NA
PA14_53600	A0A0H2Z609	Uncharacterized protein	1,24	NA
PA14_61010	A0A0H2ZGF1	Uncharacterized protein	1,24	NA
PA14_41970	A0A0H2Z8J8	Uncharacterized protein	1,24	NA
PA14_09210	A0A0H2ZGC4	Salicylate biosynthesis isochorismate synthase	1,24	NA
PA14_21730	A0A0H2ZDL9	Putative TonB-dependent receptor	1,24	NA
PA14_23630	A0A0H2ZDH0	Putative thioesterase	1,24	NA
PA14_07790	A0A0H2ZKX8	Putative nucleotidyltransferase	1,24	NA
PA14_53210	A0A0H2Z628	Uncharacterized protein	1,25	NA
PA14_41440	A0A0H2Z9V8	Putative universal stress protein	1,25	NA
PA14_09290	A0A0H2ZG63	Pyochelin biosynthetic protein PchG	1,25	NA
PA14_16250	Q02RJ6	Elastase (EC 3.4.24.26) [Cleaved into: Pro-elastase]	1,25	NA
PA14_00480	A0A0H2ZJV9	Uncharacterized protein	1,25	NA
PA14_06700	A0A0H2ZLD1	Heme d1 biosynthesis protein NirL	1,25	NA
PA14_43600	A0A0H2Z854	Putative thioesterase	1,25	NA
PA14_16910	A0A0H2ZET6	Uncharacterized protein	1,26	NA
PA14_49350	A0A0H2Z8B6	Uncharacterized protein	1,26	NA
PA14_46490	A0A0H2Z8T3	3-oxoacyl-[acyl-carrier-protein] synthase 2 (EC 2.3.1.179)	1,26	NA
PA14_37070	A0A0H2ZAK0	Putative phosphoadenosine phosphosulfate sulfotransferase	1,26	NA
PA14_04580	A0A0H2ZJZ7	Dihydrofolate reductase (EC 1.5.1.3)	1,26	NA
PA14_26780	A0A0H2ZCM8	Uncharacterized protein	1,26	NA
PA14_16180	A0A0H2ZEZ8	Uncharacterized protein	1,27	NA
PA14_24940	A0A0H2ZDA4	Putative oxidase	1,27	NA
PA14_51770	A0A0H2Z7G4	Uncharacterized protein	1,27	NA
PA14_40280	A0A0H2ZA60	Uncharacterized protein	1,27	NA
PA14_29150	A0A0H2ZCF9	Uncharacterized protein	1,27	NA
PA14_05960	A0A0H2ZK92	Putative major cold shock protein	1,28	NA
PA14_69030	A0A0H2ZIU1	Cell division protein ZapA	1,29	NA
PA14_21180	A0A0H2ZE14	Putative 3-hydroxyisobutyrate dehydrogenase	1,29	NA
PA14_13010	A0A0H2ZEE8	Uncharacterized protein	1,29	NA
PA14_15230	Q02RT0	Exodeoxyribonuclease 7 large subunit (EC 3.1.11.6) (Exodeoxyribc	1,29	NA
PA14_23700	A0A0H2ZDL4	Transcriptional regulator, LysR family	1,29	NA
PA14_13500	A0A0H2ZFH4	Putative D-isomer specific 2-hydroxyacid dehydroge	1,30	NA
PA14_28630	A0A0H2ZCI2	Putative hydrolase	1,30	NA
PA14_00640	A0A0H2ZJN6	Potential phenazine-modifying enzyme	1,30	NA
PA14_13000	A0A0H2ZFB6	Probable transcriptional regulator	1,30	NA
PA14_38590	A0A0H2ZA47	3-hydroxybutyrate dehydrogenase	1,30	NA
PA14_03650	A0A0H2ZJS9	Sulfate/thiosulfate import ATP-binding protein CysA (EC 3.6.3.25)	1,30	NA
PA14_68230	A0A0H2ZIY4	Probable two-component sensor	1,31	NA
PA14_04150	A0A0H2ZKV0	Putative fumarylacetoacetate hydrolase family protein	1,31	NA
PA14_22990	A0A0H2ZDK3	Putative permease of ABC sugar transporter	1,31	NA
PA14_06030	A0A0H2ZL80	Putative 1-acyl-sn-glycerol-3-phosphate acyltransferase	1,31	NA
PA14_66770	Q02EW4	ATP-dependent protease subunit HslV (EC 3.4.25.2)	1,32	NA
PA14_53850	A0A0H2Z6W7	Probable oxidoreductase	1,32	NA
PA14_32985	A0A0H2ZLK7	Glycine cleavage system H protein	1,32	NA
PA14_49900	A0A0H2Z6V5	Probable outer membrane protein	1,32	NA
PA14_67350	Q02ER6	Urocanate hydratase (Urocanase) (EC 4.2.1.49) (Imidazolonepropi	1,33	NA
PA14_50280	A0A0H2Z7K5	Uncharacterized protein	1,33	NA
PA14_44180	A0A0H2Z9B9	Putative transcriptional regulator, lysR family	1,33	NA
PA14_09380	A0A0H2ZF74	Putative transporter	1,33	NA
PA14_07380	A0A0H2ZL94	Uncharacterized protein	1,35	NA
PA14_23850	Q02PS6	N-(5'-phosphoribosyl)anthranilate isomerase (PRAI) (EC 5.3.1.24)	1,35	NA

PA14_29900	A0A0H2ZC14	NADH dehydrogenase I chain J	1,35	NA
PA14_18275	A0A0H2ZLI3	Phosphotransferase system, fructose-specific IIBC component	1,36	NA
PA14_24600	A0A0H2ZD29	Putative carboxypeptidase	1,36	NA
PA14_46630	A0A0H2Z8U9	Putative phenazine biosynthesis protein	1,36	NA
PA14_09280	A0A0H2ZGJ4	Pyochelin synthetase PchF	1,37	NA
PA14_34490	A0A0H2ZB23	Putative acyl-CoA dehydrogenase	1,37	NA
PA14_30370	A0A0H2ZC57	Sulfurtransferase (EC 2.8.1.-)	1,37	NA
PA14_19560	A0A0H2ZEG9	Alkanesulfonate monooxygenase (EC 1.14.14.5) (FMNH2-depende	1,37	NA
PA14_09270	A0A0H2ZGB9	Pyochelin synthetase	1,38	NA
PA14_16470	A0A0H2ZDR7	Putative chemotaxis sensor/effector fusion protein	1,38	NA
PA14_03670	A0A0H2ZKS1	Sulfate transport protein CysW	1,39	NA
PA14_24100	A0A0H2ZDG6	Type II secretion system protein M (T2SS protein M) (General secr	1,39	NA
PA14_00940	A0A0H2ZK83	Uncharacterized protein	1,39	NA
PA14_09240	A0A0H2ZF83	Pyochelin biosynthesis protein PchD	1,40	NA
PA14_72500	A0A0H2ZJS5	Uncharacterized protein	1,40	NA
PA14_34460	A0A0H2ZB86	Alkyl hydroperoxide reductase AhpD (EC 1.11.1.15)	1,41	NA
PA14_57720	Q02GZ8	Sulfate adenylyltransferase subunit 2 (EC 2.7.7.4) (ATP-sulfurylase	1,41	NA
PA14_19580	Q02QT1	Aliphatic sulfonates import ATP-binding protein SsuB 2 (EC 3.6.3.-	1,41	NA
PA14_04930	A0A0H2ZK26	RNA polymerase sigma factor RpoH (RNA polymerase sigma-32 fa	1,41	NA
PA14_65310	Q02F74	RNA-binding protein Hfq	1,42	NA
PA14_66890	A0A0H2ZIG9	Uncharacterized protein	1,44	NA
PA14_70170	A0A0H2ZJC7	Uncharacterized protein	1,44	NA
PA14_15020	A0A0H2ZF72	Uncharacterized protein	1,44	NA
PA14_07850	A0A0H2ZKY3	Putative binding protein component of ABC transporter	1,45	NA
PA14_49990	A0A0H2Z7M0	Uncharacterized protein	1,45	NA
PA14_29120	A0A0H2ZC52	Uncharacterized protein	1,45	NA
PA14_16730	A0A0H2ZEL7	Uncharacterized protein	1,46	NA
PA14_27360	A0A0H2ZCT2	Putative enoyl-CoA hydratase	1,49	NA
PA14_09220	A0A0H2ZGJ9	Salicylate biosynthesis protein PchB	1,52	NA
PA14_67400	A0A0H2ZIJ4	Putative ABC transporter, periplasmic substrate-binding protein	1,53	NA
PA14_67500	A0A0H2ZIJ3	Lactoylglutathione lyase (EC 4.4.1.5) (Glyoxalase I)	1,56	NA
PA14_16220	A0A0H2ZDT5	Single-stranded-DNA-specific exonuclease RecJ	1,58	NA
PA14_36200	A0A0H2Z9N7	Putative binding protein component of ABC transporter	1,58	NA
PA14_09230	A0A0H2ZG68	Pyochelin biosynthetic protein PchC	1,59	NA
PA14_19540	A0A0H2ZEC2	Putative ABC-type transporter periplasmic sulfonate-binding prot	1,60	NA
PA14_60830	A0A0H2ZHN8	Multidrug efflux RND transporter MexD	1,60	NA
PA14_09340	A0A0H2ZGB5	Fe(III)-pyochelin outer membrane receptor FptA	1,60	NA
PA14_09320	A0A0H2ZGC6	Putative ATP-binding component of ABC transporter	1,62	NA
PA14_60850	A0A0H2ZHG8	Multidrug efflux RND membrane fusion protein MexC	1,63	NA
PA14_07410	A0A0H2ZKV9	Uncharacterized protein	1,68	NA
PA14_09300	A0A0H2ZF78	Putative ATP-binding component of ABC transporter	1,69	NA
PA14_30820	A0A0H2ZBX1	Putative methyl-accepting chemotaxis transducer	1,73	NA
PA14_56730	A0A0H2ZGK9	Uncharacterized protein	1,78	NA
PA14_25790	A0A0H2ZCV2	Putative selenocysteine lyase	1,79	NA
PA14_00240	Q02V59	Threonylcarbamoyl-AMP synthase (TC-AMP synthase) (EC 2.7.7.8	1,92	NA
PA14_03166	A0A0H2ZLW4	Uncharacterized protein	1,97	NA
PA14_35800	A0A0H2ZAZ5	Uncharacterized protein	2,05	0,850
PA14_67530	A0A0H2ZIK3	Putative membrane protein	2,40	0,626

2384 proteins with at least 2 peptide counts

* proteins showing Q-value (minimal false discovery rate) < 0.01

NA, not applicable: proteins with fold changes (FC) > -2 and < 2
