# Supplementary material for: Plant-Derived Catechols Are Substrates of TonB-Dependent Transporters and Sensitize Pseudomonas aeruginosa to Siderophore-Drug Conjugates
Source: mBio. 2022 Jun 30;13(4):e01498-22. doi: 10.1128/mbio.01498-22 (PMC9426570; doi:10.1128/mbio.01498-22)
Supplement: TABLE S3 [file mbio.01498-22-s0006.pdf]

Table S3. Bacterial strains and plasmids

Strains/plasmids	Relevant characteristics	Reference, source
P. aeruginosa		
PAO1	PAO1, *mexT* non-functional	Laboratory collection
Δ*piuA*	unmarked deletion of *piuA* gene	(1)
Δ*pirA*	unmarked deletion of *pirA* gene	(2)
Δ*piuA*Δ*pirA*	unmarked deletion of *pirA, piuA* genes	(2)
Δ*pirR*	unmarked deletion of *pirR* gene	This study
Δ*pirS*	unmarked deletion of *pirS* gene	This study
PA14		
Δ*pvdpch*	unmarked deletion of *pvdL and pchABCD operon* (deficient in Pvd and Pch synthesis)	D. Pletzer
Δ*pvdpch*Δ*piuA*	unmarked deletion of *piuA* gene	D. Pletzer
Δ*pvdpch*Δ*pirA*	unmarked deletion of *pirA* gene	This study
Δ*pvdpch*Δ*piuApirA*	unmarked deletion of *piuA and pirA* gene	This study
E. coli		
S17λpir	*pro thi hsdR*$^+$ Tmp^r Sm^r*;* chromosome::RP4-2 Tc::Mu-Kan::Tn7/λpir	(3)
**Plasmids**		
pEXG2	gene replacement vector, Gm-R	(4)
pIApX2	Broad-host range expression vector, Ap-R	I. Attree (Grenoble)
pBBR-gfp	GFP promoter fusion vector, Ap-R	(5)

pirAp-GFP	*pirA* promoter fusion in pBBR1-GFP, Ap-R	This study
piuAp-GFP	*piuA* promoter fusion in pBBR1-GFP, Ap-R	This study
ppirR	constitutive *pirR* expression in vector pIApX2	This study
ppirS	constitutive *pirR* expression in vector pIApX2	This study

References

1. Van Delden C, Page MG, Köhler T. Involvement of Fe uptake systems and AmpC beta-lactamase in susceptibility to the siderophore monosulfactam BAL30072 in *Pseudomonas aeruginosa*. Antimicrob Agents Chemother. 2013;57(5):2095-102.
2. Moynié L, Luscher A, Rolo D, Pletzer D, Tortajada A, Weingart H, Braun Y, Page MG, Naismith JH, Köhler T. Structure and Function of the PiuA and PirA Siderophore-Drug Receptors from *Pseudomonas aeruginosa* and *Acinetobacter baumannii*. Antimicrob Agents Chemother. 2017;61(4).
3. Thoma S, Schobert M. An improved *Escherichia coli* donor strain for diparental mating. FEMS Microbiol Lett. 2009;294(2):127-32.
4. Rietsch A, Vallet-Gely I, Dove SL, Mekalanos JJ. ExsE, a secreted regulator of type III secretion genes in *Pseudomonas aeruginosa*. Proc Natl Acad Sci U S A. 2005;102(22):8006-11.
5. Ouahrani-Bettache S, Porte F, Teyssier J, Liautard JP, Köhler S. pBBR1-GFP: a broad-host-range vector for prokaryotic promoter studies. Biotechniques. 1999;26(4):620-2.
