# Supplementary material for: Plant-Derived Catechols Are Substrates of TonB-Dependent Transporters and Sensitize Pseudomonas aeruginosa to Siderophore-Drug Conjugates
Source: mBio. 2022 Jun 30;13(4):e01498-22. doi: 10.1128/mbio.01498-22 (PMC9426570; doi:10.1128/mbio.01498-22)
Supplement: TABLE S4 [file mbio.01498-22-s0007.pdf]

TABLE S4. Primers used in this study

Primer	Sequence (5' - 3')	Characteristic
pirR-Hind	ACACAAGCTTCCAGTTCGGTGGACATGCT	*pirR* deletion
pirR-Bam1	ACACGGATCCTGCCATTGAGGTGCTGGAAC	*pirR* deletion
pirR-Bam2	ACACGGATCCAAGGCGAGACCCTGACCAAG	*pirR* deletion
pirR-Eco	ACACGAATTCACGAACTCGGCGGGAAGAC	*pirR* deletion
pirS-Hind	ACACAAGCTTCTGCCGGTGATCCTGATGTC	*pirS* deletion
pirS-Bam1	ACACGGATCCTTCGGCGGACAGGTAGTAACC	*pirS* deletion
pirS-Bam2	ACACGGATCCGGTGGCTTTGCCTGGAAGAC	*pirS* deletion
pirS-Eco	ACACGAATTCTCGGCGGTGATGATGGATAC	*pirS* deletion
pirR-Bam	ACACGGATCCCGAAGCGGAAAGATCGTGAA	*pirR* overexpression
pirR-Hind	ACACAAGCTTTAGCAGGAAGAACGCCACCA	*pirR* overexpression
pirS-Bam	ACACGGATCCTCTGGGGCAAGGGTTACGTC	*pirS* overexpression
pirS-Hind	ACACAAGCTTAGGAGATCGGCACGGATGAT	*pirS* overexpression
pirAp-Kpn	ACACGGTACCAGGCGCAGGTGCCTTTACAT	*pirA* promoter cloning
pirAp-Bgl	ACACAGATCTAATTGGGGGTACATGCGTGA	*pirA* promoter cloning
ppiuA-Bam	ACACGGATCCGATGGCAGAAGCCAGCAGAC	*piuA* promoter cloning
ppiuA-Kpn	ACACGGTACCGGTGAAGATGGCGGGAATATG	*piuA* promoter cloning
pirA1	TACTTCAAGCGCGAGAACAACA	qPCR
pirA2	CCAGTTCGAGGTTACGGTTACG	qPCR
piuA1	AACAAGACCGATTCGGACGAT	qPCR
pirA2	GTGCCGTTGTTGTTCTGGGTA	qPCR

Restriction sites used for cloning are underlined
